# Changes in the leaf proteome profile of *Withania somnifera* (L.) Dunal in response to *Alternaria alternata* infection

**DOI:** 10.1371/journal.pone.0178924

**Published:** 2017-06-02

**Authors:** Varinder Singh, Baldev Singh, Robin Joshi, Puneet Jaju, Pratap Kumar Pati

**Affiliations:** 1 Department of Biotechnology, Guru Nanak Dev University, Amritsar, Punjab, India; 2 Biotechnology Division, CSIR-Institute of Himalayan Bioresource Technology, Palampur, Himachal Pradesh, India; 3 Field Application Specialist - Life Science Group, Bio-rad Laboratories (India) Pvt. Ltd, Bangalore, India; Universita degli Studi di Siena, ITALY

## Abstract

*Withania somnifera* is a high value medicinal plant which is used against large number of ailments. The medicinal properties of the plant attributes to a wide array of important secondary metabolites. The plant is predominantly infected with leaf spot pathogen *Alternaria alternata*, which leads to substantial biodeterioration of pharmaceutically important metabolites. To develop an effective strategy to combat this disease, proteomics based approach could be useful. Hence, in the present study, three different protein extraction methods tris-buffer based, phenol based and trichloroacetic acid-acetone (TCA-acetone) based method were comparatively evaluated for two-dimensional electrophoresis (2-DE) analysis of *W*. *somnifera*. TCA-acetone method was found to be most effective and was further used to identify differentially expressed proteins in response to fungal infection. Thirty-eight differentially expressed proteins were identified by matrix assisted laser desorption/ionization time of flight-mass spectrometry (MALDI TOF/TOF MS/MS). The known proteins were categorized into eight different groups based on their function and maximum proteins belonged to energy and metabolism, cell structure, stress and defense and RNA/DNA categories. Differential expression of some key proteins were also crosschecked at transcriptomic level by using qRT-PCR and were found to be consistent with the 2-DE data. These outcomes enable us to evaluate modifications that take place at the proteomic level during a compatible host pathogen interaction. The comparative proteome analysis conducted in this paper revealed the involvement of many key proteins in the process of pathogenesis and further investigation of these identified proteins could assist in the discovery of new strategies for the development of pathogen resistance in the plant.

## Introduction

Two-dimensional electrophoresis (2-DE) is one of the most widely used techniques employed for the study of comparative analysis of protein expression changes during different developmental phases, various biotic and abiotic stress responses and investigation of protein polymorphism [1]. At present, 2-DE is routinely used for synchronous quantitative expression profiling of big sets of complex protein mixtures such as entire cellular lysates. One of the main feature of 2-DE alongside mass spectroscopy (MS), is the capacity to investigate many proteins simultaneously [2]. Sample preparation is the most crucial aspect of successful 2-DE experiment [3]. As Compared to animal tissues, the sample preparation in plants becomes even more challenging because of presence of numerous compounds which have interference effect during extraction like phenolic compounds, organic acids, proteases, polysaccharides, pigments, inhibitory ions and terpenes [4]. The performance of protein extraction and separation on 2-DE gels can also be severely affected by high levels of interfering compounds, like primary and secondary metabolites. Phenolics can cause streaking and generate artefactual spots if they get oxidized by phenoloxidases and peroxidases enzymes. Similarly, polysaccharides, lipids and pigments can also cause severe interference in 2-DE gels [5]. Keeping the above problems in mind, many techniques for the plant sample processing have been suggested [6].

*Withania somnifera* has gained a significant attention for its various medicinal properties and extensive use in Indian, Unani and African systems of conventional medicine [7]. It is known for its anti-stress, immuno-modulatory, cardioprotective, anti-inflammatory, anti-aging, antioxidant and anti-tumour activities [8]. The therapeutic properties of *W*. *somnifera* are linked to the presence of a vast variety of secondary metabolites like alkaloids, withanolides, glycowithanolides, sterols, flavanol glycosides and phenolics [9]. The most important withanolides like withanone and withaferin A which are synthesized by the plant have attracted significant attention recently [10–12]. However, it is known that leaves are the major site of synthesis of withaferin-A and withanone whereas, root act as a minor site [13, 14]. Therefore, it is critical to have detailed informative data on protein composition of *W*. *somnifera* leaf tissues to comprehend their contribution in various metabolic system. Moreover, *W*. *somnifera* can be categorized into recalcitrant species because it contains plenty of primary and secondary metabolites which may interfere during the extraction of proteins.

Disease adversely affects the growth, yield and commercial value of plants. Earlier, we have reported that leaf spot disease of *W*. *somnifera* which is caused by *Alternaria alternata* leads to biodeterioration of pharmaceutically important secondary metabolites and human health promoting components [15–16]. Therefore, it is important to develop suitable strategies to protect the plant from diseases by understanding the underlying mechanisms involved in disease establishment process in the host plant. In this context, the identification of proteins involved in plant-pathogen interaction is considered as critical. Over the last few years, proteomics based approaches have been used to identify various important proteins and their modulation during the host-pathogen interactions [17–20]. However, to best of our knowledge there is no report on proteomics study conducted in *W*. *somnifera* plant infected with *A*. *alternata* pathogen. Hence, in the present study, different protein extraction methods (tris, phenol and TCA-acetone) were compared to find their suitability to be further used for downstream proteome studies of healthy and diseased plant tissue. Further, based on the proteomics data, the present paper highlights the functional and potential biological significance of the identified proteins.

## Materials and methods

### Plant material

*W*. *somnifera* seedlings were germinated in earthen pots containing a combination of soil: sand: vermicompost in the ratio of 1: 1: 8. After 30 days, the developed plants were shifted to individual pots. Two months old plants leaves were utilized for protein extraction. All plant tissues were frozen in liquid nitrogen instantly after harvest and stored at -80°C for subsequent experimentation.

### Pathogen inoculation

The causative pathogen was isolated from leaf spot infected plant and was further used for disease induction. *A*. *alternata* spore were scratched from a full grown petri plate with sterile autoclaved distilled water with 0.01% Tween-20 (v/v). The concentration of spore suspension was adjusted to 6×10^5^ spores/ml with the help of a haemocytometer. These spores were sprayed on the healthier plants and pots were held in wet chamber to maintain relative humidity of 75 ± 5% at 25°C. Plants treated in the same way with sterile water and 0.01% Tween-20 (v/v) served as control. Completely extended leaves demonstrating disease symptoms (brown spots) were immediately solidified in fluid nitrogen and kept at -80°C for further experimentations.

### Extraction protocols

#### Tris-buffer based extraction

Total proteins were isolated using tris buffer based method as described by Granier [21] with minor modifications. Each tissue (100 mg) was powdered in liquid nitrogen using mortar and pestle and 1ml of pre-chilled extraction buffer (50 mM tris (pH 7.5), 0.5% Triton X-100, protease inhibitor cocktail (PIC) 15 μl/ml, 2 mM DTT) was added. Solution was incubated on ice for 1 h and followed by centrifugation at 14,000 rpm for 30 min at 4°C. Supernatant was collected in a new tube and proteins were precipitated with 10% TCA-acetone solution (10g trichloroacetic acid dissolved in 100 ml of acetone) by incubating at -20°C for overnight. Further, proteins were pellet down by centrifugation at 12000 rpm for 10 min. Pellet was washed 2–3 times with cold acetone, air dried and stored at -80°C for 2-DE.

#### Phenol based extraction

Phenol based extraction was performed as described by Faurobert et al [4]. One gram of fresh plant tissue was grounded in precooled mortar and pestle with fluid nitrogen and suspended in 3 mL of extraction buffer (500 mM tris-HCl, 700 mM sucrose, 100 mM KCl, 50 mM EDTA), vortexed, and put on a shaker for 10 min under cold conditions. Further, an equivalent volume of tris-buffered phenol was added and the mixture is kept agitated for another 10 minutes at room temperature. Mixture was further centrifuged for 10 minutes at 10000 rpm and upper phenolic phase was dispensed into a new tube. This upper phenol phase was again re-extracted with fresh 3 mL of extraction buffer. The samples were vortexed and centrifuged for 10 min at 4°C and 10000 rpm for phase separation. The phenolic phase was carefully extracted and poured into a new tube. Four volume of 0.1 M ammonium acetate prepared in cold methanol was added to the tubes, shaken by inverting, and incubated overnight at -20°C. Centrifugation was performed to pellet down the proteins (10 min, 12000 rpm at 4°C). Pellet was washed three times with pre-cooled solution of 0.1 M ammonium acetate in cold methanol and once with pre-cooled acetone. Later, the pellet was dried and kept at -80°C for 2-DE.

#### TCA-acetone extraction

TCA-acetone precipitation was done based on Damerval et al. [22] protocol with few minor changes. In precooled mortar and pastel one gram of leaf tissue was powdered with the liquid nitrogen. Around 100–150 mg leaf tissue was precipitated with 2 mL of precipitation solution (10% TCA-Acetone, 0.07% 2-mercaptoethanol). After incubating at -20°C for overnight, solution was centrifuged for 10 min at 14000 rpm. Supernatant was removed and pellet was washed 3 times with washing solution (Acetone with 0.07% 2-mercaptoethanol). Pellet was dried in laminar and kept at -80°C for 2-DE.

### Protein quantification

The isolated protein pellets from the three different protocols outlined above were redissolved in IEF buffer (7 M urea, 2M thiourea, 0.5% Triton X-100, 4% CHAPS, 0.2% Ampholytes, 50 mM DTT). Protein concentration was quantified using the Bradford protein assay using BSA as a standard [23].

### Two-dimensional electrophoresis (2-DE)

For the evaluation studies, 7 cm IPG strips (3–10, 5–8 pH, Readystrip, Bio-Rad, USA) were passively rehydrated overnight with rehydration sample buffer containing 100 μg of isolated protein. Isoelectric focusing (IEF) was carried out on Protean IEF Cell (Bio-Rad, USA) at 20°C. The strips were focused at 250 V for 40 min, 4000 V for 2h with linear voltage ramping, and finally to 10,000 volt hour (Vh) with rapid ramping. Further, for the protein profile studies of healthy and diseased samples, IPG Strips (Bio-Rad, USA) 11 cm, pH 4–7 were used and strips were passively rehydrated for 14h with 200 ug protein. Strips were loaded onto Protean IEF Cell (Bio-Rad, USA) and focused at 20°C with linear voltage amplification at 500 V for 40 min, 8000 V for 2h and upto 28000 Vh with rapid ramping. Following isoelectric focusing, the strips were reduced with 2% DTT in equilibration buffer {6 M urea, 0.05 M tris-Cl pH 8.8, 20% glycerol, 2% sodium dodecyl sulphate (SDS)} for 15 min and alkylated with 2.5% iodoacetamide for 15 min. The second dimensional SDS page was performed using 12% polyacrylamide. Additional 0.5% of agarose containing 0.1% bromophenol blue (tracking dye) was also used to seal the gel. Electrophoresis was done at constant volt (100 V) for approximately 2 hours in tris glycine-SDS running buffer until the dye (bromophenol blue) reaches the bottom of the gel.

### Gel staining, imaging and statistical analysis

Two replicates of 2-D gels were stained overnight in 0.1% (w/v) Coomassie brilliant blue G-250 (Sigma-Aldrich, cat. no B0770), destained and stored in 5% acetic acid at 4°C for further analysis [24]. The 2-DE gels were imaged using Molecular Imager Gel Doc XR System (Bio-Rad, USA) and were analyzed using PDQuest 8.0.2 software (Bio-Rad, USA). The spots were detected automatically and manual spot addition and removal was also done if required. Molecular masses were determined by running standard protein markers (Bio-Rad, USA) and isoelectric point (pI) was estimated using PDQuest 8.0.2 software (Bio-Rad, USA). A change of at least 1.5-fold spot intensity, increase or decrease, was considered up- and down-accumulation, respectively [25]. Statistical analysis was done with Assistat version 7.7 beta software [26].

### In gel analysis and mass spectroscopy analysis

Spots of interest were excised from the gel manually and further processed as described by [27]. Briefly, excised gel pieces were washed with deionized water and distained with 100 mM NH_4_HCO_3_/50% acetonitrile for 15 min at room temperature. The de-stained gel pieces were again washed with deionized water and dehydrated in100 μl of acetonitrile and vacuum centrifuged. The supernatant was discarded and shrinked gel pieces were digested with 25 μl of freshly prepared enzyme/ trypsin solution (25mM NH_4_HCO_3_ with 5ng/μl of trypsin (Sigma, USA) for overnight at 37°C. The digested gel slices were extracted with extraction buffer (50% trifluoroacetic acid/50% acetonitrile) at room temperature. The digested samples were subjected to mass spectrometric analysis using UltrafleXtreme TM 4 MALDI TOF/TOF system (Bruker Daltonics, Germany), which was calibrated with a mass standard starter kit (Bruker, Germany) and a standard tryptic BSA digest (Bruker, Germany). Mass data was recorded in positive ionization mode and protein identification was performed by searching acquired MS and MS/MS data in non-redundant protein sequence database (Swissprot) using MASCOT search engine version 3.5 (Matrix science: www.matrixscience.com), with the following parameters: parent ion mass tolerance and MS/MS mass tolerance at 100 ppm, peptide mass tolerance ± 1.2 Da, mass values-monoisotopic, peptide charge state-1+, max missed cleavages-1, variable modifications-oxidation of methionine (M) and carbamidomethylation of cysteine (C) was considered as fixed modification, enzyme used as trypsin, taxonomic group were *Viridiaplantae* and the significance threshold was set to *p* <0.05. Protein scores greater than 58 were considered significant (p<0.05).

### Gene specific qRT-PCR

Total RNA was extracted from healthy as well as diseased plant leaves using Trizol reagent (Invitrogen, USA) according to the manufacturer’s guidelines. Five μg of RNA was treated with RNase free DNase (Sigma-Aldrich, USA) to eliminate any DNA contamination and was subsequently purified. Quantification of RNA samples was done using NanoDrop (Thermo Scientific, USA) and first strand cDNA was synthesized using cDNA synthesis kit (Bio-Rad, USA) as per their guidelines.

Primers used for the qRT-PCR analysis were designed from available sequences of selected genes at NCBI database. The PrimerQuest software of Integrated DNA Technologies (http://www.idtdna.com/Primerquest/Home/Index) was used for primer designing under default parameters and were further validated using Primer-BLAST (http://www.ncbi.nlm.nih.gov/tools/primer-blast/) feature. All information regarding primer sequences used in the qRT-PCR analyses and their accession number is presented in S1 Table. Real-time PCR was performed using SYBR Green detection chemistry and run in triplicate with the StepOne Plus machine (Applied Biosystems, USA). Reactions were prepared in 96-wells plates each well containing volume of 20 μl: 10 μl of 2X SYBR Green Master (Applied Biosystems, USA), 1 μl of each primer (1 μM) and 1 μl of template (50 ng). PCR without any template was also conducted as negative control for each primer pair. The qRT-PCR parameters were as follows: initial denaturation step of 95°C for 10 min, followed by 40 cycles of denaturation at 95°C for 15s, annealing and extension at 60°C for 60s. Melt curve analysis was also done ranging from 60°C to 90°C. Baseline and threshold cycles (Ct) were automatically calculated by the Applied Biosystems Software. Data were analysed using the StepOne software and Expression suite (Applied Biosystems: http://www.lifetechnologies.com/in/en/home/technical-resources/software-downloads/expressionsuitesoftware.html). Transcript levels (fold changes) were calculated using ΔΔCt method and normalized against cyclophilin endogenous gene (*CYP*) [28].

## Results

### Comparison between different protein extraction methods

**Fig 1:**
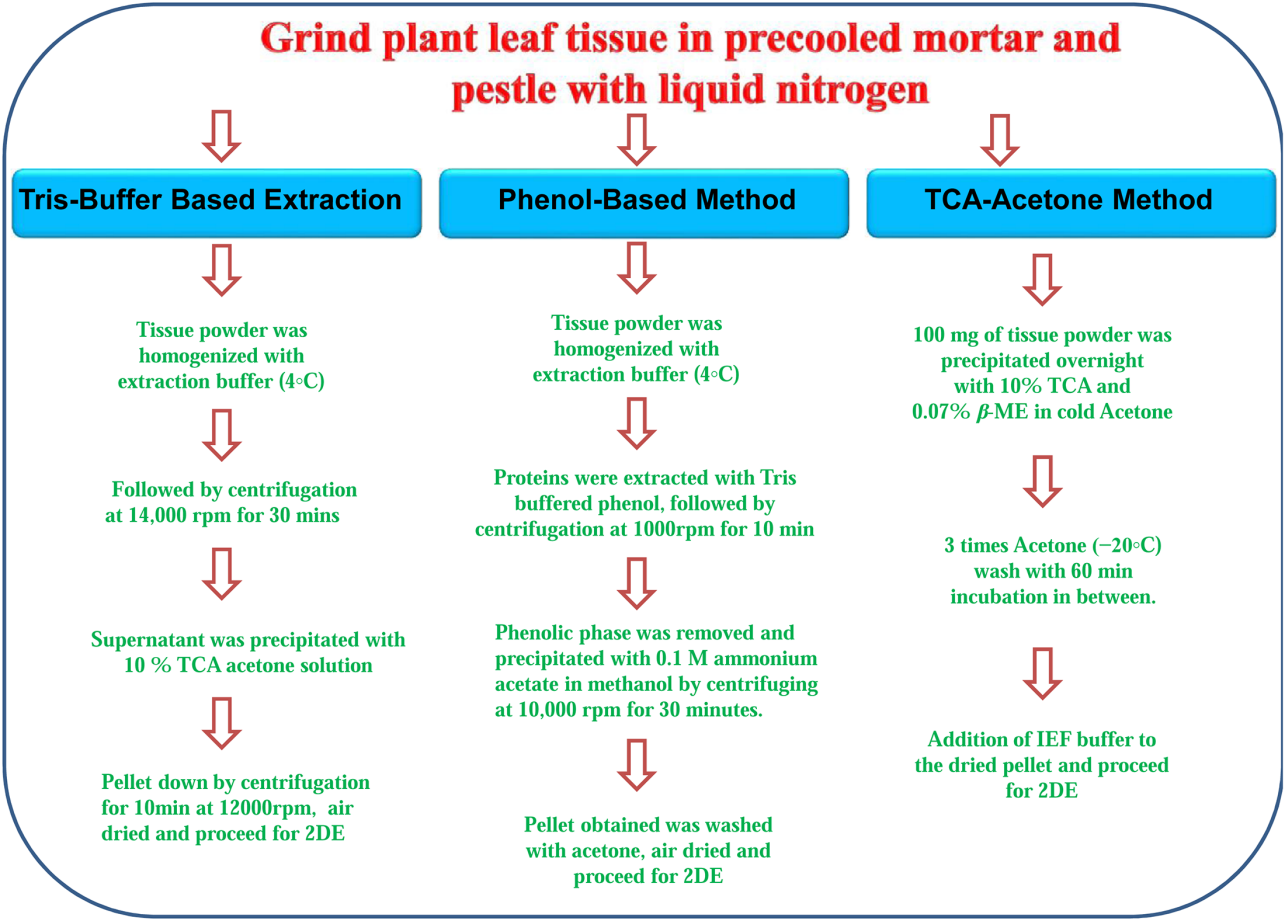
Schematic representation of extraction of protein from *W*. *somnifera* leaves using tris buffer based, phenol based and TCA-acetone based extraction protocols.

In the present study, leaf proteins were isolated using three different protocols (tris Based, phenol based and TCA-acetone based), and were further segregated by 2-DE. Flow diagram presented in Fig 1 described the basic steps involved in the process of protein extraction. The quantitative comparison showed that the phenol based method and TCA-acetone method provided highest protein yields as compared to tris buffer based extraction protocols (Table 1). The concentration of extracted protein was 2.3 ± 0.42 mg/g (tris) 5.0 ± 0.21 mg/g (Phenol) and 4.9 ± 0.13 mg/g (TCA-acetone).

**Table 1:** Features associated with the protein yield and significance of protein spots selected for the identification on 2-DE.

Extraction methods	Protein yield (mg/gm ±SD^a^)	Average number of protein spots ±SD^a^ (3–10 pH strip)	Average number of protein spots ±SD^a^ (5–8 pH strip)
Tris based	2.3 ± 0.42^b^	293±11^b^	311±8^b^
Phenol based	5.0 ± 0.21^c^	537±28^c^	393±23^c^
TCA acetone based	4.9 ± 0.13^c^	670±16^d^	480±22^d^

^a^ Values followed by a different letter (b, c, d) are statistically significant (ANOVA test, p<0.05).

**Fig 2:**
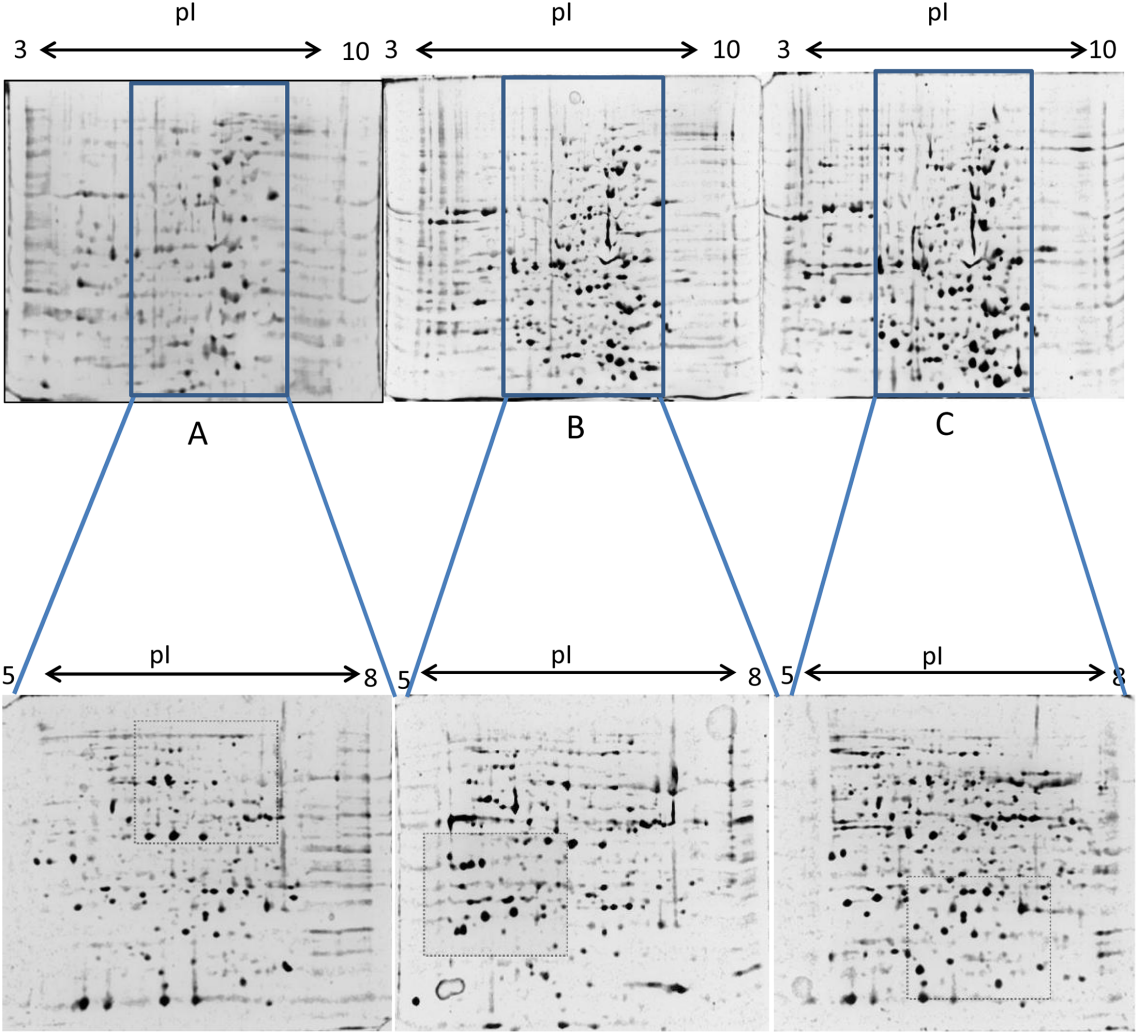
2-DE analysis of proteins from *W*. *somnifera* leaves extracted using the three protocols under evaluation. (A) Tris buffer (B) Phenol and (C) TCA-acetone. Upper panel showed the proteins were separated on a 3–10 nonlinear pH strips. Lower panels are the gel photographs showing better resolution after re-analysis of the same proteins within the pH 5–8 range. Regions of the gels that show particularly different distributions of protein spots when comparing extraction methods are highlighted by rectangular dashed boxes.

Hundreds of protein spots per gel for each leaf tissue were resulted from three different methods. While using 3–10 pH range strips, we observed that majority of the spots were in the range of 5 to 8 pH (Fig 2). The broad pH range (3.0–10.0 IPG strips) were found to generate sufficient number of spots, though the gels were little compromised in protein segregation and resolution. Considering this, and in order to increase the resolution, IEF was performed using 5–8 pH linear range strips. The use of middle pH range (5.0–8.0 IPG strip) revealed a significantly better resolution (Fig 2) and clear spots were observed. Variable spot pattern was observed among separated proteins by the three methods as outlined by dashed boxes in Fig 2. Among the protocols under evaluation, variability was observed for the number of spots obtained. From tris based extraction about 293 ± 11 and 311 ± 8 protein spots could be resolved on 2-D gels using 3–10 and 5–8 pH strips, respectively. In case of phenol extraction method, 537 ± 28 spots for 3–10 pH strips and 393 ± 23 spots for 5–8 pH strips were observed. TCA-acetone method resulted in 670 ± 16 and 480 ± 22 detectable protein spots when 3–10 and 5–8 pH strips were used, respectively (Table 1).

**Fig 3:**
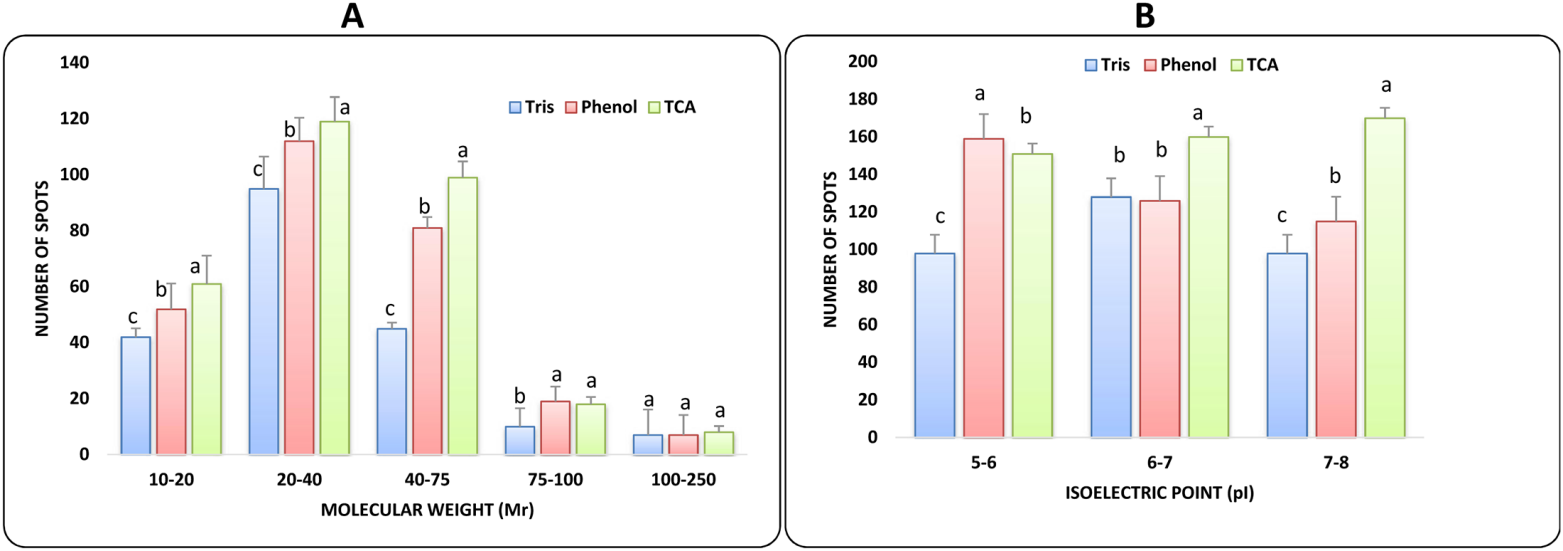
Differential spot distribution of proteins extracted by tris based, phenol based and TCA-acetone based method. According to (A) molecular weight ranges (Mr) and (B) isoelectric point (pI). Bars with different letters within each range correspond to statistically significant differences (ANOVA, p<0.05).

We also calculated the number of spot along the range of molecular weight (M_r_) and isoelectric points (pI). The TCA-acetone protocol constantly gave statistically (P ≤ 0.05) greatest number of spots along isoelectric points (pI) and Molecular weight (M_r_) gradients (Fig 3). We can notice that spots generated by TCA-acetone extraction are significantly more abundant (P ≤ 0.05) in the 10 to 75 kDa M_r_ ranges. Phenol based extraction protocol gave more spots in higher molecular weight range (100–250 kDa). However, tris based method showed low number of spots in all ranges of molecular weight proteins. Further, distribution of proteins according to their isoelectric point (pI) was also analyzed and it was found that maximum spots were distributed in the range of 6 to 7 pI. TCA method gave maximum number of spots in 6–7 and 7–8 pI range as compare to tris and phenol based methods (Fig 3). While phenol based method resolve greater number of spots only in acidic pI ranges.

### Proteomics analysis

**Fig 4:**
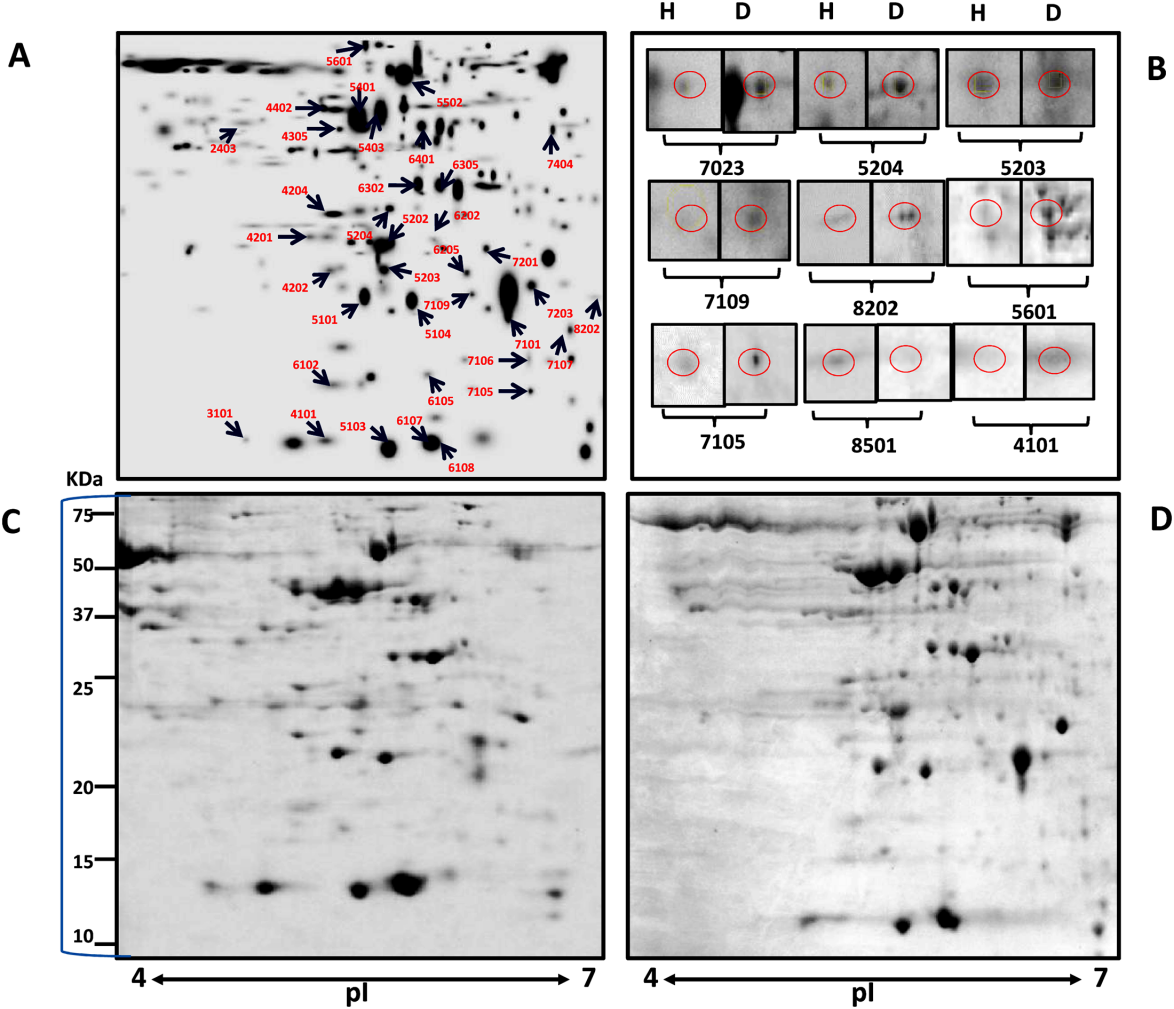
2-DE analysis of differentially extracted proteins from *W*. *somnifera* in response to *A*. *alternata* infection. (A) Master gel Image showing different protein spots selected for MALDI. (B) Changes in the expression pattern of proteins that were selected for quantitative gene expression analysis. The positions of differentially expressed proteins are indicated by circles. (C-D) Representative 2-DE protein profiles of healthy and diseased leaves samples. Proteins were separated over the pI range 4–7 in the first dimension and on 12% SDS-polyacrylamide gels in the second dimension. The gels were stained by colloidal Coomassie brilliant blue G-250. Spot volumes were normalized and determined using PDQuest software.

After the evaluation of different extraction methods, TCA-acetone method was further used to isolate proteins from healthy and diseased plant samples. As shown in the gel images of healthy and infected leaf samples (Fig 4), several protein spots (SSPs) were detected in response to infection during 2-DE analysis. The total number of spots revealed were approximately 243 in healthy and 273 in diseased gels (S2 Table). Out of these, 52 spots were found to be of altered intensity, thus were excised and used for MALDI TOF/TOF MS/MS. Among these differential spots, 38 spots were successfully identified, hence were further analyzed. The list of the proteins which were successfully identified at significant level of confidence by MS/MS is shown in Table 2. For the mass spectrometry analysis each selected spot in Fig 4 was taken from gels of both healthy and infected gels. The results of MS analysis are given in S3 Table. Average fold change was calculated from the spot intensity ratios in comparison to healthy sample.

**Table 2:** Proteins identified by MALDI-TOF/TOF-MS/MS analysis after 2-DE gel separation of proteins from healthy and diseased leaf spot samples of *W*. *somnifera*.

Spot No^a^	Protein Name^b^	Accession number	Reference organism	Mr/pI (Theoretical)	Mr/pI (Experimental)	Score	Coverage	Category
5104	Ferredoxin-NADP reductase	FNRR1_ARATH	*Arabidopsis thaliana*	42.71/8.77	14.39/5.70	68	22	Photosynthesis
6305	Oxygen-evolving enhancer protein	PSBO_HELAN	*Helianthus annuus*	34.48/5.40	44.47/5.83	64	31	Photosynthesis
6302	Oxygen-evolving enhancer protein	PSBO_POPEU	*Populus euphratica*	10.66/5.36	29.70/5.74	67	52	Photosynthesis
4305	NAD(P)H-quinone oxidoreductase subunit	NDHJ_DIOEL	*Dioscorea elephantipes*	18.82/6.19	42.15/5.41	76	41	Photosynthesis
6102	NAD(P)H-quinone oxidoreductase subunit J	NDHJ_DIOEL	*Dioscorea elephantipes*	18.82/6.19	5.87/5.79	75	35	Photosynthesis
5101	Probable serine/threonine-protein kinase NAK	NAK_ARATH	*Arabidopsis thaliana*	43.73/9.28	14.91/5.51	66	19	Energy and metabolism
7201	Adenylate isopentenyl transferase	IPT8_ARATH	*Arabidopsis thaliana*	37.58/8.18	12.97/6.09	74	29	Energy and metabolism
5401	Lipoyl synthase	LIAS_MEDTR	*Medicago truncatula*	42.34/8.75	47.12/5.49	99	32	Energy and metabolism
6202	Lipoyl synthase	LISC_RICCO	*Ricinus communis*	40.73/7.59	23.77/5.81	63	25	Energy and metabolism
5502	ATP synthase subunit beta	ATPB_NICBI	*Nicotiana bigelovii*	53.54/5.09	61.47/5.68	87	37	Energy and metabolism
6105	ATP synthase epsilon Chain	ATPE_ATRBE	*Atropa belladonna*	14.58/5.18	8.90/5.77	82	45	Energy and metabolism
7106	Acyl-[acyl-carrier-protein] desaturase	STAD2_ARATH	*Arabidopsis thaliana*	47 14/5.87	10.02/6.18	84	25	Energy and metabolism
7105	Probable-3-ketoacyl-CoA synthase	KCS14_ARATH	*Arabidopsis thaliana*	52.16/9.18	7.97/6.19	66	19	Energy and metabolism
4106	6-phosphofructokinase	K6PF4_ARATH	*Arabidopsis thaliana*	58.88/8.46	8.03/5.32	70	22	Energy and metabolism
5403	Actin-54	ACT3_TOBAC	*Nicotiana tabacum*	37.52/5.66	47.16/5.58	59	15	Cell structure
8202	Expansin B13	EXB13_ORYSJ	*Oryza sativa subsp*. *Japonica*	24.65.5.55	15.22/6.53	83	39	Cell structure
6401	Vacuolar protein sorting-associated protein	VPS36_ARATH	*Arabidopsis thaliana*	49.23/5.54	44.49/5.76	69	27	Cell structure
4201	Cyclin dependent kinase inhibitor	KRP1_ARATH	*Arabidopsis thaliana*	22.49/5.29	20.89/5.25	66	36	Cell structure
7109	CASP like protein	CSPL9_MAIZE	*Zea Maize*	19.80/9.41	14.08/6.01	65	38	Cell structure
7107	Profilin-3	PROF3_CORAV	*Corylus avellana*	14.29/4.89	9.58/6.38	44	57	Cell structure
7404	Pre-mRNA-processing factor	PR19A_ARATH	*Arabidopsis thaliana*	57.23/6.16	42.85/6.3	71	23	Stress and defense
6108	Putative disease resistance protein	RGA3_SOLBU	*Solanum bulbocastanum*	114.8/6.03	5.56/5.85	74	13	Stress and defense
5601	Putative respiratory burst oxidase homolog protein	RBOHJ_ARATH	*Arabidopsis thaliana*	103.4/9.48	77.91/5.50	60	9	Stress and defense
8501	Glutathione-S-transferase	GSTF2_ARATH	*Arabidopsis thaliana*	24.11/5.92	65.56/6.68	57	23	Stress and defense
7101	Phenylalanine ammonia-lyase	PALY_VITVI	*Vitis vinifera*	46.44/6.12	12.97/6.09	73	28	Stress and defense
5204	Caffeoyl-CoA O-methyltransferase	CAMT1_POPTR	*Populus trichocarpa*	28.01/5.30	25.52/5.61	73	34	Stress and defense
5203	Protein argonaute	AGO1B_ORYSJ	*Oryza sativa subsp*. *Japonica*	124.4/9.55	17.48/5.59	58	9	RNA/DNA
4402	Apurinic endonuclease-redox protein	ARP_ARATH	*Arabidopsis thaliana*	60.62/9.11	51.20/5.33	54	12	RNA/DNA
6205	Pentatricopeptide repeat-containing protein	PP292_ARATH	*Arabidopsis thaliana*	87.76/6.86	18.09/5.94	40	16	RNA/DNA
2403	Protein mago nashi homolog	MGN_ORYSJ	*Oryza sativa subsp*. *Japonica*	18.47/5.84	48.15/4.77	60	26	RNA/DNA
4202	LOB domain-containing protein	LBD26_ARATH	*Arabidopsis thaliana*	17.93/9.39	16.99/5.34	66	50	RNA/DNA
7203	Paired amphipathic helix protein Sin3-like protein	SNL6_ARATH	*Arabidopsis thaliana*	135.8/7.17	15.91/6.19	71	15	RNA/DNA
4101	14-3-3 like protein	1433D_TOBAC	*Nicotiana tabacum*	28.41/4.76	5.74/5.21	53	21	cell Signalling
5103	Probable F-box protein	FB332_ARATH	*Arabidopsis thaliana*	40.17/8.51	5.62/5.60	60	36	cell Signalling
6107	Putative F-box protein	FB169_ARATH	*Arabidopsis thaliana*	26.22/9.19	6.14/5.76	69	18	cell Signalling
4204	Putative cysteine-rich repeat secretory protein	CRR27_ARATH	*Arabidopsis thaliana*	29.38/8.66	23.76/5.35	51	38	Unknown
5202	Uncharacterized protein At4g04980	Y4498_ARATH	*Arabidopsis thaliana*	82.31/5.01	21.20/5.59	60	12	Unknown
3101	Uncharacterized mitochondrial protein	M1010_ARATH	*Arabidopsis thaliana*	13.16/4.59	5.82/4.98	59	30	Unknown

^a^Number corresponds to the 2-DE gel in Fig 2.

^b^Names of the proteins that were obtained via the MASCOT software from the Swissprot database.

**Fig 5:**
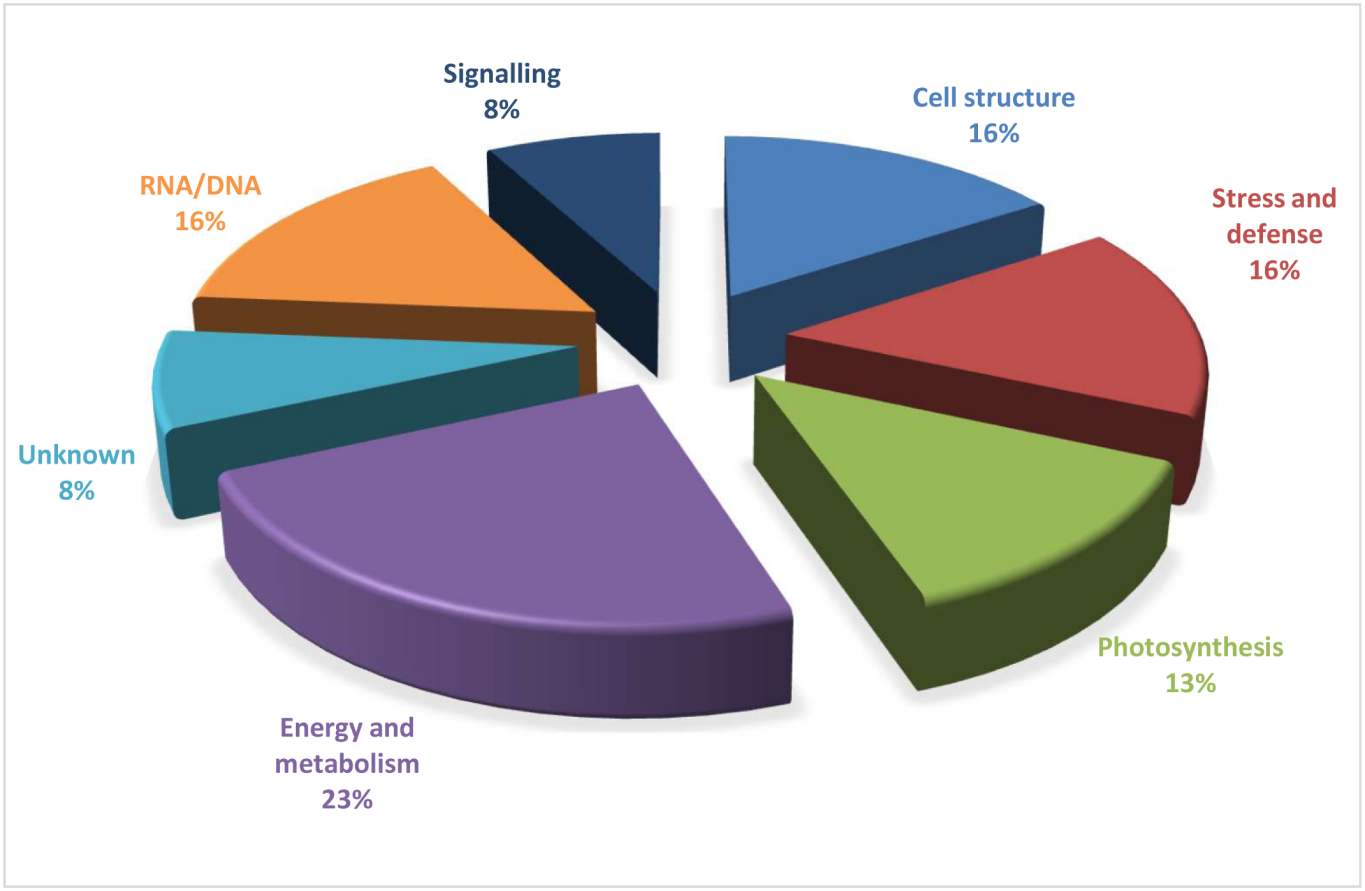
Pie chart showing distribution according to biological function of proteins identified in *W*. *somnifera* in response to fungal infection. This classification is based on KEGG (http://www.kegg.jp/kegg/pathway.html) and on the literature.

The 38 identified proteins were categorized into eight functional groups based upon their biological functions (Fig 5). The identified proteins were found to be associated with various metabolic pathways and functions, such as energy and metabolism (23%), cell structure (16%), stress and defense (16%), photosynthesis (13%), RNA/DNA processing (16%) and signaling (8%). Some proteins had unknown functions but successfully matched in the database were classified as ‘unknown’. The most of the identified proteins belonged to energy and metabolism category. Beside differential spots, the spots which appeared to be present only in one sample were also excised for MALDI analysis. Only five and three spots could be successfully identified from healthy and diseased samples, respectively. From healthy plant sample identified proteins were lipoyl synthase (spot 6202), 6-phosphofructokinase (spot 4106), glutathione-S-transferase (GST) (spot 8501), protein mago nashi (spot 2403) and one uncharacterized mitochondrial protein (spot 3101). From infected leaf sample Profilin-3 (spot 7107), probable Pre-mRNA-processing factor (spot 7404), and 14-3-3 proteins (spot 4101) was identified successfully.

In the present study, twenty-three percent of identified proteins were found to be involved in energy and metabolism. Probable serine/threonine-protein kinase NAK (spot 5101) was up-regulated in response to fungal infection. In diseased samples up-accumulation of proteins was recorded for ATP synthase subunit beta (spot 5202), ATP synthase epsilon chain (spot 6105) and Acyl-[acyl-carrier-protein] desaturase (spot 7106). In contrast, lipoyl synthase (spot 5401), adenylate isopentenyl transferase (spot 7201) and probabale-3-ketoacyl-CoA synthase proteins were found to be down-regulated in diseased sample. Eighteen percent of proteins were found to be related to cell structure and functions. Among these proteins there was not a much significant difference between the spot intensities of healthy and diseased samples except for spot number 4201 (cyclin-dependent kinase inhibitor). There was 2.54 fold down-accumulation of cyclin-dependent kinase inhibitor protein in diseased sample as compared to healthy sample. Interestingly, most of the proteins related to cell structure and functions were down-accumulated in response to fungal infection and it might be due to the necrotic effect of fungal toxin to plant cells.

Five identified proteins were related to photosynthesis category including ferredoxin-NADP reductase protein, two oxygen-evolving enhancer proteins and two NAD(P)H-quinone oxidoreductase subunit proteins. Interestingly, all these proteins were down-accumulated in diseased samples. Among the stress and defense related proteins, spot number 5601 was found to be a putatitve respiratory burst oxidase homolog protein (RBOH) with 1.96 fold up-accumulation in diseased samples. Furthermore, up regulation of caffeoyl-CoA O-methyltransferase (COM) (1.68 fold) was also observed in diseased sample as compared to healthy sample. Spot number 7404 was identified as a Pre-mRNA-processing factor protein which appeared only after fungal infection. Spot number 7101 was identified as Phenylalanine ammonia lyase (PAL) and it was 6.25-fold down-accumulated in diseased sample. Another important protein which disappeared after pathogen inoculation was identified as GST. Spot number 6108 was found to be a putative disease resistance protein which showed slight down-regulation in diseased sample. Approximately 16% of the identified proteins were RNA/ DNA related proteins involving protein argonaute, apurinic endonuclease-redox protein, pentatricopeptide repeat containing protein, protein mago nashi homolog, LOB domain containing protein, and Paired amphipathic helix protein Sin3-like (Sin3-like protein). Protein argonaute (spot 5203), pentatricopeptide repeat containing protein (6205) and Sin3-like protein (7203) showed 1.43, 1.77 and 1.74 fold up-regulation, respectively in the diseased sample. While apurinic endonuclease-redox and mago nashi proteins showed higher expression in healthy samples. Most of the proteins which fall under this group were down-accumulated in diseased sample. The 14-3-3 proteins (spot 4101) which play key functional roles in the signal transduction process by binding to the phosphorylated target, was appeared only in diseased sample gels. Other two probable F-box proteins were down-regulated in diseased sample.

### Correlation between gene expression levels and protein expression

**Fig 6:**
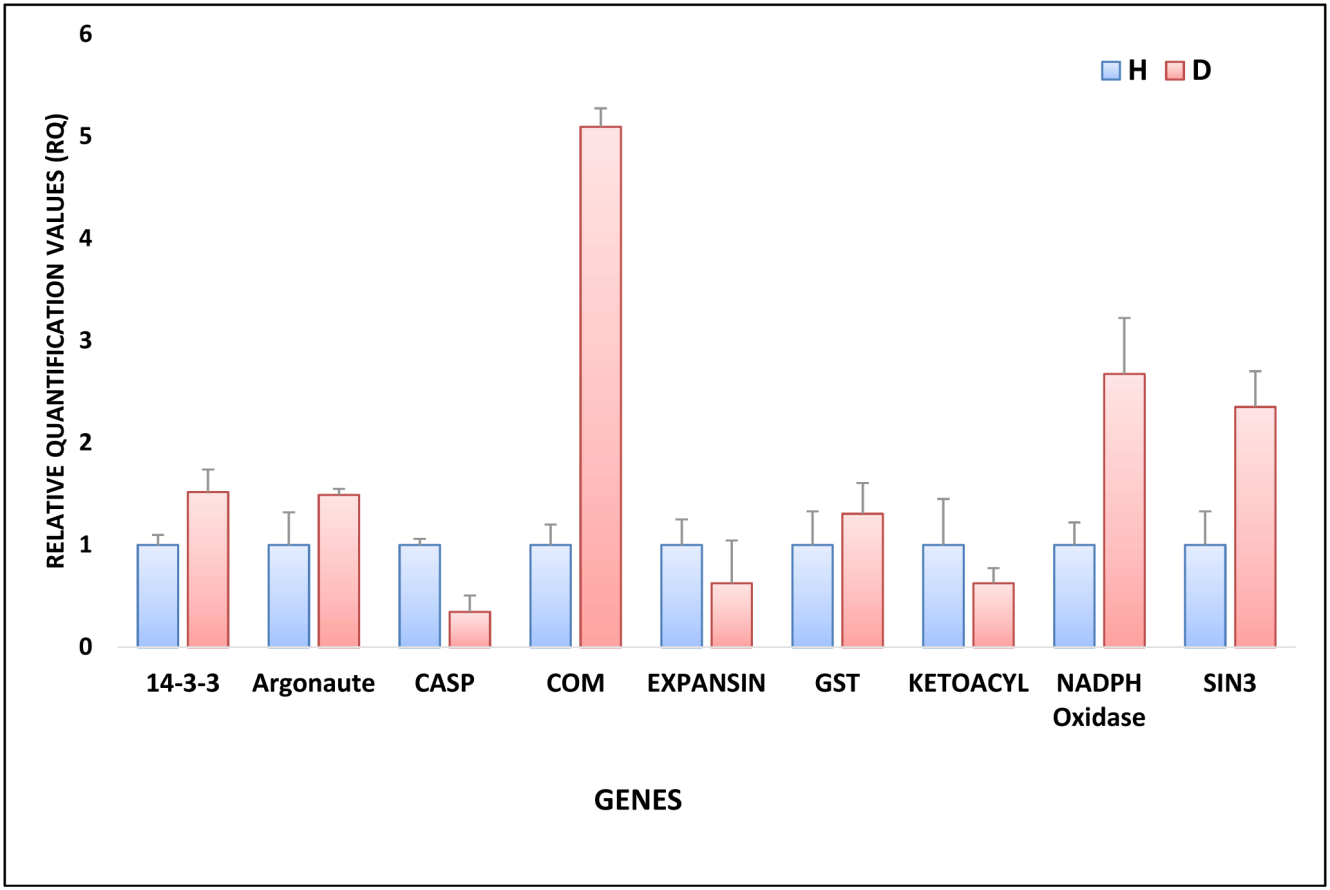
Gene expression levels of selected genes as determined by quantitative RT-PCR in healthy (H) and diseased (D) plant samples. Here 14-3-3, Argonaute, CASP, COM, Expansin, GST, Ketoacyl, NADPH oxidase and SIN3 are 14-3-3 like protein, Argonaute protein, Casparian strip membrane domain proteins, Caffeoyl-CoA O-methyltransferase, Expansin, Glutathione-S-transferase, 3-ketoacyl-CoA synthase, NADPH oxidase, Sin3-like protein. Gene expression data was normalized against expression of cyclophilin (*CYP*) internal control.

To correlate protein expression with corresponding gene expression, the transcript level of 9 genes was analyzed using quantitative RT-PCR (Fig 6). The selected genes were chosen because of their known role in pathogenesis. Proteins selected for qRT-PCR analysis were probable 3-ketoacyl-CoA synthase from energy and metabolism group, expansin protein and Casparian strip membrane domain proteins (CASP) from cell structure group, putative respiratory burst oxidase homolog protein, GST and COM from stress and defense group, protein argonaute from RNA/DNA processing group, Sin3-like protein from RNA/DNA Processing group and 14-3-3-like protein from signaling group. Among these genes, expression of 5 genes was similar to the protein expression while 4 genes showed no correlation between the changes in protein and mRNA levels.

## Discussion

This study was performed with a plant species (*W*. *somnifera*) that contains many interfering compounds and for which no comparative protein extraction studies have been conducted. Comparison was done based on protein yield, gel resolution, focusing of the spots, intensity and number of spots resolved. *W*. *somnifera* contains high levels of flavanol glycosides, sterols and phenolics [9] and various alkaloids [29]. Phenolic compounds may lead to streaking of the gels because they can interact with the proteins either reversibly through hydrogen bonding or irreversibly by oxidation of proteins [30]. The other interfering compounds are carbohydrates which can clog gel pores resulting in extended focusing times, precipitation and streaking in gels. *Withania* is also rich in terpenoids, pigments and various other secondary metabolites which can also produce streaking and charge heterogeneity.

For the comparison of 2D gels, it is recommended that proteins should be well separated, free of smearing, streaking, background staining and reproducible. In the present study, two dimensional protein separation system was used to differentiate the three protein extraction method. Almost equal amount of protein was isolated by both TCA-acetone method and phenol based method. However, it was observed that as compared to other two methods, TCA-acetone method displayed better spots resolution and least streaking. Earlier, Wang *et al*. [31] have also reported the higher protein yield with TCA-acetone method using maize leaf midrib. As compared to other methods, tris based method failed to give significant results both in terms of yield and number of spots. Previously, it has also been reported that direct extraction with tris buffer resulted in lower protein yield [32]. However, among different extraction methods, TCA-acetone based method displayed higher number of protein spots with all range of pH strips. Earlier better performance of TCA-acetone method has also been demonstrated over phenol based method in *Arabidopsis thaliana* [33]. In contrast to TCA-acetone method, tris buffer based extraction method showed poor resolving capabilities and less number of spots. These findings are in accordance to earlier reports, where the suitability of TCA-acetone has been emphasised [32, 34]. While, phenol based extraction system is preferred over other extraction systems due to its high clean-up capacity and hindering the molecular interaction between proteins and other interfering compounds [4]. However, the phenol based method is time consuming and involves chemicals like methanol and phenol which are toxic in nature [4].

We also studied the differential distribution of protein based on their molecular weight (M_r)_ and isoelectric points (pI). In TCA-acetone method most of the proteins were distributed in the range of lower molecular weight whereas, phenol based method favoured the higher molecular range. This finding was further substantiated by Carpentier et al. [35] who reported more number of spots above 25 kDa using phenol based method. On the contrary, TCA-acetone method gave greater number of spots below 25 kDa. The differential distribution of proteins of various molecular weight proteins in phenol and TCA-acetone method has also been reported earlier [36].

Comparative proteome analysis provided critical information regarding the distribution of various proteins and their expression in healthy and diseased leaf samples. These proteins were categories into eight different groups and their associations in the process of defense response were analysed. It was observed that proteins such as probable serine/threonine-protein kinase NAK (spot 5101), ATP synthase subunit beta (spot 5202), ATP synthase epsilon chain (spot 6105) and Acyl-(acyl-carrier-protein) desaturase (spot 7106), which are associated with energy and metabolism were up-accumulated in response to fungal infection. Kinases are known to involve in variety of cellular processes and previously their role in defense response has also been established [37]. Similarly, ATP synthase which is linked to oxidative phosphorylation and ATP synthesis also plays critical role in host pathogen interactions [38]. On the other hand, Lipoyl synthase which catalyzes the final step of lipoic acid biosynthesis was down-accumulated in diseased samples. Lipoic acid is important member of non-enzymatic antioxidant system which play a critical role in ROS scavenging as well as recycling of other antioxidants in plants upon exposed to stress [39].

In the present study, all proteins involved in the process of photosynthesis such as Ferredoxin-NADP reductase, Oxygen-evolving enhancer protein, NAD(P)H-quinone oxidoreductase were found to be downregulated in the diseased samples. Earlier, we have reported a decline in the net rate of photosynthesis during leaf spot disease in *W*. *somnifera* [19]. The leaf spot disease which is caused by *A*. *alternata* is a necrophytic fungus and secretes host specific toxins [40]. Hence, the down-regulation of proteins involved in photosynthesis observed in the present work and the net decrease in the photosynthetic activity reported earlier could be attributed to the process of pathogenesis.

In the present investigation, the stress responsive protein respiratory burst oxidase homolog (RBOH) and COM was found to be up-regulated in diseased sample compared to healthy samples. RBOHs are calcium-dependent NADPH oxidases that generates superoxide ions and implicated in versatile roles in plants [41]. Previously, dual role of the *AtRbohD* in response to *Alternaria* has been reported in *Arabidopsis*, where it triggers cell death in infected cells as well as it simultaneously suppresses the cell-death in neighboring healthy cells [42]. The other stress related protein, COM was found to be up-regulated in response to pathogen attack in *W*. *somnifera*. COM plays a critical role in lignin biosynthesis and hence provide a first line of defence under pathogen attack [43]. In contrast to above proteins, PAL and GST showed decrease expression in diseased sample. PAL is a key regulatory enzyme in phenylpropanoid pathway and involved in the synthesis of many precursor molecules such as flavonoids, phenylpropanoids, and lignins in plants [44]. Overexpression of *PAL* and GST has resulted in disease resistance against several plant pathogens [45–48].

Among RNA/DNA processing proteins spot number 5203 was identified as argonaute protein which is a part of RNA silencing pathway involving small interfering RNAs (siRNA) and microRNAs (miRNAs). Defense regulation mediated by endogenous small RNAs have been reported only in few cases. In *Arabidopsis*, miRNAs have been demonstrated to play important role in plant development as well as abiotic stress tolerance [49, 50]. Earlier, enhanced disease susceptibility of ARGONAUTE4 (AGO4) mutant to *Pseudomonas syringae pv tomato* infection was reported indicating the importance of argonaute proteins for plant disease resistance [51]. In another report, *Cucumber mosaic virus* counteract host defense via CMV 2b protein, which has also been shown to interacts directly with argonaute 1 (AGO1) protein inhibiting AGO1 cleavage activity. These results indicate the importance of AGO1 in defense against CMV infection [52].

Other identified proteins which were more abundant in diseased sample and belong to RNA processing were pentatricopeptide repeat containing protein (PPRPs) and SIN3-like protein. PPRPs has been related to RNA processing, RNA metabolism and posttranscriptional regulation [53]. These proteins are reported to be hyper accumulated in response to fungal infection [54]. SIN3 interacts with site-specific DNA binding factors and modulates the expression of various genes associated with stress response in plants [55].

In the present experiment, the expression of selected proteins (argonaute, COM, 3-ketoacyl-CoA synthase, RBOH, SIN3-like, 14-3-3 protein, CASP, GST and expansin) were also analyzed at transcript level to draw a correlation between transcriptional and translational events. Transcript level expression of argonaute, *COM*, 3-*ketoacyl-CoA synthase*, *RBOH*, *SIN3-like* genes showed a direct correlation with their protein expression profile. However, the transcript level expression of *14-3-3 protein*, *CASP*, *GST* and *expansin* was different from the protein level. This differential expression of these proteins could be due to post-transcriptional or posttranslational modifications [56, 57].

## Conclusion

In the present study, a comparative analysis of different protein extraction methods was conducted in an important medicinal plant. The results demonstrated the higher efficiency of the TCA-acetone based protocol as compared to phenol extraction and tris buffer based method on the basis of protein yield, number of spots, gel resolution and reproducibility. This study will facilitate the future proteomics analysis of this plant. Our investigation on comparative proteome analysis of healthy and diseased sample provides critical information on proteins linked to leaf spot disease initiation in *W*. *somnifera*. The present paper also indicated some novel proteins which are still to be explored for their involvement in the process of pathogenesis.

## Supporting information

S1 Table Details of primers used for quantitative RT-PCR. (DOCX)

S2 Table Protein yield and number of spots observed. (DOCX)

S3 Table MALDI data obtained from MASCOT search. (PDF)
