# Supplementary material for: Changes in the leaf proteome profile of Withania somnifera (L.) Dunal in response to Alternaria alternata infection
Source: PLoS One. 2017 Jun 2;12(6):e0178924. doi: 10.1371/journal.pone.0178924 (PMC5456394; doi:10.1371/journal.pone.0178924)
Supplement: S1 Table — (DOCX) [file pone.0178924.s001.docx]

S1 Table: Details of primers used for quantitative RT-PCR

| ***Gene Abbreviation*** | **Gene Definition** | **Accession Number** | **Primer Pair**  **(5’Forward - 3’/5’-reverse-3’)** | **Product Size** |
| --- | --- | --- | --- | --- |
| *NADPH oxidase* | NADPH oxidoreductase | AB372264.1 | \| TAGCACTCGTCTTGCGATTC/ \| \| --- \| \| CTTTGGAGGCCGAATAGATAGG \| | 122 |
| *Expansin* | Expansin-like protein | DQ178133.1 | \| GGTACGATGGCAAGTGGATT / \| \| --- \| \| GGAACAACCTTCTTGAGCAATG \| | 113 |
| *Sin3-like* | Sin3-like | NM_100919.4 | \| TGAGGTCTACGAAGAGGTTACT / \| \| --- \| \| CTTTGTCGAGGGAGCTGATT \| | 106 |
| *GST* | Glutathione-S-transferase | Solyc01g099590.2.1 | \| TCGGTGGTAGAGGGATTACA / \| \| --- \| \| GGAACCAATAAGCAATCCAACC \| | 115 |
| *Ketoacyl* | 3-ketoacyl-CoA synthase | XM_009760570.1 | \| CACACGGTTCGAACCCATA / \| \| --- \| \| TTTAGAGCATCCCCAGCTAC \| | 131 |
| *14-3-3* | 14-3-3 like protein | XM_006353123.1 | \| TTGCTGAACTGGACACTTTG/ \| \| --- \| \| GTCCATCTGCTCCTGCATATC \| | 113 |
| *COM* | Caffeoyl-CoA  O-methyltransferase | XM_006339819.1 | \| ACACCCTTGGAACCTYATGAC / \| \| --- \| \| CATGGCAGTAGCAAGMAGAGA \| | 133 |
| *Argonaute* | Argonaute 1-like protein | XM_009607958.1 | \| CTCGTTCCGTCTCCATTGTT / \| \| --- \| \| GACTGATCCACTGTCAGATGTC \| | 101 |
| *CASP* | CASP Like Protein | XM_009607958.1 | TCACTGGTTCAAGTTATAAGG/  TCAAGTAGGCCATCAACTGAT | 112 |
| *CYP* | Endogenous Control | AF126551.1 | \| AGGTGTTGGAAAGATGGGTAAG/ \| \| --- \| \| TCACCTCCTTGACACATGAAC \| | 87 |
