# Supplementary material for: Changes in the leaf proteome profile of Withania somnifera (L.) Dunal in response to Alternaria alternata infection
Source: PLoS One. 2017 Jun 2;12(6):e0178924. doi: 10.1371/journal.pone.0178924 (PMC5456394; doi:10.1371/journal.pone.0178924)
Supplement: S2 Table — (DOCX) [file pone.0178924.s002.docx]

S2 Table: Protein yield and number of spots observed.

| Sample | Protein yield (*m*g/gm ±SD) | Average number of spots ±SD | Number of spots selected  for the analysis | Number of  spots identified |
| --- | --- | --- | --- | --- |
| Healthy | 4.0 ± 0.28 | 211±27 | 52 | 38 |
| Disease | 3.6 ± 0.23 | 232±19 |  |  |
