# Supplementary material for: Changes in the leaf proteome profile of Withania somnifera (L.) Dunal in response to Alternaria alternata infection
Source: PLoS One. 2017 Jun 2;12(6):e0178924. doi: 10.1371/journal.pone.0178924 (PMC5456394; doi:10.1371/journal.pone.0178924)
Supplement: S3 Table — (PDF) [file pone.0178924.s003.pdf]

Protein View: FNRR1_ARATH

Ferredoxin--NADP reductase, root isozyme 1, chloroplastic OS=Arabidopsis thaliana GN=RFNR1 PE=2 SV=2

**Database:**	SwissProt
**Score:**	68
**Expect:**	0.0049
**Nominal mass (M_r):**	42712
**Calculated pI:**	8.77
**Taxonomy:**	Arabidopsis thaliana

Sequence similarity is available as an NCBI BLAST search of FNRR1_ARATH against nr.

Search parameters

**Enzyme:**	Trypsin: cuts C-term side of KR unless next residue is P.
**Fixed modifications:**	Carbamidomethyl (C)
**Variable modifications:**	Oxidation (M)
**Mass values searched:**	6
**Mass values matched:**	6

Protein sequence coverage: 22%

Matched peptides shown in ***bold red***.

```
  1 MALSTTPSQM SVALPTRIDG SSRSMIKVQS ISFTDKSWGP PLLRLDSKSR
 51 SLGVKKRSTI CMSLQQSSKS KVLVTPLELE DPKETPLNLF RPKEPYTATI
101 VSVERIVGPQ APGETCHIVI DHDGNVPYWE GQSYGVIPPG ENPKKPGAPH
151 NVRLYSIAST RYGDSFDGKT ASLCVRRAIY YDPETGKEDP SKAGVCSNFL
201 CNAKPGDKVK ITGPSGKVML LPEDDPKATH IMIATGTGVA PYRGYLRRMF
251 MENVPNFKFD GLAWLFLGVA NSDSLLYDEE FAGYRKDYPE NFRYDKALSR
301 EEKNKKGGKM YVQDKIEEYS DEIFKLLDNG AHIYFCGLKG MMPGIQDTLK
351 RVAEERGESW EQKLTQLRKN KQWHVEVY
```

Unformatted sequence string: 378 residues (for pasting into other applications).

Sort peptides by ◉ Residue Number ○ Increasing Mass ○ Decreasing Mass

Show predicted peptides also

Start - End	Observed	Mr(expt)	Mr(calc)	Delta	M	Peptide
72 - 83	1351.6816	1350.6744	1351.7599	-1.0855	0	K.VLVTPLELEDPK.E
218 - 227	1171.6773	1170.6701	1171.5795	-0.9094	0	K.VMLLPEDDPK.A + Oxidation (M)
259 - 285	3069.2635	3068.2562	3067.4603	0.7959	0	K.FDGLAWLFLGVANSDSLLYDEEFAGYR.K
316 - 325	1272.6581	1271.6508	1271.5921	0.0587	0	K.IEEYSDEIFK.L
326 - 350	2808.3089	2807.3016	2807.3809	-0.0792	1	K.LLDNGAHIYFCGLKGMMPGIQDTLK.R + Oxidation (M)
340 - 350	1189.6841	1188.6768	1189.5835	-0.9067	0	K.GMMPGIQDTLK.R

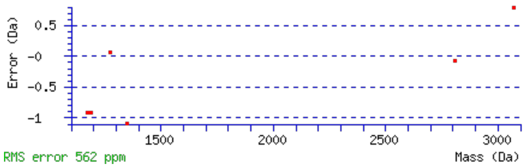

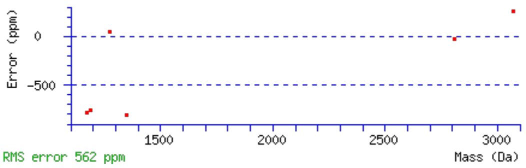

Protein View: PSBO_HELAN

Oxygen-evolving enhancer protein 1, chloroplastic OS=Helianthus annuus GN=PSBO PE=1 SV=1

**Database:**	SwissProt
**Score:**	64
**Expect:**	0.014
**Nominal mass (M_r):**	34487
**Calculated pI:**	5.40
**Taxonomy:**	Helianthus annuus

Sequence similarity is available as **an NCBI BLAST search of PSBO_HELAN against nr**.

Search parameters

**Enzyme:**	Trypsin: cuts C-term side of KR unless next residue is P.
**Fixed modifications:**	Carbamidomethyl (C)
**Variable modifications:**	Oxidation (M)
**Mass values searched:**	8
**Mass values matched:**	6

Protein sequence coverage: 31%

Matched peptides shown in ***bold red***.

1 MAASLQAAAT FMTPTSRVQL KSSPSICKAF GIESTGGSKVS CSLQADLKDF
51 AQKCTDAAKI AGFALATSAL VVSGASAEGS PK**RLTYDEIQ** **SK**TYMEVK**GT**
101 **GTANQCPTIE** **GGVNGFAVKP** **GKYNAQK**FCL EPTSFTVKAE GISKNSAPEF
151 QKTKLMTR**LT** **YTLDEIEGPL** **EVSSDGTIK**F EEK**DGIDYAA** **VTVQLPGGER**
201 **VPFLFTIK**EL VATGKPESFG GNFLVPSYRG SSFLDPKGR**G** **GSTGYDNAVA**
251 **LPAGGR**GDEE ELLKENIKNT AAGKGEITFS VTSSKPETGE VIGVFESIQP
301 SDTDLGAKAP KDVKIQGVWY AQLW

Unformatted sequence string: **324 residues** (for pasting into other applications).

Sort peptides by ◉ Residue Number ○ Increasing Mass ○ Decreasing Mass

Show predicted peptides also

Start – End	Observed	Mr(expt)	Mr(calc)	Delta	M	Peptide
83 – 92	1252.6314	1251.6241	1251.6459	-0.0218	1	K.RLTYDEIQSK.T
99 – 127	2963.3067	2962.2994	2963.4560	-1.1566	1	K.GTGTANQCPTIEGGVNGFAVKPGKYNAQK.F
159 – 179	2281.0610	2280.0537	2279.1420	0.9117	0	R.LTYTLDEIEGPLEVSSDGTIK.F
184 – 200	1760.8255	1759.8183	1759.8741	-0.0558	0	K.DGIDYAAVTVQLPGGER.V
201 – 208	964.5680	963.5608	963.5793	-0.0186	0	R.VPFLFTIK.E
240 – 256	1562.7286	1561.7213	1561.7485	-0.0272	0	R.GGSTGYDNAVALPAGGR.G

No match to: 2808.2181, 3347.5928

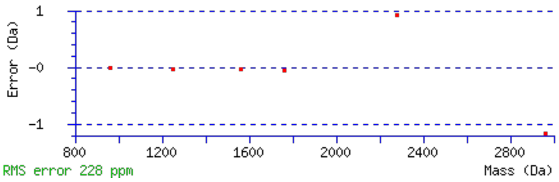

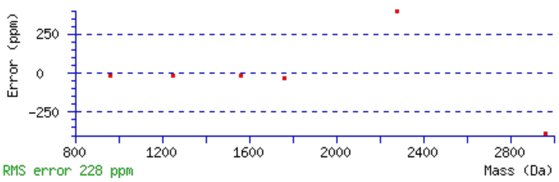

Protein View: PSBO_POPEU

Oxygen-evolving enhancer protein 1, chloroplastic (Fragments) OS=Populus euphratica GN=PSBO PE=1 SV=1

**Database:**	SwissProt
**Score:**	67
**Expect:**	0.0062
**Nominal mass (M_r):**	10664
**Calculated pI:**	5.36
**Taxonomy:**	Populus euphratica

Sequence similarity is available as an NCBI BLAST search of PSBO_POPEU against nr.

Search parameters

**Enzyme:**	Trypsin: cuts C-term side of KR unless next residue is P.
**Fixed modifications:**	Carbamidomethyl (C)
**Variable modifications:**	Oxidation (M)
**Mass values searched:**	4
**Mass values matched:**	4

Protein sequence coverage: 52%

Matched peptides shown in ***bold red***.

```
 1 RLTYDEIQSK AEGINKNSPP DFQKTKLMTR DGIDYAAVTV QLPGGERVPF
51 LFTIKGGSTG YDNAVALPAG GRSKPETGEI IGVFESLQPS DTDLGAKTPK
```

Unformatted sequence string: 100 residues (for pasting into other applications).

Sort peptides by ◉ Residue Number ○ Increasing Mass ○ Decreasing Mass

Show predicted peptides also

Start – End	Observed	Mr(expt)	Mr(calc)	Delta	M	Peptide
1 – 10	1252.6591	1251.6518	1251.6459	0.0059	1	-.RLTYDEIQSK.A
31 – 47	1760.8608	1759.8536	1759.8741	-0.0205	0	R.DGIDYAAVTVQLPGGER.V
48 – 55	964.5832	963.5759	963.5793	-0.0035	0	R.VPFLFTIK.G
56 – 72	1563.7691	1562.7618	1561.7485	1.0133	0	K.GGSTGYDNAVALPAGGR.S

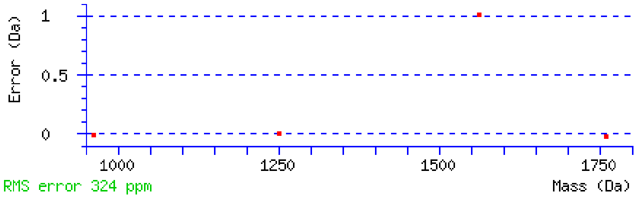

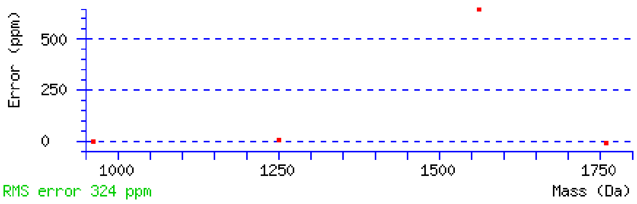

Protein View: NDHJ_DIOEL

NAD(P)H-quinone oxidoreductase subunit J, chloroplastic OS=Dioscorea elephantipes GN=ndhJ PE=3 SV=1

**Database:**	SwissProt
**Score:**	76
**Expect:**	0.00087
**Nominal mass (M_r):**	18820
**Calculated pI:**	6.19
**Taxonomy:**	Dioscorea elephantipes

Sequence similarity is available as an NCBI BLAST search of NDHJ_DIOEL against nr.

Search parameters

**Enzyme:**	Trypsin: cuts C-term side of KR unless next residue is P.
**Fixed modifications:**	Carbamidomethyl (C)
**Variable modifications:**	Oxidation (M)
**Mass values searched:**	6
**Mass values matched:**	6

Protein sequence coverage: 41%

Matched peptides shown in ***bold red***.

```
  1 MQSRLSDWLV KHELVHRSLG FDYQGIETLQ IKTEDWDSIA VISYIYGYNY
 51 LRSQCAYDVA PGGFLASVYH LTRIQYGIDK VEEVCLKVFV SRNNPRIPSV
101 FWIWKSADFQ ERESYDMLGI SYANHPHLKR ILMPESWIGW PLRKDYITPN
151 FYEIQDAR
```

Unformatted sequence string: 158 residues (for pasting into other applications).

Sort peptides by ◉ Residue Number ○ Increasing Mass ○ Decreasing Mass

Show predicted peptides also

Start – End	Observed	Mr(expt)	Mr(calc)	Delta	M	Peptide
5 – 11	861.0230	860.0157	859.4803	0.5354	0	R.LSDWLVK.H
81 – 87	876.9961	875.9888	875.4422	0.5466	0	K.VEEVCLK.V
106 – 129	2807.1723	2806.1650	2807.2973	-1.1323	1	K.SADFQERESYDMLGISYANHPHLK.R
131 – 144	1724.8474	1723.8401	1724.9436	-1.1035	1	R.ILMPESWIGWPLRK.D
131 – 144	1742.8585	1741.8512	1740.9385	0.9127	1	R.ILMPESWIGWPLRK.D + Oxidation (M)
144 – 158	1874.0140	1873.0067	1871.9054	1.1014	1	R.KDYITPNFYEIQDAR.-

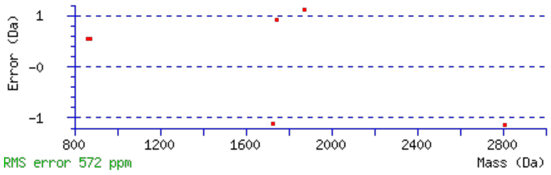

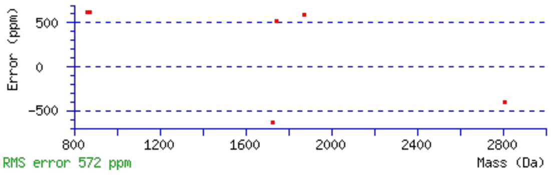

Protein View: NDHJ_DIOEL

NAD(P)H-quinone oxidoreductase subunit J, chloroplastic OS=Dioscorea elephantipes GN=ndhJ PE=3 SV=1

**Database:**	SwissProt
**Score:**	75
**Expect:**	0.0011
**Nominal mass (M_r):**	18820
**Calculated pI:**	6.19
**Taxonomy:**	Dioscorea elephantipes

Sequence similarity is available as **an NCBI BLAST search of NDHJ_DIOEL against nr**.

Search parameters

**Enzyme:**	Trypsin: cuts C-term side of KR unless next residue is P.
**Fixed modifications:**	Carbamidomethyl (C)
**Variable modifications:**	Oxidation (M)
**Mass values searched:**	5
**Mass values matched:**	5

Protein sequence coverage: 35%

Matched peptides shown in ***bold red***.

```
  1 MQSRLSDWLV KHELVHRSLG FDYQGIETLQ IKTEDWDSIA VISYIYGYNY
 51 LRSQCAYDVA PGGFLASVYH LTRIQYGIDK VEEVCLKVFV SRNNPRIPSV
101 FWIWKSADFQ ERESYDMLGI SYANHPHLKR ILMPESWIGW PLRKDYITPN
151 FYEIQDAR
```

Unformatted sequence string: **158 residues** (for pasting into other applications).

Sort peptides by ◉ Residue Number ○ Increasing Mass ○ Decreasing Mass

Show predicted peptides also

Start – End	Observed	Mr(expt)	Mr(calc)	Delta	M	Peptide
1 – 11	1377.5544	1376.5471	1377.7075	-1.1603	1	-.MQSRLSDWLVK.H + Oxidation (M)
5 – 11	861.0104	860.0032	859.4803	0.5228	0	R.LSDWLVK.H
81 – 87	876.9842	875.9769	875.4422	0.5347	0	K.VEEVCLK.V
106 – 129	2807.1607	2806.1534	2807.2973	-1.1439	1	K.SADFQERESYDMLGISYANHPHLK.R
145 – 158	1743.7824	1742.7752	1743.8104	-1.0352	0	K.DYITPNFYEIQDAR.-

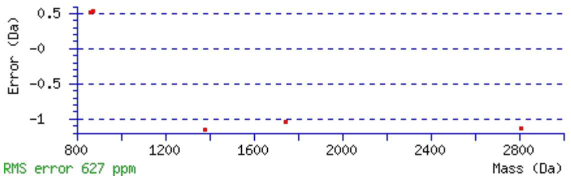

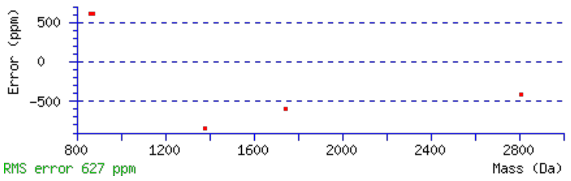

Protein View: NAK_ARATH

Probable serine/threonine-protein kinase NAK OS=Arabidopsis thaliana GN=NAK PE=2 SV=2

**Database:**	SwissProt
**Score:**	66
**Expect:**	0.0089
**Nominal mass (M_r):**	43734
**Calculated pI:**	9.28
**Taxonomy:**	Arabidopsis thaliana

Sequence similarity is available as an NCBI BLAST search of NAK_ARATH against nr.

Search parameters

**Enzyme:**	Trypsin: cuts C-term side of KR unless next residue is P.
**Fixed modifications:**	Carbamidomethyl (C)
**Variable modifications:**	Oxidation (M)
**Mass values searched:**	6
**Mass values matched:**	6

Protein sequence coverage: 19%

Matched peptides shown in ***bold red***.

1 MGGCFSNRIK TDIASSTWLS SKFLSRDGSK **GSSTASFSYM PR**TEGEILQN
51 ANLK**NFSLSE LKSATRNFRP DSVVGEGGFG CVFK**GWIDES SLAPSKPGTG
101 IVIAVKRLNQ EGFQGHREWL AEINYLGQLD HPNLVKLIGY CLEEEHR**LLV**
151 **YEFMTR**GSLE NHLFRRGTFY QPLSWNTRVR MALGAARGLA FLHNAQPQVI
201 YRDFKASNIL LDSNYNAKLS DFGLARDGPM GDNSHVSTRV MGTQGYAAPE
251 YLATGHLSVK SDVYSFGVVL LELLSGRRAI DKNQPVGEHN LVDWARPYLT
301 NKRRLLRVMD PRLQGQYSLT RALKIAVLAL DCISIDAK**SR PTMNEIVKTM**
351 **EELHIQKEAS K**EQQNPQISI DNIINKSPQA VNYPRPSIM

Unformatted sequence string: 389 residues (for pasting into other applications).

Sort peptides by ◉ Residue Number ○ Increasing Mass ○ Decreasing Mass

Show predicted peptides also

Start – End	Observed	Mr(expt)	Mr(calc)	Delta	M	Peptide
31 – 42	1290.7072	1289.6999	1289.5710	0.1289	0	K.GSSTASFSYMPR.T
55 – 66	1351.7304	1350.7231	1351.7095	-0.9864	1	K.NFSLSELKSATR.N
63 – 84	2387.1820	2386.1747	2386.1488	0.0259	1	K.SATRNFRPDSVVGEGGFGCVFK.G
148 – 156	1171.6422	1170.6350	1170.6107	0.0243	0	R.LLVYEFMTR.G
339 – 348	1189.6494	1188.6422	1189.6125	-0.9703	0	K.SRPTMNEIVK.T + Oxidation (M)
349 – 361	1558.7590	1557.7518	1558.7661	-1.0143	1	K.TMEELHIQKEASK.E + Oxidation (M)

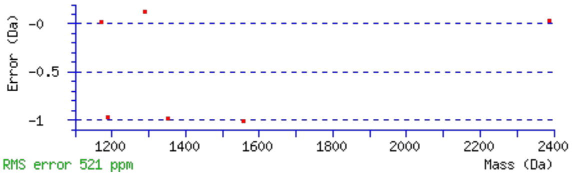

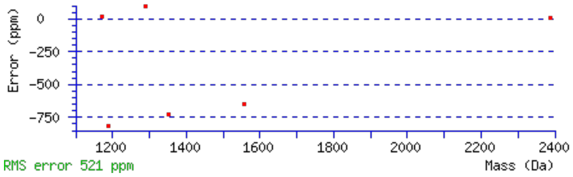

Protein View: IPT8_ARATH

Adenylate isopentenyltransferase 8, chloroplastic OS=Arabidopsis thaliana GN=IPT8 PE=2 SV=1

**Database:**	SwissProt
**Score:**	74
**Expect:**	0.0012
**Nominal mass (M_r):**	37583
**Calculated pI:**	8.18
**Taxonomy:**	Arabidopsis thaliana

Sequence similarity is available as an NCBI BLAST search of IPT8_ARATH against nr.

Search parameters

**Enzyme:**	Trypsin: cuts C-term side of KR unless next residue is P.
**Fixed modifications:**	Carbamidomethyl (C)
**Variable modifications:**	Oxidation (M)
**Mass values searched:**	6
**Mass values matched:**	6

Protein sequence coverage: 29%

Matched peptides shown in ***bold red***.

```
  1 MQNLTSTFVS PSMIPITSPR LRLPPPRSVV PMTTVCMEQS YKQKVVVIMG
 51 ATGSGKSCLS IDLATRFSGE IVNSDKIQFY DGLKVTTNQM SILERCGVPH
101 HLLGELPPDD SELTTSEFRS LASRSISEIT ARGNLPIIAG GSNSFIHALL
151 VDRFDPKTYP FSSETSISSG LRYECCFLWV DVSVSVLFEY LSKRVDQMME
201 SGMFEELAGF YDPRYSGSAI RAHGIHKTIG IPEFDRYFSL YPPERKQKMS
251 EWDQARKGAY DEAVQEIKEN TWRLAKKQIE RIMKLKSSGW DIQRLDATPS
301 FGRSSREIWD NTVLDESIKV VKRFLVKDKV
```

Unformatted sequence string: 330 residues (for pasting into other applications).

Sort peptides by ◉ Residue Number ○ Increasing Mass ○ Decreasing Mass

Show predicted peptides also

Start – End	Observed	Mr(expt)	Mr(calc)	Delta	M	Peptide
28 – 42	1791.7308	1790.7235	1790.7889	-0.0654	0	R.SVVPMTTVCMEQSYK.Q + 2 Oxidation (M)
85 – 95	1307.6730	1306.6657	1306.6551	0.0106	0	K.VTTNQMSILER.C + Oxidation (M)
96 – 119	2706.1347	2705.1275	2705.2755	-0.1481	0	R.CGVPHHLLGELPPDDSELTTSEFR.S
125 – 132	877.0592	876.0519	875.4712	0.5807	0	R.SISEITAR.G
195 – 214	2384.8911	2383.8838	2382.9807	0.9032	0	R.VDQMMESGMFEELAGFYDPR.Y + 2 Oxidation (M)
228 – 245	2201.1019	2200.0947	2199.1000	0.9946	1	K.TIGIPEFDRYFSLYPPER.K

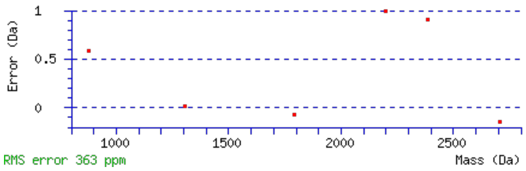

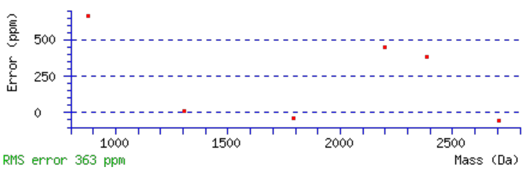

Protein View: LIAS_MEDTR

Lipoyl synthase, mitochondrial OS=Medicago truncatula GN=LIP1 PE=2 SV=1

**Database:**	SwissProt
**Score:**	99
**Expect:**	4.1e-006
**Nominal mass (M_r):**	42342
**Calculated pI:**	8.75
**Taxonomy:**	Medicago truncatula

Sequence similarity is available as an NCBI BLAST search of LIAS_MEDTR against nr.

Search parameters

**Enzyme:**	Trypsin: cuts C-term side of KR unless next residue is P.
**Fixed modifications:**	Carbamidomethyl (C)
**Variable modifications:**	Oxidation (M)
**Mass values searched:**	10
**Mass values matched:**	10

Protein sequence coverage: 32%

Matched peptides shown in ***bold red***.

```
  1 MMYSRFRTVA KNLKSTTKPF SFTTATTTTT VSSSEFPQNL TELRARLARE
 51 SPSLSDFISL KSNNAYSVEV GTKKNPLPKP KWMKESIPGG WKYVQIKKKL
101 RELKLHTVCE EAKCPNMGEC WSGGETGTAT ATIMILGDTC TRGCRFCNVK
151 TSRTPPPPDP DEPTNVAEAI ASWGLDYVVI TSVGRDDLPD QGSSHFTETV
201 QKLKILKPSI LIEALVPDFR GNAECVEKVS KSGLDVFAHN IETVEELQSA
251 VRDHRANFNQ SLDVLRMAKD YAPAGTLTKT SIMLGCGETP DQIVKTMEKV
301 RAAGVDVMTF GQYMRPSKRH MPVSEYITPE AFEKYQTLGM EMGFRYVASG
351 PMVRSSYKAG EFYIKSMIDS DRAVSSQS
```

Unformatted sequence string: 378 residues (for pasting into other applications).

Sort peptides by ◉ Residue Number ○ Increasing Mass ○ Decreasing Mass

Show predicted peptides also

Start – End	Observed	Mr(expt)	Mr(calc)	Delta	M	Peptide
1 – 7	1023.4691	1022.4619	1021.4473	1.0145	1	-.MMYSRFR.T + 2 Oxidation (M)
47 – 61	1663.6923	1662.6850	1661.8988	0.7862	1	R.LARESPSLSDFISLK.S
85 – 97	1503.6427	1502.6355	1503.8085	-1.1731	1	K.ESIPGGWKYVQIK.K
93 – 97	649.9932	648.9859	649.3799	-0.3939	0	K.YVQIK.K
105 – 113	1086.5785	1085.5712	1085.5175	0.0536	0	K.LHTVCEEAK.C
205 – 220	1822.9480	1821.9407	1823.0920	-1.1513	0	K.ILKPSILIEALVPDFR.G
280 – 295	1747.8230	1746.8157	1747.8485	-1.0328	0	K.TSIMLGCGETPDQIVK.T
300 – 318	2111.9953	2110.9880	2112.0609	-1.0728	1	K.VRAAGVDVMTFGQYMRPSK.R
319 – 334	1933.8961	1932.8889	1932.9404	-0.0515	1	K.RHMPVSEYITPEAFEK.Y
346 – 358	1459.6253	1458.6180	1459.7129	-1.0950	1	R.YVASGPMVRSSYK.A + Oxidation (M)

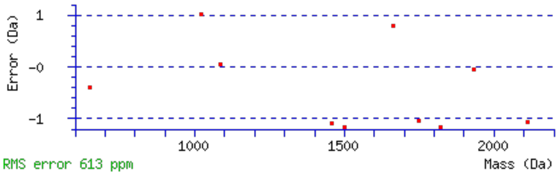

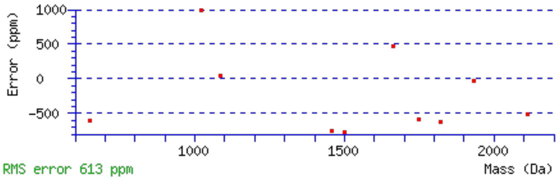

Protein View: LISC_RICCO

Lipoyl synthase, chloroplastic OS=Ricinus communis GN=LIP1P PE=3 SV=1

**Database:**	SwissProt
**Score:**	63
**Expect:**	0.018
**Nominal mass (M_r):**	40734
**Calculated pI:**	7.59
**Taxonomy:**	Ricinus communis

Sequence similarity is available as **an NCBI BLAST search of LISC_RICCO against nr**.

Search parameters

**Enzyme:**	Trypsin: cuts C-term side of KR unless next residue is P.
**Fixed modifications:**	Carbamidomethyl (C)
**Variable modifications:**	Oxidation (M)
**Mass values searched:**	5
**Mass values matched:**	5

Protein sequence coverage: 25%

Matched peptides shown in ***bold red***.

```
  1 MEQTLFNPSI SMPKSFYHKH ITISSRIQCQ LSTNSPSSNT KTTTVTVPSK
 51 KTMGPYTGRD PNVKKPEWLR QRAPQGERFQ EVKHSLSSLK LNTVCEEAQC
101 PNIGECWNGG GDGIATATIM LLGDTCTRGC RFCAVKTSRN PSPPDPLEPQ
151 NTALAIASWG VDYIVLTSVD RDDLPDGGSG HFSETVQAMK KLKPEIMVEC
201 LTSDFRGDLE AVETLVHSGL DVFAHNIETV KRLQRIVRDP RAGYEQSLSV
251 LKHAKHSKEG MITKSSIMLG LGETDDELKE AMADLRAIDV DILTLGQYLQ
301 PTPLHLTVKE YVTPEKFAFW KEYGESIGFR YVASGPMVRS SYRAGELFVK
351 TMVKERSSNS AAKP
```

Unformatted sequence string: **364 residues** (for pasting into other applications).

Sort peptides by ◉ Residue Number ○ Increasing Mass ○ Decreasing Mass

Show predicted peptides also

Start – End	Observed	Mr(expt)	Mr(calc)	Delta	M	Peptide
2 – 19	2170.0882	2169.0809	2169.0565	0.0245	1	M.EQTLFNPSISMPKSFYHK.H + Oxidation (M)
42 – 50	933.4996	932.4923	932.5179	-0.0256	0	K.TTTVTVPSK.K
60 – 70	1381.5494	1380.5421	1380.7514	-0.2092	1	R.DPNVKKPEWLR.Q
207 – 231	2693.2559	2692.2487	2692.3708	-0.1222	0	R.GDLEAVETLVHSGLDVFAHNIETVK.R
280 – 309	3350.6123	3349.6050	3349.7955	-0.1905	1	K.EAMADLRAIDVDILTLGQYLQPTPLHLTVK.E + Oxidatio

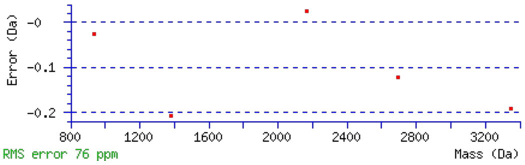

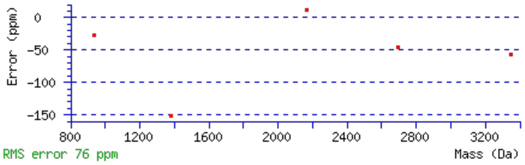

Protein View: ATPB_NICBI

ATP synthase subunit beta, chloroplastic OS=Nicotiana bigelovii GN=atpB PE=3 SV=1

**Database:**	SwissProt
**Score:**	87
**Expect:**	6.9e-005
**Nominal mass (M_r):**	53549
**Calculated pI:**	5.09
**Taxonomy:**	Nicotiana quadrivalvis var. bigelovii

Sequence similarity is available as an NCBI BLAST search of ATPB_NICBI against nr.

Search parameters

**Enzyme:**	Trypsin: cuts C-term side of KR unless next residue is P.
**Fixed modifications:**	Carbamidomethyl (C)
**Variable modifications:**	Oxidation (M)
**Mass values searched:**	19
**Mass values matched:**	11

Protein sequence coverage: 37%

Matched peptides shown in ***bold red***.

1 MR**INPTTSGS GVSTLEK**KNP GRVVQIIGPV LDVAFPPGKM PNIYNALVVQ
51 GR**DSVGQPIN VACEVQQLLG NNR**VRAVAMS ATDGLTR**GME VIDTGAPISV**
101 **PVGGATLGRI FNVLGEPVDN LGPVDTSTTS PIHR**SAPAFI QLDTKLSIFE
151 TGIKVVDLLA PYRRGGKIGL FGGAGVGKTV LIMELINNIA K**AHGGVSVFG**
201 **GVGER**TREGN DLYMEMKESG VINEENIAES K**VALVYGQMN EPPGAR**MR**VG**
251 **LTALTMAEYF RDVNEQDVLL FIDNIFRFVQ AGSEVSALLG R**MPSAVGYQP
301 TLSTEMGSLQ ERITSTKEGS ITSIQAVYVP ADDLTDPAPA TTFAHLDATT
351 VLSRGLAAKG IYPAVDPLDS TSTMLQPR**IV GEEHYETAQR** VKQTLQR**YKE**
401 **LQDIIAILGL DELSEEDR**LL VARARKIERF LSQPFFVAEV FTGSPGKYVG
451 LAETIRGFQL ILSGELDGLP EQAFYLVGNI DEATAKAMNL EMESNLKK

Unformatted sequence string: 498 residues (for pasting into other applications).

Sort peptides by ◉ Residue Number ○ Increasing Mass ○ Decreasing Mass

Show predicted peptides also

Start - End	Observed	Mr(expt)	Mr(calc)	Delta	M	Peptide
3 - 17	1490.6787	1489.6714	1489.7624	-0.0910	0	R.INPTTSGSGVSTLEK.K
53 - 73	2310.9892	2309.9820	2310.1386	-0.1567	0	R.DSVGQPINVACEVQQLLGNNR.V
88 - 109	2112.9519	2111.9446	2112.0885	-0.1439	0	R.GMEVIDTGAPISVPVGGATLGR.I + Oxidation (M)
110 - 134	2678.2050	2677.1977	2677.3712	-0.1735	0	R.IFNVLGEPVDNLGPVDTSTTSPIHR.S
192 - 205	1328.5743	1327.5670	1327.6633	-0.0963	0	K.AHGGVSVFGGVGER.T
232 - 246	1617.6932	1616.6859	1616.7981	-0.1121	0	K.VALVYGQMNEPPGAR.M + Oxidation (M)
249 - 261	1487.6541	1486.6468	1486.7490	-0.1022	0	R.VGLTALTMAEYFR.D + Oxidation (M)
262 - 277	1949.8638	1948.8566	1948.9894	-0.1329	0	R.DVNEQDVLLFIDNIFR.F
278 - 291	1433.6746	1432.6673	1432.7674	-0.1001	0	R.FVQAGSEVSALLGR.M
379 - 390	1431.5868	1430.5795	1430.6790	-0.0995	0	R.IVGEEHYETAQR.V
398 - 418	2462.1044	2461.0972	2461.2587	-0.1616	1	R.YKELQDIIAILGLDELSEEDR.L

No match to: 1390.5866, 1399.6678, 1864.8168, 2185.9971, 2239.0369, 2269.0675, 2329.9661, 2988.4009

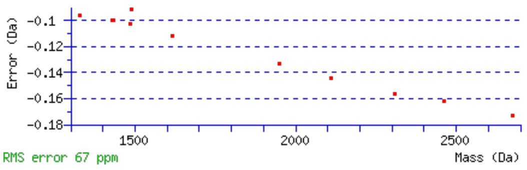

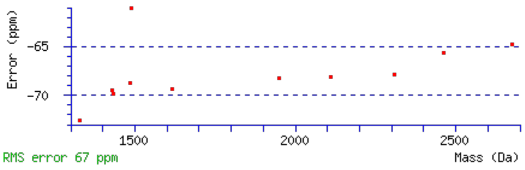

MASCOT Search Results

Protein View: ATPE_ATRBE

ATP synthase epsilon chain, chloroplastic OS=Atropa belladonna GN=atpE PE=3 SV=1

**Database:**	SwissProt
**Score:**	82
**Expect:**	0.00023
**Monoisotopic mass (M_r):**	14588
**Calculated pI:**	5.18
**Taxonomy:**	Atropa belladonna

Sequence similarity is available as an NCBI BLAST search of ATPE_ATRBE against nr.

Search parameters

**Enzyme:**	Trypsin: cuts C-term side of KR unless next residue is P.
**Fixed modifications:**	Carbamidomethyl (C)
**Variable modifications:**	Oxidation (M)
**Mass values searched:**	5
**Mass values matched:**	5

Protein sequence coverage: 45%

Matched peptides shown in ***bold red***.

1 MTLNLSVLTP NRIVWDSEVE EIVLSTNSGQ IGILSNHAPI ATAVDIGILR

51 IR**LNDQWLTM ALMGGFARIG NNEITVLVND AEKGSDIDPQ EAQQTLEIAE**

101 **ANVKK**AEGRR QK**IEANLALR** RARTRVEAIN PIS

Unformatted sequence string: 133 residues (for pasting into other applications).

Sort by ◉ residue number ○ increasing mass ○ decreasing mass
Show ◉ matched peptides only ○ predicted peptides also

Start – End	Observed	Mr(expt)	Mr(calc)	Delta	M	Peptide
53 – 68	1855.8178	1854.8105	1854.8757	-0.0652	0	R.LNDQWLTMALMGGFAR.I + 2 Oxidation (M)
69 – 83	1629.7796	1628.7723	1627.8417	0.9306	0	R.IGNNEITVLVNDAEK.G
84 – 104	2256.9879	2255.9806	2255.0917	0.8889	0	K.GSDIDPQEAQQTLEIAEANVK.K
84 – 105	2384.8405	2383.8333	2383.1867	0.6466	1	K.GSDIDPQEAQQTLEIAEANVKK.A
113 – 120	899.4946	898.4873	898.5236	-0.0363	0	K.IEANLALR.R

Protein View: STAD2_ARATH

Stearoyl-[acyl-carrier-protein] 9-desaturase 2, chloroplastic OS=Arabidopsis thaliana GN=S-ACP-DES2 PE=1 SV=1

**Database:**	SwissProt
**Score:**	84
**Expect:**	0.00015
**Monoisotopic mass (M_r):**	47149
**Calculated pI:**	5.87
**Taxonomy:**	Arabidopsis thaliana

Sequence similarity is available as an NCBI BLAST search of STAD2_ARATH against nr.

Search parameters

**Enzyme:**	Trypsin: cuts C-term side of KR unless next residue is P.
**Fixed modifications:**	Carbamidomethyl (C)
**Variable modifications:**	Oxidation (M)
**Mass values searched:**	8
**Mass values matched:**	8

Protein sequence coverage: 25%

Matched peptides shown in ***bold red***.

```
  1 MALLLNSTIT VAMKQNPLVA VSFPRTTCLG SSFSPPRLLR VSCVATNPSK
 51 TSEETDKKKF RPIKEVPNQV THTITQEKLE IFKSMENWAQ ENLLSYLKPV
101 EASWQPQDFL PETNDEDRFY EQVKELRDRT KEIPDDYFVV LVGDMITEEA
151 LPTYQTTLNT LDGVKDETGG SLTPWAVWVR AWTAEENRHG DLLNKYLYLS
201 GRVDMRHVEK TIQYLIGSGM DSKFENNPYN GFIYTSFQER ATFISHGNTA
251 KLATTYGDTT LAKICGTIAA DEKRHETAYT RIVEKLFEID PDGTVQALAS
301 MMRKRITMPA HLMHDGRDDD LFDHYAAVAQ RIGVYTATDY AGILEFLLRR
351 WEVEKLGMGL SGEGRRAQDY LCTLPQRIRR LEERANDRVK LASKSKPSVS
401 FSWIYGREVE L
```

Unformatted sequence string: 411 residues (for pasting into other applications).

Sort by ◉ residue number ○ increasing mass ○ decreasing mass
Show ◉ matched peptides only ○ predicted peptides also

Start - End	Observed	Mr(expt)	Mr(calc)	Delta	M	Peptide
2 - 25	2600.2805	2599.2732	2598.4567	0.8165	1	M.ALLLNSTITVAMKQNPLVAVSFPR.T + Oxidation (M)
26 - 40	1690.7341	1689.7269	1690.8825	-1.1556	1	R.TTCLGSSFSPPRLLR.V
79 - 83	650.0027	648.9954	648.3846	0.6108	0	K.LEIFK.S
125 - 129	687.9616	686.9543	687.3664	-0.4121	1	K.ELRDR.T
275 - 281	876.9872	875.9800	876.4090	-0.4290	0	R.HETAYTR.I
286 - 303	1994.8447	1993.8374	1992.9649	0.8725	0	K.LFEIDPDGTVQALASMMR.K
351 - 365	1663.7252	1662.7180	1662.8035	-0.0856	1	R.WEVEKLGMGLSGEGR.R + Oxidation (M)
391 - 407	1913.9026	1912.8953	1912.0207	0.8747	1	K.LASKSKPSVSFSWIYGR.E

MATRIX SCIENCE MASCOT Search Results

Protein View: KCS14_ARATH

Probable 3-ketoacyl-CoA synthase 14 OS=Arabidopsis thaliana GN=KCS14 PE=2 SV=1

**Database:**	SwissProt
**Score:**	66
**Expect:**	0.0095
**Monoisotopic mass (M_r):**	52163
**Calculated pI:**	9.18
**Taxonomy:**	Arabidopsis thaliana

Sequence similarity is available as an NCBI BLAST search of KCS14_ARATH against nr.

Search parameters

**Enzyme:**	Trypsin: cuts C-term side of KR unless next residue is P.
**Fixed modifications:**	Carbamidomethyl (C)
**Variable modifications:**	Oxidation (M)
**Mass values searched:**	6
**Mass values matched:**	6

Protein sequence coverage: 19%

Matched peptides shown in ***bold red***.

```
  1 MFIAMADFKL LLLILILLSL FELDLLHFHH DFFSPFPVKI GLLLISIFFY
 51 AYSTTRSKPV YLVDFSCHQP TDSCKISSET FFNMAKGAQL YTEETIQFMT
101 RILNRSGLGD DTYSPRCMLT SPPTPSMYEA RHESELVIFG ALNSLFKKTG
151 IEPREVGIFI VNCSLFNPNP SLSSMIVNRY KLKTDVKTYN LSGISVDLAT
201 NLLKANPNTY AVIVSTENMT LSMYRGNDRS MLVPNCLFRV GGAAVMLSNR
251 SQDRVRSKYE LTHIVRTHKG SSDKHYTCAE QKEDSKGIVG VALSKELTVV
301 AGDTLKTNLT ALGPLVLPLS EKLRFILFLV KSKLFRLKVS PYVPDFKLCF
351 KHFCIHAGGR ALLDAVEKGL GLSEFDLEPS RMTLHRFGNT SSSSLWYELA
401 YVEAKCRVKR GDRVWQLAFG SGFKCNSIVW RALRTIPANE SLVGNPWGDS
451 VHKYPVHVT
```

Unformatted sequence string: 459 residues (for pasting into other applications).

Sort by ◉ residue number ○ increasing mass ○ decreasing mass
Show ◉ matched peptides only ○ predicted peptides also

Start - End	Observed	Mr(expt)	Mr(calc)	Delta	M	Peptide
87 - 101	1786.7644	1785.7571	1786.8560	-1.0989	0	K.GAQLYTEETIQFMTR.I
106 - 131	2922.1500	2921.1427	2920.2677	0.8750	1	R.SGLGDDTYSPRCMLTSPPTPSMYEAR.H + 2 Oxidation (M)
149 - 154	671.9854	670.9781	671.3602	-0.3821	0	K.TGIEPR.E
155 - 179	2807.0928	2806.0855	2806.4146	-0.3291	0	R.EVGIFIVNCSLFNPNPSLSSMIVNR.Y
226 - 239	1694.8586	1693.8513	1693.8028	0.0485	1	R.GNDRSMLVPNCLFR.V + Oxidation (M)
332 - 336	649.9925	648.9853	649.3911	-0.4059	1	K.SKLFR.L

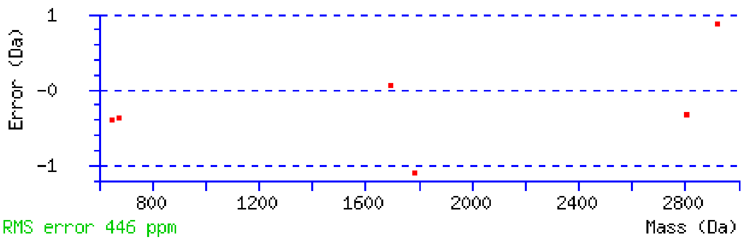

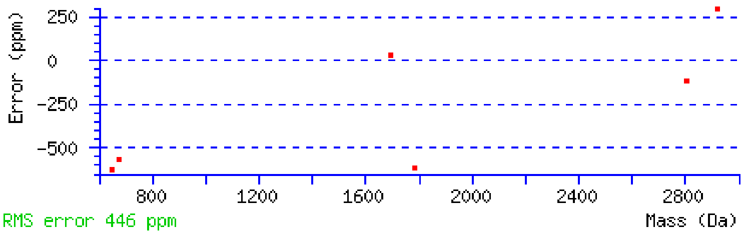

```
ID   KCS14_ARATH             Reviewed;         459 AA.
AC   Q9SS39;
DT   05-SEP-2006, integrated into UniProtKB/Swiss-Prot.
DT   01-MAY-2000, sequence version 1.
DT   15-FEB-2017, entry version 102.
DE   RecName: Full=Probable 3-ketoacyl-CoA synthase 14 {ECO:0000303|PubMed:18465198};
DE            Short=KCS-14 {ECO:0000303|PubMed:18465198};
DE            EC=2.3.1.199 {ECO:0000305};
DE   AltName: Full=Very long-chain fatty acid condensing enzyme 14 {ECO:0000303|PubMed:18465198};
DE            Short=VLCFA condensing enzyme 14 {ECO:0000303|PubMed:18465198};
DE   Flags: Precursor;
GN   Name=KCS14 {ECO:0000303|PubMed:18465198};
GN   OrderedLocusNames=At3g10280 {ECO:0000312|Araport:AT3G10280};
GN   ORFNames=F14P13.12 {ECO:0000312|EMBL:AAF02814.1};
OS   Arabidopsis thaliana (Mouse-ear cress).
OC   Eukaryota; Viridiplantae; Streptophyta; Embryophyta; Tracheophyta;
OC   Spermatophyta; Magnoliophyta; eudicotyledons; Gunneridae;
OC   Pentapetalae; rosids; malvids; Brassicales; Brassicaceae; Camelineae;
OC   Arabidopsis.
OX   NCBI_TaxID=3702;
RN   [1]
RP   NUCLEOTIDE SEQUENCE [LARGE SCALE GENOMIC DNA].
RC   STRAIN=cv. Columbia;
RX   PubMed=11130713; DOI=10.1038/35048706;
RA   Salanoubat M., Lemcke K., Rieger M., Ansorge W., Unseld M.,
RA   Fartmann B., Valle G., Bloecker H., Perez-Alonso M., Obermaier B.,
RA   Delseny M., Boutry M., Grivell L.A., Mache R., Puigdomenech P.,
RA   De Simone V., Choisne N., Artiguenave F., Robert C., Brottier P.,
RA   Wincker P., Cattolico L., Weissenbach J., Saurin W., Quetier F.,
RA   Schaefer M., Mueller-Auer S., Gabel C., Fuchs M., Benes V.,
RA   Wurmbach E., Drzonek H., Erfle H., Jordan N., Bangert S.,
RA   Wiedelmann R., Kranz H., Voss H., Holland R., Brandt P., Nyakatura G.,
RA   Vezzi A., D'Angelo M., Pallavicini A., Toppo S., Simionati B.,
RA   Conrad A., Hornischer K., Kauer G., Loehnert T.-H., Nordsiek G.,
RA   Reichelt J., Scharfe M., Schoen O., Bargues M., Terol J., Climent J.,
RA   Navarro P., Collado C., Perez-Perez A., Ottenwaelder B., Duchemin D.,
RA   Cooke R., Laudie M., Berger-Llauro C., Purnelle B., Masuy D.,
RA   de Haan M., Maarse A.C., Alcaraz J.-P., Cottet A., Casacuberta E.,
RA   Monfort A., Argiriou A., Flores M., Liguori R., Vitale D.,
RA   Mannhaupt G., Haase D., Schoof H., Rudd S., Zaccaria P., Mewes H.-W.,
RA   Mayer K.F.X., Kaul S., Town C.D., Koo H.L., Tallon L.J., Jenkins J.,
RA   Rooney T., Rizzo M., Walts A., Utterback T., Fujii C.Y., Shea T.P.,
RA   Creasy T.H., Haas B., Maiti R., Wu D., Peterson J., Van Aken S.,
RA   Pai G., Militscher J., Sellers P., Gill J.E., Feldblyum T.V.,
RA   Preuss D., Lin X., Nierman W.C., Salzberg S.L., White O., Venter J.C.,
RA   Fraser C.M., Kaneko T., Nakamura Y., Sato S., Kato T., Asamizu E.,
RA   Sasamoto S., Kimura T., Idesawa K., Kawashima K., Kishida Y.,
RA   Kiyokawa C., Kohara M., Matsumoto M., Matsuno A., Muraki A.,
RA   Nakayama S., Nakazaki N., Shinpo S., Takeuchi C., Wada T.,
RA   Watanabe A., Yamada M., Yasuda M., Tabata S.;
RT   "Sequence and analysis of chromosome 3 of the plant Arabidopsis
RT   thaliana.";
RL   Nature 408:820-822(2000).
RN   [2]
RP   GENOME REANNOTATION.
RC   STRAIN=cv. Columbia;
RG   The Arabidopsis Information Resource (TAIR);
RL   Submitted (APR-2011) to the EMBL/GenBank/DDBJ databases.
```

```
RN   [3]
RP   INDUCTION, AND GENE FAMILY.
RX   PubMed=12916765; DOI=10.1002/ps.714;
RA   Lechelt-Kunze C., Meissner R.C., Drewes M., Tietjen K.;
RT   "Flufenacet herbicide treatment phenocopies the fiddlehead mutant in
RT   Arabidopsis thaliana.";
RL   Pest Manag. Sci. 59:847-856(2003).
RN   [4]
RP   GENE FAMILY, NOMENCLATURE, 3D-STRUCTURE MODELING, AND TISSUE
RP   SPECIFICITY.
RX   PubMed=18465198; DOI=10.1007/s11103-008-9339-z;
RA   Joubes J., Raffaele S., Bourdenx B., Garcia C., Laroche-Traineau J.,
RA   Moreau P., Domergue F., Lessire R.;
RT   "The VLCFA elongase gene family in Arabidopsis thaliana: phylogenetic
RT   analysis, 3D modelling and expression profiling.";
RL   Plant Mol. Biol. 67:547-566(2008).
CC   -!- CATALYTIC ACTIVITY: A very-long-chain acyl-CoA + malonyl-CoA = CoA
CC       + a very-long-chain 3-oxoacyl-CoA + CO(2). {ECO:0000305}.
CC   -!- PATHWAY: Lipid metabolism; fatty acid biosynthesis.
CC   -!- SUBCELLULAR LOCATION: Membrane {ECO:0000255}; Single-pass membrane
CC       protein {ECO:0000255}.
CC   -!- TISSUE SPECIFICITY: Expressed in siliques.
CC       {ECO:0000269|PubMed:18465198}.
CC   -!- INDUCTION: Repressed by herbicides such as flufenacet and
CC       benfuresate. {ECO:0000269|PubMed:12916765}.
CC   -!- SIMILARITY: Belongs to the chalcone/stilbene synthases family.
CC       {ECO:0000305}.
DR   EMBL; AC009400; AAF02814.1; -; Genomic_DNA.
DR   EMBL; CP002686; AEE74883.1; -; Genomic_DNA.
DR   RefSeq; NP_187639.1; NM_111863.2.
DR   UniGene; At.36468; -.
DR   UniGene; At.50165; -.
DR   ProteinModelPortal; Q9SS39; -.
DR   STRING; 3702.AT3G10280.1; -.
DR   PaxDb; Q9SS39; -.
DR   EnsemblPlants; AT3G10280.1; AT3G10280.1; AT3G10280.
DR   GeneID; 820190; -.
DR   Gramene; AT3G10280.1; AT3G10280.1; AT3G10280.
DR   KEGG; ath:AT3G10280; -.
DR   Araport; AT3G10280; -.
DR   TAIR; locus:2076254; AT3G10280.
DR   eggNOG; ENOG410JWTH; Eukaryota.
DR   eggNOG; COG3424; LUCA.
DR   HOGENOM; HOG000238893; -.
DR   InParanoid; Q9SS39; -.
DR   KO; K15397; -.
DR   OrthoDB; EOG093606UZ; -.
DR   PhylomeDB; Q9SS39; -.
DR   UniPathway; UPA00094; -.
DR   PRO; PR:Q9SS39; -.
DR   Proteomes; UP000006548; Chromosome 3.
DR   Genevisible; Q9SS39; AT.
DR   GO; GO:0016021; C:integral component of membrane; IEA:UniProtKB-KW.
DR   GO; GO:0102336; F:3-oxo-arachidoyl-CoA synthase activity; IEA:UniProtKB-EC.
DR   GO; GO:0102337; F:3-oxo-cerotoyl-CoA synthase activity; IEA:UniProtKB-EC.
DR   GO; GO:0102338; F:3-oxo-lignoceronyl-CoA synthase activity; IEA:UniProtKB-EC.
DR   GO; GO:0006633; P:fatty acid biosynthetic process; IEA:UniProtKB-UniPathway.
DR   Gene3D; 3.40.47.10; -; 2.
DR   InterPro; IPR012392; 3-ktacl-CoA_syn.
DR   InterPro; IPR013747; ACP_syn_III_C.
DR   InterPro; IPR013601; FAE1_typ3_polyketide_synth.
DR   InterPro; IPR016039; Thiolase-like.
DR   PANTHER; PTHR31561; PTHR31561; 1.
DR   Pfam; PF08541; ACP_syn_III_C; 1.
DR   Pfam; PF08392; FAE1_CUT1_RppA; 1.
DR   PIRSF; PIRSF036417; 3-ktacl-CoA_syn; 1.
DR   SUPFAM; SSF53901; SSF53901; 2.
PE   2: Evidence at transcript level;
KW   Acyltransferase; Complete proteome; Membrane; Reference proteome;
KW   Signal; Transferase; Transmembrane; Transmembrane helix.
FT   SIGNAL        1     25       {ECO:0000255}.
FT   CHAIN        26    459       Probable 3-ketoacyl-CoA synthase 14.
FT                                /FTId=PRO_0000249106.
FT   TRANSMEM     32     52       Helical. {ECO:0000255}.
FT   DOMAIN       52    334       FAE. {ECO:0000255}.
FT   ACT_SITE    268    268       {ECO:0000250|UniProtKB:Q38860}.
FT   ACT_SITE    352    352       {ECO:0000250|UniProtKB:Q38860}.
FT   ACT_SITE    356    356       {ECO:0000250|UniProtKB:Q38860}.
FT   ACT_SITE    385    385       {ECO:0000250|UniProtKB:Q38860}.
FT   ACT_SITE    389    389       {ECO:0000250|UniProtKB:Q38860}.
SQ   SEQUENCE   459 AA;  51626 MW;  1307C2E49C34C509 CRC64;
     MFIAMADFKL LLLILILLSL FELDLLHFHH DFFSPFPVKI GLLLISIFFY AYSTTRSKPV
```

```
YLVDFSCHQP TDSCKISSET FFNMAKGAQL YTEETIQFMT RILNRSGLGD DTYSPRCMLT
SPPTPSMYEA RHESELVIFG ALNSLFKKTG IEPREVGIFI VNCSLFNPNP SLSSMIVNRY
KLKTDVKTYN LSGISVDLAT NLLKANPNTY AVIVSTENMT LSMYRGNDRS MLVPNCLFRV
GGAAVMLSNR SQDRVRSKYE LTHIVRTHKG SSDKHYTCAE QKEDSKGIVG VALSKELTVV
AGDTLKTNLT ALGPLVLPLS EKLRFILFLV KSKLFRLKVS PYVPDFKLCF KHFCIHAGGR
ALLDAVEKGL GLSEFDLEPS RMTLHRFGNT SSSSLWYELA YVEAKCRVKR GDRVWQLAFG
SGFKCNSIVW RALRTIPANE SLVGNPWGDS VHKYPVHVT
```

Protein View: K6PF4_ARATH

6-phosphofructokinase 4, chloroplastic OS=Arabidopsis thaliana GN=PFK4 PE=1 SV=1

**Database:**	SwissProt
**Score:**	70
**Expect:**	0.0038
**Nominal mass (M_r):**	58886
**Calculated pI:**	8.46
**Taxonomy:**	Arabidopsis thaliana

Sequence similarity is available as an NCBI BLAST search of K6PF4_ARATH against nr.

Search parameters

**Enzyme:**	Trypsin: cuts C-term side of KR unless next residue is P.
**Fixed modifications:**	Carbamidomethyl (C)
**Variable modifications:**	Oxidation (M)
**Mass values searched:**	6
**Mass values matched:**	6

Protein sequence coverage: 22%

Matched peptides shown in ***bold red***.

1 **MEASISFLGS TKPNISLFNP SSNVLPR**RDF PLPALKLKKV SVLPRILHQK
51 RLIRAQCSDG FKPEEDDGFV LEDVPHLTK**F LPDLPSYPNP LK**ESQAYAIV
101 KRTFVSSEDV VAQNIVVQKG SKRGVHFRRA GPRERVYFRS DEVKACIVTC
151 GGLCPGINTV IREIVCGLNN MYGVNNILGI QGGYRGFYSK NTMNLTPKVV
201 NDIHKRGGTF LQTSRGGHDT AKIVDNIQDR GINQVYIIGG GGTQKGAEKI
251 YEEVERRGLQ VAVSGIPKTI DNDIAVIDKS FGFDTAVEEA QRAINAAHVE
301 VESVENGVGI VKLMGRYSGF IAMIATLANR DVDCCLIPES PFFLEGKGGL
351 FEFIEERLKE NR**HMVIVIAE GAGQDYVAQS MRASETKDAS GNRLLLDVGL**
401 **WLTQQIK**DHF TNVRK**MMINM KYIDPTYMIR** AIPSNASDNV YCTLLAQSAV
451 HGAMAGYSGF TVGPVNSR**HA YIPISQVTEV TNTVKLTDR**M WARLLASTNQ
501 PSFLTGEGAL QNVIDMETQE KIDNMKISSI

Unformatted sequence string: 530 residues (for pasting into other applications).

Sort peptides by ◉ Residue Number ○ Increasing Mass ○ Decreasing Mass

Show predicted peptides also

Start – End	Observed	Mr(expt)	Mr(calc)	Delta	M	Peptide
1 – 27	2921.4328	2920.4255	2921.4957	-1.0701	0	-.MEASISFLGSTKPNISLFNPSSNVLPR.R + Oxidation ([illegible]
80 – 92	1501.7287	1500.7214	1499.8024	0.9190	0	K.FLPDLPSYPNPLK.E
363 – 387	2691.3465	2690.3392	2690.3156	0.0236	1	R.HMVIVIAEGAGQDYVAQSMRASETK.D
388 – 407	2239.2031	2238.1958	2239.2325	-1.0367	1	K.DASGNRLLLDVGLWLTQQIK.D
416 – 430	1952.7327	1951.7255	1950.9075	0.8179	1	K.MMINMKYIDPTYMIR.A + 2 Oxidation (M)
469 – 489	2385.0147	2384.0074	2384.2700	-0.2626	1	R.HAYIPISQVTEVTNTVKLTDR.M

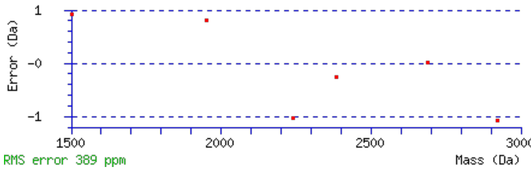

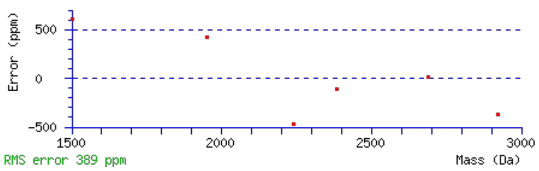

Actin-54 (Fragment) OS=Nicotiana tabacum PE=3 SV=1

**Database:**	SwissProt
**Score:**	59
**Expect:**	0.05
**Nominal mass (M_r):**	37521
**Calculated pI:**	5.66
**Taxonomy:**	Nicotiana tabacum

Sequence similarity is available as an NCBI BLAST search of ACT3_TOBAC against nr.

Search parameters

**Enzyme:**	Trypsin: cuts C-term side of KR unless next residue is P.
**Fixed modifications:**	Carbamidomethyl (C)
**Variable modifications:**	Oxidation (M)
**Mass values searched:**	4
**Mass values matched:**	4

Protein sequence coverage: 15%

Matched peptides shown in ***bold red***.

```
  1 AGFAGDDAPR AVFPSIVGRP RHTGVMVGMG QKDAYVGDEA QSKRGILTLK
 51 YPIEHGIASN WDDMEKIWHH TFYNELRVAP EEHPVLLTEA PLNPKANREK
101 MTQIMFETFS VPAMYVAIQA VLSLYASGRT TGTGIVLDSG DGVSHHVPIY
151 EGYALPHAIL RLDLAGRDLT DSLMKILTER GYMFTTTAER EIVRDMKEKL
201 AYVALDYEQE LETARSSSSI EKNYELPDGQ VITIGAERFR CPEVLFQPSM
251 IGMEAAGIHE TTYNSIMKRD VDIRKDLYGN IVLSGGSTMF PGIADRMSKE
301 ITALAPSSMK IKVVAPPERK YSVWIGGSIL ASLSTLQQV
```

Unformatted sequence string: 339 residues (for pasting into other applications).

Sort peptides by ◉ Residue Number ○ Increasing Mass ○ Decreasing Mass

Show predicted peptides also

Start – End	Observed	Mr(expt)	Mr(calc)	Delta	M	Peptide
67 – 77	1515.6662	1514.6589	1514.7419	-0.0829	0	K.IWHHTFYNELR.V
162 – 175	1547.6545	1546.6473	1546.8025	-0.1552	1	R.LDLAGRDLTDSLMK.I
181 – 190	1192.4647	1191.4574	1191.5230	-0.0656	0	R.GYMFTTTAER.E + Oxidation (M)
223 – 238	1774.8044	1773.7971	1773.8897	-0.0926	0	K.NYELPDGQVITIGAER.F

Protein View: EXB13_ORYSJ

Expansin-B13 OS=Oryza sativa subsp. japonica GN=EXPB13 PE=3 SV=2

**Database:**	SwissProt
**Score:**	83
**Expect:**	0.00016
**Nominal mass (M_r):**	24653
**Calculated pI:**	5.55
**Taxonomy:**	Oryza sativa Japonica Group

Sequence similarity is available as **an NCBI BLAST search of EXB13_ORYSJ against nr**.

Search parameters

**Enzyme:**	Trypsin: cuts C-term side of KR unless next residue is P.
**Fixed modifications:**	Carbamidomethyl (C)
**Variable modifications:**	Oxidation (M)
**Mass values searched:**	6
**Mass values matched:**	6

Protein sequence coverage: 39%

Matched peptides shown in ***bold red***.

```
  1 MASSSLLLAS VVVAAMVSAV SCGPPKVPPG PNITASYGDK WLEARATWYG
 51 AAKGAGRKDN SGACGYKDVD KAPFLGMNSC GNDPIFKDGK GCGSCFEIKC
101 SKPKACSDKP VLIHVTDMND EPIAAYHFDL FGLAFGAMAK DGKDEELLKY
151 VAGDGDVVEV EIKEKGSEEW KALKESWGAI WRIDTPKPLK GPFSVRVTTE
201 GGEKIIAEDA IPDGWKADSV YKSNVQAK
```

Unformatted sequence string: **228 residues** (for pasting into other applications).

Sort peptides by ◉ Residue Number ○ Increasing Mass ○ Decreasing Mass

Show predicted peptides also

Start – End	Observed	Mr(expt)	Mr(calc)	Delta	M	Peptide
46 – 57	1207.5688	1206.5615	1207.6098	-1.0483	1	R.ATWYGAAKGAGR.K
59 – 71	1429.7513	1428.7441	1427.5987	1.1454	1	K.DNSGACGYKDVDK.A
72 – 87	1784.8570	1783.8497	1782.8069	1.0428	0	K.APFLGMNSCGNDPIFK.D + Oxidation (M)
144 – 163	2221.0446	2220.0374	2219.1209	0.9165	1	K.DEELLKYVAGDGDVVEVEIK.E
175 – 190	1897.9271	1896.9198	1896.0257	0.8941	1	K.ESWGAIWRIDTPKPLK.G
191 – 204	1462.7648	1461.7575	1462.7416	-0.9841	1	K.GPFSVRVTTEGGEK.I

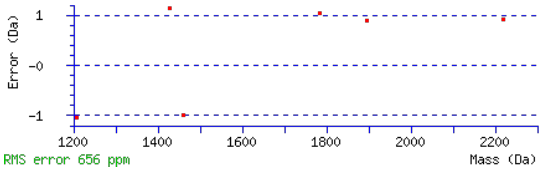

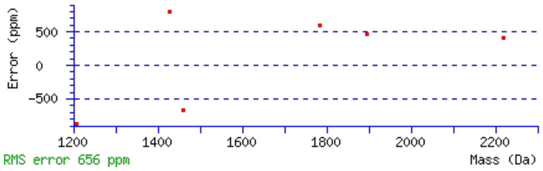

Protein View: VPS36_ARATH

Vacuolar protein sorting-associated protein 36 OS=Arabidopsis thaliana GN=VPS36 PE=1 SV=1

**Database:**	SwissProt
**Score:**	69
**Expect:**	0.0047
**Nominal mass (M_r):**	49231
**Calculated pI:**	5.54
**Taxonomy:**	Arabidopsis thaliana

Sequence similarity is available as an NCBI BLAST search of VPS36_ARATH against nr.

Search parameters

**Enzyme:**	Trypsin: cuts C-term side of KR unless next residue is P.
**Fixed modifications:**	Carbamidomethyl (C)
**Variable modifications:**	Oxidation (M)
**Mass values searched:**	6
**Mass values matched:**	6

Protein sequence coverage: 27%

Matched peptides shown in ***bold red***.

```
  1 MASGSSSIAI GGLFENAEVT TSGRPVLRRN EVECFLLSSI DIDSEDDPPR
 51 FTALRSGNLI LTTHRLIWIP SQSNESVPSS IPLSAVTHIY SHKKSIKSMF
101 HSPRIRFQAD PGSIVVTIVF RGKGDFDGFL SKLWECWRGR AWEEEEKSES
151 ETSKSGSGTV AQGLYGNDGT VRMVGLAGIL RKEQEQWEST DKSLQDAFQD
201 LNALMSKAKE MVSLAEKMRQ KLLSAPSSQN GSTDDEEMGS KEEMQQWMLS
251 VGIISPVTKE SAGALYHQEL SRQLADFVRI PLEKAGGMIS LTDMYYHFNR
301 ARGTELISPD DLWQACTLWE KFDVPVMLRK FDSGVMVIQN KSHSDEEVMS
351 RIRMLVTKTE TLRVGVTASD AALTLKIAPA MAKEHLLSAE TKGLLCRDMS
401 PDGLRFYFNL FPEIDPTNLH IVKEFGTYGE WIKATSLLSV
```

Unformatted sequence string: 440 residues (for pasting into other applications).

Sort peptides by ◉ Residue Number ○ Increasing Mass ○ Decreasing Mass

Show predicted peptides also

Start - End	Observed	Mr(expt)	Mr(calc)	Delta	M	Peptide
1 - 28	2807.1753	2806.1680	2806.4283	-0.2603	0	-.MASGSSSIAIGGLFENAEVTTSGRPVLR.R
51 - 65	1700.7863	1699.7790	1698.9529	0.8261	1	R.FTALRSGNLILTTHR.L
148 - 172	2487.0437	2486.0365	2486.1521	-0.1157	1	K.SESETSKSGSGTVAQGLYGNDGTVR.M
280 - 300	2472.0419	2471.0346	2471.1977	-0.1631	1	R.IPLEKAGGMISLTDMYYHFNR.A + Oxidation (M)
342 - 353	1462.7361	1461.7289	1460.6678	1.0611	1	K.SHSDEEVMSRIR.M + Oxidation (M)
406 - 423	2206.9699	2205.9626	2206.1463	-0.1837	0	R.FYFNLFPEIDPTNLHIVK.E

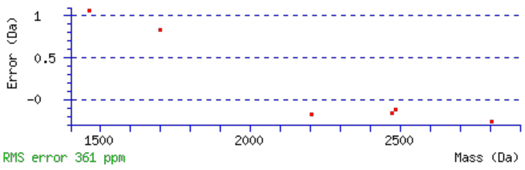

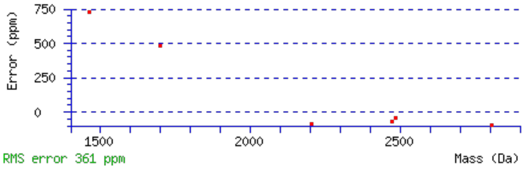

Protein View: KRP1_ARATH

Cyclin-dependent kinase inhibitor 1 OS=Arabidopsis thaliana GN=KRP1 PE=1 SV=2

**Database:**	SwissProt
**Score:**	66
**Expect:**	0.0077
**Nominal mass (M_r):**	22497
**Calculated pI:**	5.29
**Taxonomy:**	Arabidopsis thaliana

Sequence similarity is available as an NCBI BLAST search of KRP1_ARATH against nr.

Search parameters

**Enzyme:**	Trypsin: cuts C-term side of KR unless next residue is P.
**Fixed modifications:**	Carbamidomethyl (C)
**Variable modifications:**	Oxidation (M)
**Mass values searched:**	5
**Mass values matched:**	5

Protein sequence coverage: 36%

Matched peptides shown in ***bold red***.

```
  1 MVRKYRKAKG IVEAGVSSTY MQLRSRRIVY VRSEKSSSVS VVGDNGVSSS
 51 CSGSNEYKKK ELIHLEEEDK DGDTETSTYR RGTKRKLFEN LREEEKEELS
101 KSMENYSSEF ESAVKESLDC CCSGRKTMEE TVTAEEEEKA KLMTEMPTES
151 EIEDFFVEAE KQLKEKFKKK YNFDFEKEKP LEGRYEWVKL E
```

Unformatted sequence string: 191 residues (for pasting into other applications).

Sort peptides by ◉ Residue Number ○ Increasing Mass ○ Decreasing Mass

Show predicted peptides also

Start - End	Observed	Mr(expt)	Mr(calc)	Delta	M	Peptide
10 - 26	1868.7747	1867.7675	1868.9414	-1.1740	1	K.GIVEAGVSSTYMQLRSR.R + Oxidation (M)
28 - 32	649.9947	648.9874	648.3959	0.5915	0	R.IVYVR.S
71 - 81	1301.5511	1300.5439	1299.5691	0.9747	1	K.DGDTETSTYRR.G
102 - 125	2833.1430	2832.1357	2831.1143	1.0215	1	K.SMENYSSEFESAVKESLDCCCSGR.K
127 - 139	1542.6883	1541.6811	1540.6450	1.0361	0	K.TMEETVTAEEEEK.A + Oxidation (M)

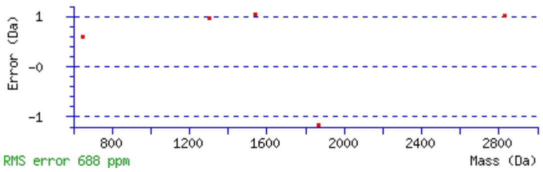

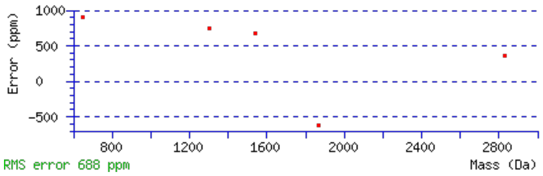

Protein View: CSPL9_MAIZE

CASP-like protein 9 OS=Zea mays PE=2 SV=1

**Database:**	SwissProt
**Score:**	65
**Expect:**	0.01
**Nominal mass (M_r):**	19804
**Calculated pI:**	9.41
**Taxonomy:**	Zea mays

Sequence similarity is available as an NCBI BLAST search of CSPL9_MAIZE against nr.

Search parameters

**Enzyme:**	Trypsin: cuts C-term side of KR unless next residue is P.
**Fixed modifications:**	Carbamidomethyl (C)
**Variable modifications:**	Oxidation (M)
**Mass values searched:**	4
**Mass values matched:**	4

Protein sequence coverage: 38%

Matched peptides shown in ***bold red***.

```
  1 MAAGPPSSVR AERVLRAACA AMAAAGALLL GFSAQTKTVL FIQKKAVPKD
 51 VQALWVLIVA AAAAAAYHVA QLARCFCMER LAITGGGGGC RRLGRAVACA
101 SFLLDKGCAY MVFATTVAAL QACFVGLLGV DALQWSKLCN IYTRFCEQAA
151 AGMVCSLLAA AGMAVLSAFS ARDLFRRPCS PEYR
```

Unformatted sequence string: 184 residues (for pasting into other applications).

Sort peptides by (•) Residue Number ( ) Increasing Mass ( ) Decreasing Mass

Show predicted peptides also

Start - End	Observed	Mr(expt)	Mr(calc)	Delta	M	Peptide
14 - 37	2378.9959	2377.9886	2377.2610	0.7276	1	R.VLRAACAAMAAAGALLLGFSAQTK.T + Oxidation (M)
17 - 37	1994.8252	1993.8179	1993.0125	0.8054	0	R.AACAAMAAAGALLLGFSAQTK.T
93 - 106	1520.6973	1519.6900	1519.8181	-0.1280	1	R.LGRAVACASFLLDK.G
145 - 176	3424.3318	3423.3245	3422.6243	0.7002	1	R.FCEQAAAGMVCSLLAAAGMAVLSAFSARDLFR.R + 2 Oxid

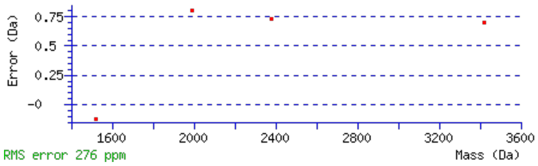

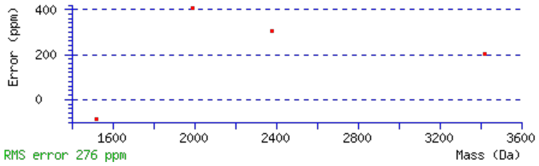

MATRIX
SCIENCE
MASCOT Search Results

Proteln Vlew: PROF3_CORAV

Proflln-3 OS=Corylus avellana PE=1 SV=1

**Database:**	SwlssProt
**Score:**	44
**Expect:**	1.5
**Nomlnal mass (M_r):**	14294
**Calculated pI:**	4.89
**Taxonomy:**	Corylus avellana

Sequence slmllarlty ls avallable as an NCBI BLAST search of PROF3_CORAV agalnst nr.

Search parameters

**MS data flle:**	peaklist.xml
**Enzyme:**	Trypsln: cuts C-term slde of KR unless next resldue ls P.
**Flxed modlflcatlons:**	Carbamldomethyl (C)
**Varlable modlflcatlons:**	Oxldatlon (M)
**Mass values searched:**	18
**Mass values matched:**	4

Proteln sequence coverage: 57%

Matched peptldes shown ln ***bold red***.

```
  1 MSWQAYVDEH LMCDIDGQGQ QLAASAIVGH DGSVWAQSSS FPQLKPEEIT
 51 GIMKDFDEPG HLAPTGLHLG GTKYMVIQGE AGAVIRGKKG SGGITIKKTG
101 QALVFGIYEE PVTPGQCNMV VERLGDYLVE QGL
```

Unformatted sequence strlng: 133 resldues (for pastlng lnto other appllcatlons).

Sort peptldes by ● Resldue Number 0 Increaslng Mass 0 Decreaslng Mass

Show predlcted peptldes also

Start – End	Observed	Mr(expt)	Mr(calc)	Delta	M	Peptide
55 – 86	3349.3948	3348.3875	3348.6925	-0.3050	1	K.DFDEPGHLAPTGLHLGGTKYMVIQGEAGAVIR.G
90 – 98	860.9985	859.9912	859.5127	0.4785	1	K.GSGGITIKK.T
98 – 123	2922.1497	2921.1424	2921.4416	-0.2992	1	K.KTGQALVFGIYEEPVTPGQCNMVVER.L
124 – 133	1106.4408	1105.4335	1105.5655	-0.1320	0	R.LGDYLVEQGL.-

No match to: 1065.9856, 1194.0992, 1234.5907, 1365.5361, 1404.5507, 1602.6366, 1691.7271, 1791.5986, 2239.9558, 2384.7822, 2563.9907, 2692.1018, 2807.1182, 3332.3794

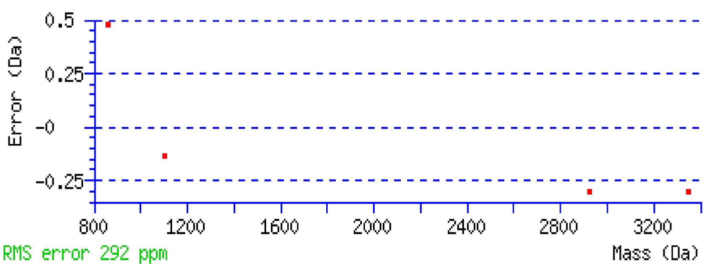

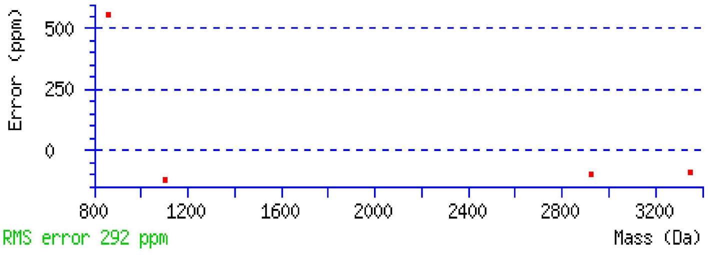

Protein View: PR19A_ARATH

Pre-mRNA-processing factor 19 homolog 1 OS=Arabidopsis thaliana GN=PRP19A PE=1 SV=1

**Database:**	SwissProt
**Score:**	71
**Expect:**	0.0029
**Nominal mass (M_r):**	57230
**Calculated pI:**	6.16
**Taxonomy:**	Arabidopsis thaliana

Sequence similarity is available as an NCBI BLAST search of PR19A_ARATH against nr.

Search parameters

**Enzyme:**	Trypsin: cuts C-term side of KR unless next residue is P.
**Fixed modifications:**	Carbamidomethyl (C)
**Variable modifications:**	Oxidation (M)
**Mass values searched:**	6
**Mass values matched:**	6

Protein sequence coverage: 23%

Matched peptides shown in ***bold red***.

```
  1 MNCAISGEVP EEPVVSKKSG LLYEKRLIQT HISDYGKCPV TGEPHTLDDI
 51 VPIKTGKIVK PKPLHTASIP GLLGTFQTEW DSLMLSNFAL EQQLHTARQE
101 LSHALYQHDA ACRVIARLKK ERDESRQLLA EAERQLPAAP EVATSNAALS
151 NGKRGIDDGE QGPNAKKMRL GISAEVITEL TDCNAALSQQ RKKRQIPKTL
201 ASVDALEKFT QLSSHPLHKT NKPGIFSMDI LHSKDVIATG GIDTTAVLFD
251 RPSGQILSTL TGHSKKVTSI KFVGDTDLVL TASSDKTVRI WGCSEDGNYT
301 SRHTLKDHSA EVRAVTVHAT NKYFVSASLD STWCFYDLSS GLCLAQVTDA
351 SENDVNYTAA AFHPDGLILG TGTAQSIVKI WDVKSQANVA KFGGHNGEIT
401 SISFSENGYF LATAALDGVR LWDLRKLKNF RTFDFPDANS VEFDHSGSYL
451 GIAASDIRVF QAASVKAEWN PIKTLPDLSG TGKATSVKFG LDSKYIAVGS
501 MDRNLRIFGL PDDDNTEDSA QDS
```

Unformatted sequence string: 523 residues (for pasting into other applications).

Sort peptides by ◉ Residue Number ○ Increasing Mass ○ Decreasing Mass

Show predicted peptides also

Start – End	Observed	Mr(expt)	Mr(calc)	Delta	M	Peptide
1 – 17	1862.7694	1861.7621	1860.8597	0.9024	0	-.MNCAISGEVPEEPVVSK.K + Oxidation (M)
99 – 117	2238.9934	2237.9861	2237.1123	0.8738	1	R.QELSHALYQHDAACRVIAR.L
135 – 154	1994.8283	1993.8210	1994.0545	-0.2335	1	R.QLPAAPEVATSNAALSNGKR.G
168 – 191	2692.1019	2691.0947	2691.3320	-0.2373	1	K.MRLGISAEVITELTDCNAALSQQR.K + Oxidation (M)
195 – 208	1513.7159	1512.7087	1511.8559	0.8528	1	R.QIPKTLASVDALEK.F
429 – 458	3349.4069	3348.3997	3347.5483	0.8513	1	K.NFRTFDFPDANSVEFDHSGSYLGIAASDIR.V

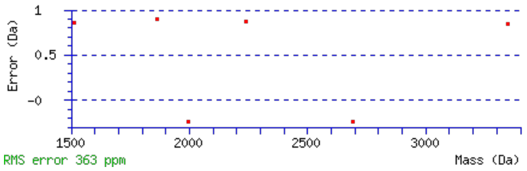

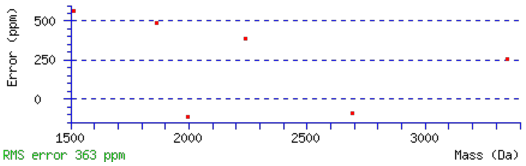

Protein View: RGA3_SOLBU

Putative disease resistance protein RGA3 OS=Solanum bulbocastanum GN=RGA3 PE=2 SV=2

**Database:**	SwissProt
**Score:**	74
**Expect:**	0.0014
**Nominal mass (M_r):**	114864
**Calculated pI:**	6.03
**Taxonomy:**	Solanum bulbocastanum

Sequence similarity is available as an NCBI BLAST search of RGA3_SOLBU against nr.

Search parameters

**Enzyme:**	Trypsin: cuts C-term side of KR unless next residue is P.
**Fixed modifications:**	Carbamidomethyl (C)
**Variable modifications:**	Oxidation (M)
**Mass values searched:**	10
**Mass values matched:**	10

Protein sequence coverage: 13%

Matched peptides shown in ***bold red***.

```
  1 MAEAFLQVLL DNLTFFIQGE LGLVFGFEKE FKKLSSMFSM IQAVLEDAQE
 51 KQLKYKAIKN WLQKLNVAAY EVDDILDDCK TEAARFKQAV LGRYHPRTIT
101 FCYKVGKRMK EMMEKLDAIA EERRNFHLDE RIIERQAARR QTGFVLTEPK
151 VYGREKEEDE IVKILINNVS YSEEVPVLPI LGMGGLGKTT LAQMVFNDQR
201 ITEHFNLKIW VCVSDDFDEK RLIKAIVESI EGKSLGDMDL APLQKKLQEL
251 LNGKRYFLVL DDVWNEDQEK WDNLRAVLKI GASGASILIT TRLEKIGSIM
301 GTLQLYQLSN LSQEDCWLLF KQRAFCHQTE TSPKLMEIGK EIVKKCGGVP
351 LAAKTLGGLL RFKREESEWE HVRDSEIWNL PQDENSVLPA LRLSYHHLPL
401 DLRQCFAYCA VFPKDTKIEK EYLIALWMAH SFLLSKGNME LEDVGNEVWN
451 ELYLRSFFQE IEVKSGKTYF KMHDLIHDLA TSMFSASASS RSIRQINVKD
501 DEDMMFIVTN YKDMMSIGFS EVVSSYSPSL FKRFVSLRVL NLSNSEFEQL
551 PSSVGDLVHL RYLDLSGNKI CSLPKRLCKL QNLQTLDLYN CQSLSCLPKQ
601 TSKLCSLRNL VLDHCPLTSM PPRIGLLTCL KTLGYFVVGE RKGYQLGELR
651 NLNLRGAISI THLERVKNDM EAKEANLSAK ANLHSLSMSW DRPNRYESEE
701 VKVLEALKPH PNLKYLEIID FCGFCLPDWM NHSVLKNVVS ILISGCENCS
751 CLPPFGELPC LESLELQDGS VEVEYVEDSG FLTRRRFPSL RKLHIGGFCN
801 LKGLQRMKGA EQFPVLEEMK ISDCPMFVFP TLSSVKKLEI WGEADAGGLS
851 SISNLSTLTS LKIFSNHTVT SLLEEMFKNL ENLIYLSVSF LENLKELPTS
901 LASLNNLKCL DIRYCYALES LPEEGLEGLS SLTELFVEHC NMLKCLPEGL
951 QHLTTLTSLK IRGCPQLIKR CEKGIGEDWH KISHIPNVNI YI
```

Unformatted sequence string: 992 residues (for pasting into other applications).

Sort peptides by ◉ Residue Number ○ Increasing Mass ○ Decreasing Mass

Show predicted peptides also

Start – End	Observed	Mr(expt)	Mr(calc)	Delta	M	Peptide
33 – 51	2170.0869	2169.0796	2170.0650	-0.9853	1	K.KLSSMFSMIQAVLEDAQEK.Q + Oxidation (M)
34 – 51	2042.0170	2041.0097	2041.9700	-0.9603	0	K.LSSMFSMIQAVLEDAQEK.Q + Oxidation (M)
111 – 123	1596.7328	1595.7255	1595.7171	0.0085	1	K.EMMEKLDAIAEER.R + 2 Oxidation (M)
189 – 200	1439.6387	1438.6314	1438.6875	-0.0560	0	K.TTLAQMVFNDQR.I + Oxidation (M)
225 – 245	2212.9888	2211.9815	2213.1613	-1.1798	1	K.AIVESIEGKSLGDMDLAPLQK.K
355 – 363	1004.5207	1003.5134	1003.6178	-0.1045	1	K.TLGGLLRFK.R
393 – 403	1363.5565	1362.5493	1362.7408	-0.1915	0	R.LSYHHLPLDLR.Q
513 – 532	2227.0063	2225.9990	2226.0225	-0.0234	0	K.DMMSIGFSEVVSSYSPSLFK.R + Oxidation (M)
577 – 599	2809.2717	2808.2644	2808.3972	-0.1328	1	R.LCKLQNLQTLDLYNCQSLSCLPK.Q
666 – 673	933.5141	932.5068	933.4589	-0.9521	1	R.VKNDMEAK.E

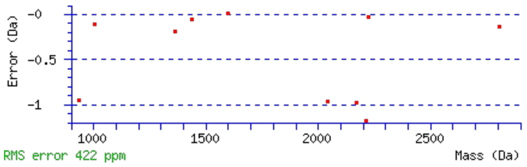

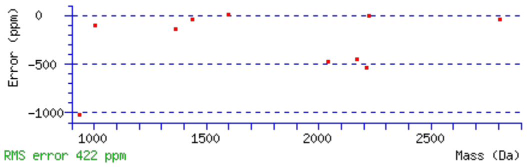

Protein View: RBOHJ_ARATH

Putative respiratory burst oxidase homolog protein J OS=Arabidopsis thaliana GN=RBOHJ PE=3 SV=2

**Database:**	SwissProt
**Score:**	60
**Expect:**	0.04
**Monoisotopic mass (M_r):**	103498
**Calculated pI:**	9.48
**Taxonomy:**	Arabidopsis thaliana

Sequence similarity is available as an NCBI BLAST search of RBOHJ_ARATH against nr.

Search parameters

**Enzyme:**	Trypsin: cuts C-term side of KR unless next residue is P.
**Fixed modifications:**	Carbamidomethyl (C)
**Variable modifications:**	Oxidation (M)
**Mass values searched:**	7
**Mass values matched:**	7

Protein sequence coverage: 9%

Matched peptides shown in ***bold red***.

1 **MKNNK**KVGTE DSTKWMLESV EIDPKGDSSV KQPESTINSN NPESSGAGGG
51 ILKNVSKNLA VGSIIR**SMSV** **NK**WRKSGNLG SPSTRKSGNL GPPLPVSQVK
101 RPGPQRVERT TSSAARGLQS LRFLDRTVTG RERDSWRSIE NRFNQFAVDG
151 RLPKDKFGVC IGMGDTLEFA AKVYEALGRR RQIKTENGID KEQLKLFWED
201 MIKKDLDCRL QIFFDMCDKD GDGKLTEEEV KEVIVLSASA NRLVNLKKNA
251 ASYASLIMEE LDPNEQGYIE MWQLEVLLTG IVSNADSHKV VR**KSQQLTR**A
301 MIPKRYRTPT SKYVVVTAEL MYEHWKK**IWV** **VTLWLAVNVV** **LFMWK**YEEFT
351 TSPLYNITGR CLCAAKGTAE ILK**LNMALIL** **VPVLR**RTLTF LRSTFLNHLI
401 PFDDNINFHK LIAVAIAVIS LLHTALHMLC NYPRLSSCPY NFYSDYAGNL
451 LGAKQPTYLG LMLTPVSVTG VLMIIFMGIS FTLAMHYFRR NIVKLPIPFN
501 RLAGFNSFWY AHHLLVIAYA LLIIHGYILI IEKPWYQKTT WMYVAIPMVL
551 YASERLFSRV QEHNHR**VHII** **KAIVYSGNVL** **ALYMTKPQGF** **K**YKSGMYMFV
601 KCPDISKFEW HPFSITSAPG DEYLSVHIR**A** **LGDWTSELRN** **R**FAETCEPHQ
651 KSKPSPNDLI RMETRARGAN PHVEESQALF PRIFIKGPYG APAQSYQKFD
701 ILLLIGLGIG ATPFISILKD MLNNLKPGIP KTGQKYEGSV GGESLGGSSV
751 YGGSSVNGGG SVNGGGSVSG GGRKFPQRAY FYWVTREQAS FEWFKGVMDD
801 IAVYDKTNVI EMHNYLTSMY EAGDARSALI AMVQKLQHAK NGVDIVSESR
851 IRTHFARPNW RKVFSELSNK HETSRIGVFY CGSPTLVRPL KSLCQEFSLE
901 SSTRFTFHKE NF

Unformatted sequence string: 912 residues (for pasting into other applications).

Sort by ◉ residue number ○ increasing mass ○ decreasing mass
Show ◉ matched peptides only ○ predicted peptides also

Start – End	Observed	Mr(expt)	Mr(calc)	Delta	M	Peptide
1 – 5	649.9890	648.9817	649.3217	-0.3400	1	-.MKNNK.K + Oxidation (M)
67 – 72	665.9642	664.9569	664.3214	0.6356	0	R.SMSVNK.W
293 – 299	861.0069	859.9996	859.4875	0.5120	1	R.KSQQLTR.A
328 – 345	2233.0188	2232.0115	2232.2533	-0.2418	0	K.IWVVTLWLAVNVVLFMWK.Y + Oxidation (M)
374 – 385	1352.6350	1351.6277	1350.8421	0.7856	0	K.LNMALILVPVLR.R
567 – 591	2807.1404	2806.1331	2805.5615	0.5716	1	R.VHIIKAIVYSGNVLALYMTKPQGFK.Y + Oxidation (M)
630 – 641	1417.6929	1416.6856	1416.7110	-0.0254	1	R.ALGDWTSELRNR.F

Protein View: GSTF2_ARATH

Glutathione S-transferase F2 OS=Arabidopsis thaliana GN=GSTF2 PE=1 SV=3

**Database:**	SwissProt
**Score:**	57
**Expect:**	0.082
**Monoisotopic mass (M_r):**	24114
**Calculated pI:**	5.92
**Taxonomy:**	Arabidopsis thaliana

Sequence similarity is available as an NCBI BLAST search of GSTF2_ARATH against nr.

Search parameters

**Enzyme:**	Trypsin: cuts C-term side of KR unless next residue is P.
**Fixed modifications:**	Carbamidomethyl (C)
**Variable modifications:**	Oxidation (M)
**Mass values searched:**	4
**Mass values matched:**	4

Protein sequence coverage: 23%

Matched peptides shown in ***bold red***.

```
  1 MAGIKVFGHP ASIATRRVLI ALHEKNLDFE LVHVELKDGE HKKEPFLSRN
 51 PFGQVPAFED GDLKLFESRA ITQYIAHRYE NQGTNLLQTD SKNISQYAIM
101 AIGMQVEDHQ FDPVASKLAF EQIFKSIYGL TTDEAVVAEE EAKLAKVLDV
151 YEARLKEFKY LAGETFTLTD LHHIPAIQYL LGTPTKKLFT ERPRVNEWVA
201 EITKRPASEK VQ
```

Unformatted sequence string: 212 residues (for pasting into other applications).

Sort by ◉ residue number ○ increasing mass ○ decreasing mass
Show ◉ matched peptides only ○ predicted peptides also

Start	– End	Observed	Mr(expt)	Mr(calc)	Delta	M	Peptide
43	– 49	877.0433	876.0361	875.4865	0.5496	1	K.KEPFLSR.N
93	– 117	2807.3358	2806.3285	2807.3258	-0.9973	0	K.NISQYAIMAIGMQVEDHQFDPVASK.L + Oxidation (M)
187	– 194	1045.5375	1044.5302	1045.6032	-1.0731	1	K.KLFTERPR.V
195	– 204	1187.6630	1186.6557	1187.6186	-0.9629	0	R.VNEWVAEITK.R

Protein View: PALY_VITVI

Phenylalanine ammonia-lyase (Fragment) OS=Vitis vinifera GN=PAL PE=2 SV=1

**Database:**	SwissProt
**Score:**	73
**Expect:**	0.0017
**Nominal mass (M_r):**	46443
**Calculated pI:**	6.12
**Taxonomy:**	Vitis vinifera

Sequence similarity is available as an NCBI BLAST search of PALY_VITVI against nr.

Search parameters

**Enzyme:**	Trypsin: cuts C-term side of KR unless next residue is P.
**Fixed modifications:**	Carbamidomethyl (C)
**Variable modifications:**	Oxidation (M)
**Mass values searched:**	8
**Mass values matched:**	7

Protein sequence coverage: 28%

Matched peptides shown in ***bold red***.

```
  1 TDHLTHKLKH HPGQIEAAAI MEHILDGSSY VKEAKKLHEM DPLQKPKQDR
 51 YALRTSPQWL GPHIEVIRAS TKSIEREINS VNDNPLIDVS RNKALHGGNF
101 QGTPIGVSMD NTRLAIAAIG KLMFAQFSEL VNDFYNNGLP SNLSGSRNPS
151 LDYGFKGAEI AMASYCSELQ FLANPVTNHV ESAEQHNQDV NSLGLISSRK
201 TAEAVDILKL MSTTYLVALC QAIDLRHLEE NLKSTVKKTV SHVAKKTLTI
251 GANGELHPSR FCEKDLLKVV DREHVFAYID DPCSATYPLM QKVRQVLVEH
301 ALNNGESEKN GSTSIFQKIG AFEEELKAVL PKEVESARDG VESGNPSIPN
351 RIKECRSYPL YKFVREELGT GLLTGEKVRS PGEDFDKVFT AMCEGKIIDP
401 LLDCLSAWNG APLPIC
```

Unformatted sequence string: 416 residues (for pasting into other applications).

Sort peptides by (●) Residue Number ( ) Increasing Mass ( ) Decreasing Mass

Show predicted peptides also

Start – End	Observed	Mr(expt)	Mr(calc)	Delta	M	Peptide
37 – 50	1750.8183	1749.8110	1749.8832	-0.0722	1	K.LHEMDPLQKPKQDR.Y + Oxidation (M)
55 – 68	1631.6873	1630.6800	1631.8784	-1.1984	0	R.TSPQWLGPHIEVIR.A
73 – 91	2170.0937	2169.0864	2169.1025	-0.0161	1	K.SIEREINSVNDNPLIDVSR.N
94 – 121	2808.2997	2807.2924	2808.4705	-1.1781	1	K.ALHGGNFQGTPIGVSMDNTRLAIAAIGK.L
273 – 292	2385.0064	2383.9991	2384.0817	-0.0825	0	R.EHVFAYIDDPCSATYPLMQK.V
363 – 377	1648.7257	1647.7184	1647.8832	-0.1648	1	K.FVREELGTGLLTGEK.V
378 – 387	1149.6462	1148.6390	1148.5462	0.0928	1	K.VRSPGEDFDK.V

No match to: 2807.3021

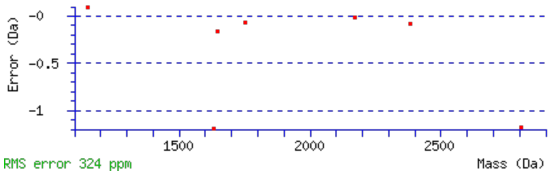

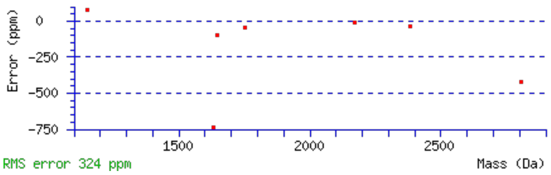

Protein View: CAMT1_POPTR

Caffeoyl-CoA O-methyltransferase 1 OS=Populus trichocarpa GN=CCOAOMT1 PE=2 SV=1

**Database:**	SwissProt
**Score:**	73
**Expect:**	0.0021
**Monoisotopic mass (M_r):**	28017
**Calculated pI:**	5.30
**Taxonomy:**	Populus trichocarpa

Sequence similarity is available as an NCBI BLAST search of CAMT1_POPTR against nr.

Search parameters

**Enzyme:**	Trypsin: cuts C-term side of KR unless next residue is P.
**Fixed modifications:**	Carbamidomethyl (C)
**Variable modifications:**	Oxidation (M)
**Mass values searched:**	5
**Mass values matched:**	5

Protein sequence coverage: 34%

Matched peptides shown in ***bold red***.

```
  1 MATNGEEQQS QAGRHQEVGH KSLLQSDALY QYILETSVYP REPECMKELR
 51 EVTAKHPWNI MTTSADEGQF LNMLLKLVNA KNTMEIGVYT GYSLLATALA
101 IPEDGKILAM DINRENYELG LPVIQKAGVA HKIDFKEGPA LPVLDQMIED
151 GKCHGSFDFI FVDADKDNYI NYHKRLIELV KVGGLIGYDN TLWNGSVVAP
201 PDAPMRKYVR YYRDFVLELN KALAADPRIE ICMLPVGDGI TLCRRIQ
```

Unformatted sequence string: 247 residues (for pasting into other applications).

Sort by ◉ residue number ○ increasing mass ○ decreasing mass
Show ◉ matched peptides only ○ predicted peptides also

Start – End	Observed	Mr(expt)	Mr(calc)	Delta	M	Peptide
1 – 14	1507.6841	1506.6769	1505.6528	1.0240	0	-.MATNGEEQQSQAGR.H
2 – 21	2191.9534	2190.9461	2190.0162	0.9299	1	M.ATNGEEQQSQAGRHQEVGHK.S
56 – 81	2970.3391	2969.3318	2970.5095	-1.1777	1	K.HPWNIMTTSADEGQFLNMLLKLVNAK.N
137 – 166	3349.5132	3348.5059	3349.5635	-1.0576	1	K.EGPALPVLDQMIEDGKCHGSFDFIFVDADK.D
167 – 174	1066.0276	1065.0203	1065.4879	-0.4676	0	K.DNYINYHK.R

Protein View: AGO1B_ORYSJ

Protein argonaute 1B OS=Oryza sativa subsp. japonica GN=AGO1B PE=2 SV=3

**Database:**	SwissProt
**Score:**	58
**Expect:**	0.055
**Nominal mass (M_r):**	124427
**Calculated pI:**	9.55
**Taxonomy:**	Oryza sativa Japonica Group

Sequence similarity is available as an NCBI BLAST search of AGO1B_ORYSJ against nr.

Search parameters

**Enzyme:**	Trypsin: cuts C-term side of KR unless next residue is P.
**Fixed modifications:**	Carbamidomethyl (C)
**Variable modifications:**	Oxidation (M)
**Mass values searched:**	8
**Mass values matched:**	8

Protein sequence coverage: 9%

Matched peptides shown in ***bold red***.

```
   1 MALQLENGRP HHHQVPIMVK KKRTGSGSTG ESSGEAPGAP GHGSSQRAER
  51 GPQQHGGGRG WVPQHGGRGG GQYQGRGGHY QGRGGQGSHH PGGGPPEYQG
 101 RGGPGSHHPG GGPPDYQGRG GSGSHHPGGG PPEYQPRDYQ GRGGPRPRGG
 151 MPQPYYGGPR GSGGRSVPSG SSRTVPELHQ APHVQYQAPM VSPTPSGAGS
 201 SSQPAAEVSS GQVQQQFQQL ATRDQSSTSQ AIQIAPPSSK SVRFPLRPGK
 251 GTYGDRCIVK ANHFFAELPD KDLHQYDVSI TPEVTSRGVN RAVMFELVTL
 301 YRYSHLGGRL PAYDGRKSLY TAGPLPFASR TFEITLQDEE DSLGGGQGTQ
 351 RRERLFRVVI KFAARADLHH LAMFLAGRQA DAPQEALQVL DIVLRELPTT
 401 RYSPVGRSFY SPNLGRRQQL GEGLESWRGF YQSIRPTQMG LSLNIDMSST
 451 AFIEPLPVID FVAQLLNRDI SVRPLSDSDR VKIKKALRGV KVEVTHRGNM
 501 RRKYRISGLT SQATRELSFP VDDRGTVKTV VQYFLETYGF SIQHTTLPCL
 551 QVGNQQRPNY LPMEVCKIVE GQRYSKRLNE KQITALLKVT CQRPQERELD
 601 ILRTVSHNAY HEDQYAQEFG IKIDERLASV EARVLPPPRL KYHDSGREKD
 651 VLPRVGQWNM MNKKMVNGGR VNNWACINFS RNVQDSAARG FCHELAIMCQ
 701 ISGMDFALEP VLPPLTARPE HVERALKARY QDAMNMLRPQ GRELDLLIVI
 751 LPDNNGSLYG DLKRICETDL GLVSQCCLTK HVFKMSKQYL ANVALKINVK
 801 VGGRNTVLVD ALTRRIPLVS DRPTIIFGAD VTHPHPGEDS SPSIAAVVAS
 851 QDWPEVTKYA GLVSAQAHRQ ELIQDLFKVW QDPHRGTVTG GMIKELLISF
 901 KRATGQKPQR IIFYRDGVSE GQFYQVLLYE LDAIRKACAS LEPNYQPPVT
 951 FVVVQKRHHT RLFANNHNDQ RTVDRSGNIL PGTVVDSKIC HPTEFDFYLC
1001 SHAGIQGTSR PAHYHVLWDE NKFTADELQT LTNNLCYTYA RCTRSVSIVP
1051 PAYYAHLAAF RARFYMEPET SDSGSMASGA ATSRGLPPGV RSARVAGNVA
1101 VRPLPALKEN VKRVMFYC
```

Unformatted sequence string: 1118 residues (for pasting into other applications).

Sort peptides by ◉ Residue Number ○ Increasing Mass ○ Decreasing Mass

Show predicted peptides also

Start – End	Observed	Mr(expt)	Mr(calc)	Delta	M	Peptide
2 – 20	2203.1298	2202.1225	2203.1796	-1.0571	0	M.ALQLENGRPHHHQVPIMVK.K
51 – 68	1868.8823	1867.8750	1866.9099	0.9651	1	R.GPQQHGGGRGWVPQHGGR.G
149 – 160	1278.6486	1277.6413	1278.5815	-0.9403	0	R.GGMPQPYYGGPR.G
379 – 401	2576.2931	2575.2858	2575.3969	-0.1112	1	R.QADAPQEALQVLDIVLRELPTTR.Y
498 – 502	650.0395	649.0322	648.3126	0.7197	1	R.GNMRR.K + Oxidation (M)
665 – 681	1995.0070	1993.9997	1993.9363	0.0634	1	K.MVNGGRVNNWACINFSR.N
682 – 689	861.0694	860.0622	859.4148	0.6474	0	R.NVQDSAAR.G

Start – End	Observed	Mr(expt)	Mr(calc)	Delta	M	Peptide
781 – 787	877.0429	876.0357	875.4687	0.5669	1	K.HVFKMSK.Q

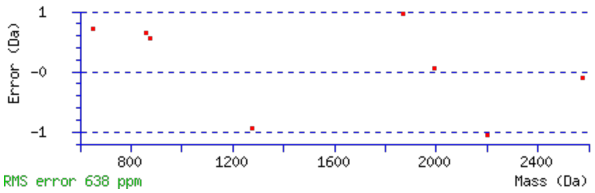

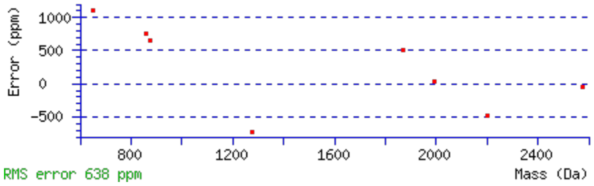

MASCOT Search Results

Protein View: ARP_ARATH

DNA-(apurinic or apyrimidinic site) lyase, chloroplastic OS=Arabidopsis thaliana GN=ARP PE=1 SV=2

**Database:**	SwissProt
**Score:**	54
**Expect:**	0.16
**Monoisotopic mass (M_r):**	60622
**Calculated pI:**	9.11
**Taxonomy:**	Arabidopsis thaliana

Sequence similarity is available as an NCBI BLAST search of ARP_ARATH against nr.

Search parameters

**Enzyme:**	Trypsin: cuts C-term side of KR unless next residue is P.
**Fixed modifications:**	Carbamidomethyl (C)
**Variable modifications:**	Oxidation (M)
**Mass values searched:**	5
**Mass values matched:**	5

Protein sequence coverage: 12%

Matched peptides shown in ***bold red***.

1 **MNNVLQFGLQ SSAIYVAKFL VVPLR**SLRVG SSFVGVGVGT RSFNKRLMSN
51 ATAFSINNSK RKELKIPGAA IDQNCHQMGS DTDRDEMGTL QDDRK**EIEAM**
101 **TVQELRSTLR** KLGVPVKGRK QELISTLRLH MDSNLPDQKE TSSSTRSDSV
151 TIKRKISNRE EPTEDECTNS EAYDIEHGEK RVKQSTEKNL KAKVSAKAIA
201 KEQKSLMRTG KQQIQSKEET SSTISSELLK TEEIISSPSQ SEPWTVLAHK
251 KPQKDWKAYN PKTMRPPPLP EGTKCVKVMT WNVNGLRGLL KFESFSALQL
301 AQRENFDILC LQETKLQVK**D VEEIKK**TLID GYDHSFWSCS VSKLGYSGTA
351 IISRIKPLSV RYGTGLSGHD TEGRIVTAEF DSFYLINTYV PNSGDGLKRL
401 SYRIEEWDRT LSNHIKELEK SKPVVLTGDL NCAHEEIDIF NPAGNKRSAG
451 FTIEERQSFG ANLLDKGFVD TFR**KQHPGVV GYTYWGYRHG GR**KTNKGWRL
501 DYFLVSQSIA ANVHDSYILP DINGSDHCPI GLILKL

Unformatted sequence string: 536 residues (for pasting into other applications).

Sort by ◉ residue number ○ increasing mass ○ decreasing mass
Show ◉ matched peptides only ○ predicted peptides also

Start – End	Observed	Mr(expt)	Mr(calc)	Delta	M	Peptide

1	– 25	2807.1453	2806.1381	2806.5568	-0.4187	1	-.MNNVLQFGLQSSAIYVAKFLVVPLR.S
96	– 110	1776.7130	1775.7057	1774.9247	0.7810	1	K.EIEAMTVQELRSTLR.K
320	– 326	861.0047	859.9974	859.4651	0.5324	1	K.DVEEIKK.T
474	– 488	1809.7996	1808.7924	1809.8951	-1.1027	1	R.KQHPGVVGYTYWGYR.H
475	– 492	2089.0374	2088.0301	2089.0031	-0.9730	1	K.QHPGVVGYTYWGYRHGGR.K

MASCOT Search Results

Protein View: PP292_ARATH

Pentatricopeptide repeatcontaining protein At3g61520, mitochondrial OS=Arabidopsis thaliana GN=At3g61520 PE=2 SV=1

Database:	SwissProt
Score:	40
Expect:	4.1
Nominal mass (M_r):	87762
Calculated pI:	6.86
Taxonomy:	Arabidopsisthaliana

Sequence similarity is available as anNCBIBLASTsearchofPP292_ARATHagainstnr.

Search parameters

MS data file:	peaklist.xml
Enzyme:	Trypsin: cuts Cterm side of KR unless next residue is P.
Fixed modifications:	Carbamidomethyl(C)
Variable modifications:	Oxidation(M)
Mass values searched:	15
Mass values matched:	9

Protein sequence coverage: 16%

Matched peptides shown in **bold red**.

```
  1 MSIMLSISRR RNSYILLNHS RFLRRFSYDV DPRPEIKLES QEFVVVKFVK
 51 TLQKTPQHDW ASSESLSALV VSSSSASPLV FSQITRRLGS YSLAISFFEY
101 LDAKSQSLKR REESLSLALQ SVIEFAGSEP DPRDKLLRLY EIAKEKNIPL
151 TVVATNLLIR WFGRMGMVNQ SVLVYERLDS NMKNSQVRNV VVDVLLRNGL
201 VDDAFKVLDE MLQKESVFPP NRITADIVLH EVWKGRLLTE EKIIALISRF
251 SSHGVSPNSV WLTRFISSLC KNARANAAWD ILSDLMKNKT PLEAPPFNAL
301 LSCLGRNMDI SRMNDLVLKM DEVKIRPDVV TLGILINTLC KSRRVDEALE
351 VFEKMRGKRT DDGNVIKADS IHFNTLIDGL CKVGRLKEAE ELLVRMKLEE
401 RCAPNAVTYN CLIDGYCRAG KLETAKEVVS RMKEDEIKPN VVTVNTIVGG
451 MCRHHGLNMA VVFFMDMEKE GVKGNVVTYM TLIHACCSVS NVEKAMYWYE
501 KMLEAGCSPD AKIYYALISG LCQVRRDHDA IRVVEKLKEG GFSLDLLAYN
551 MLIGLFCDKN NTEKVYEMLT DMEKEGKKPD SITYNTLISF FGKHKDFESV
601 ERMMEQMRED GLDPTVTTYG AVIDAYCSVG ELDEALKLFK DMGLHSKVNP
651 NTVIYNILIN AFSKLGNFGQ ALSLKEEMKM KMVRPNVETY NALFKCLNEK
701 TQGETLLKLM DEMVEQSCEP NQITMEILME RLSGSDELVK LRKFMQGYSV
751 ASPTEKASPF DVFSLG
```

Unformatted sequence string: 766 residues (for pasting into other applications).

Sort peptides by ● Residue Number ● Increasing Mass ● Decreasing Mass

Show predicted peptides also

Start - End	Observed	Mr(expt)	Mr(calc)	Delta	M	Peptide
165 - 177	1558.7158	1557.7085	1556.7327	0.9758	0	R.MGMVNQSVLVYER.L + 2 Oxidation (M)
165 - 183	2244.9744	2243.9671	2245.0541	1.0870	1	R.MGMVNQSVLVYERLDSNMK.N + 2 Oxidation (M)
178 - 188	1308.5549	1307.5477	1306.6299	0.9177	1	R.LDSNMKNSQVR.N + Oxidation (M)
250 - 271	2508.2163	2507.2090	2508.2584	1.0493	1	R.FSSHGVSPNSVWLTRFISSLCK.N
290 - 306	1855.7063	1854.6990	1854.9662	0.2672	0	K.TPLEAPPFNALLSCLGR.N
360 - 367	860.9733	859.9661	860.4240	0.4579	0	R.TDDGNVIK.A
560 - 574	1877.6882	1876.6810	1875.8230	0.8580	1	K.NNTEKVYEMLTDMEK.E + 2 Oxidation (M)
596 - 608	1703.7587	1702.7514	1702.7113	0.0401	1	K.DFESVERMMEQMR.E + Oxidation (M)
641 - 664	2704.1660	2703.1587	2703.4054	0.2467	1	K.DMGLHSKVNPNTVIYNILINAFSK.L + Oxidation (M)

No match to: 1065.9686, 1278.5675, 1514.7343, 1721.7703, 2672.1465, 2808.1155

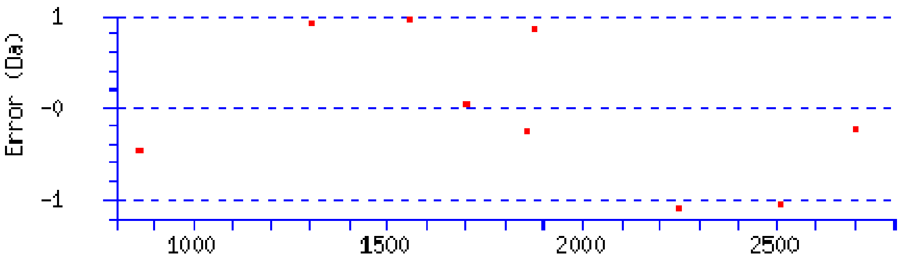

RMS error 449 ppm

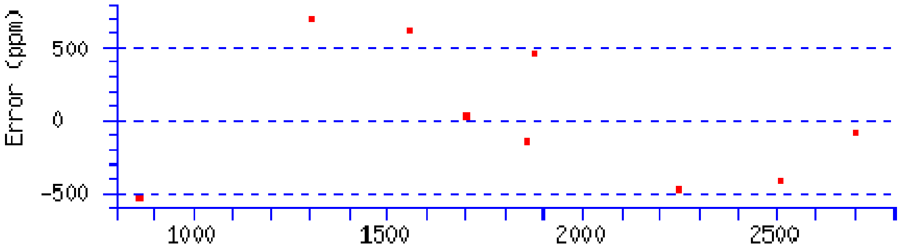

RMS error 449 ppm

Protein View: MGN_ORYSJ

Protein mago nashi homolog OS=Oryza sativa subsp. japonica GN=Os12g0287200 PE=2 SV=2

**Database:**	SwissProt
**Score:**	60
**Expect:**	0.037
**Nominal mass (M_r):**	18475
**Calculated pI:**	5.84
**Taxonomy:**	Oryza sativa Japonica Group

Sequence similarity is available as an NCBI BLAST search of MGN_ORYSJ against nr.

Search parameters

**Enzyme:**	Trypsin: cuts C-term side of KR unless next residue is P.
**Fixed modifications:**	Carbamidomethyl (C)
**Variable modifications:**	Oxidation (M)
**Mass values searched:**	4
**Mass values matched:**	4

Protein sequence coverage: 26%

Matched peptides shown in ***bold red***.

```
  1 MATGGAAGED VPGGGEFYLR YYVGHKGKFG HEFLEFEFRP DGKLRYANNS
 51 NYKNDTMIRK EVFVSPSVLR EARRIIQESE IMKEDDNNWP EPDRVGRQEL
101 EIVMGNEHIS FTTSKIGSLV DVQTSKDPEG LRIFYYLVQD LKCFVFSLIN
151 LHFKIKPIQS
```

Unformatted sequence string: 160 residues (for pasting into other applications).

Sort peptides by ◉ Residue Number ○ Increasing Mass ○ Decreasing Mass

Show predicted peptides also

Start – End	Observed	Mr(expt)	Mr(calc)	Delta	M	Peptide
74 – 83	1263.6218	1262.6145	1261.6700	0.9445	1	R.RIIQESEIMK.E + Oxidation (M)
75 – 83	1105.4960	1104.4887	1105.5689	-1.0802	0	R.IIQESEIMK.E + Oxidation (M)
75 – 94	2457.2313	2456.2240	2457.1118	-0.8878	1	R.IIQESEIMKEDDNNWPEPDR.V
133 – 154	2807.2901	2806.2828	2806.4920	-0.2092	1	R.IFYYLVQDLKCFVFSLINLHFK.I

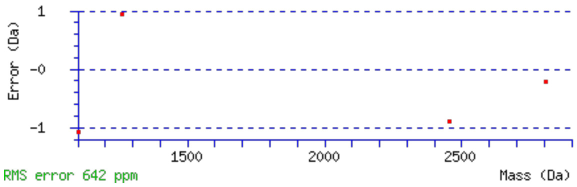

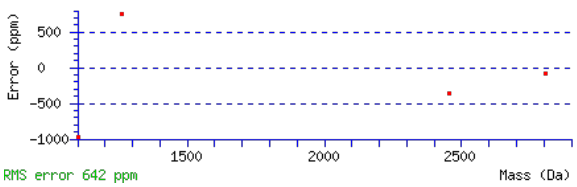

Protein View: LBD26_ARATH

LOB domain-containing protein 26 OS=Arabidopsis thaliana GN=LBD26 PE=2 SV=2

**Database:**	SwissProt
**Score:**	66
**Expect:**	0.0076
**Nominal mass (M_r):**	17931
**Calculated pI:**	9.39
**Taxonomy:**	Arabidopsis thaliana

Sequence similarity is available as an NCBI BLAST search of LBD26_ARATH against nr.

Search parameters

**Enzyme:**	Trypsin: cuts C-term side of KR unless next residue is P.
**Fixed modifications:**	Carbamidomethyl (C)
**Variable modifications:**	Oxidation (M)
**Mass values searched:**	4
**Mass values matched:**	4

Protein sequence coverage: 50%

Matched peptides shown in ***bold red***.

```
  1 MNANPCEVCR FQNKQCVNNC MFALLFPSSD LEKFDVVNRI FGLETLTFYL
 51 KDLSPMERID TTRTLYYEAK PCFLNPPKNP SKFLEALLNY PYQKAEEVSK
101 TKKLLASYSR PCVVLALPAP KYTQSKSKPS VLRKRKRKTK SSDESAIRVV
151 EDS
```

Unformatted sequence string: 153 residues (for pasting into other applications).

Sort peptides by ◉ Residue Number ○ Increasing Mass ○ Decreasing Mass

Show predicted peptides also

Start - End	Observed	Mr(expt)	Mr(calc)	Delta	M	Peptide
11 - 33	2807.2033	2806.1961	2805.2924	0.9036	1	R.FQNKQCVNNCMFALLFPSSDLEK.F + Oxidation (M)
40 - 51	1445.6099	1444.6026	1443.8013	0.8013	0	R.IFGLETLTFYLK.D
64 - 82	2268.0405	2267.0332	2266.1456	0.8876	1	R.TLYYEAKPCFLNPPKNPSK.F
104 - 126	2562.1655	2561.1582	2561.4039	-0.2457	1	K.LLASYSRPCVVLALPAPKYTQSK.S

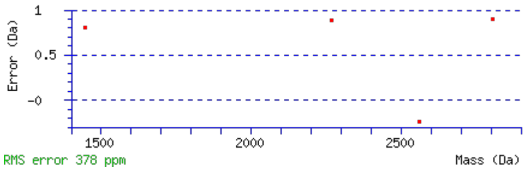

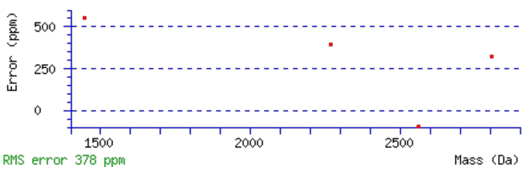

MATRIX SCIENCE MASCOT Search Results

Protein View: SNL6_ARATH

Paired amphipathic helix protein Sin3-like 6 OS=Arabidopsis thaliana GN=SNL6 PE=3 SV=2

**Database:**	SwissProt
**Score:**	71
**Expect:**	0.0031
**Monoisotopic mass (M_r):**	134332
**Calculated pI:**	7.17
**Taxonomy:**	Arabidopsis thaliana

Sequence similarity is available as an NCBI BLAST search of SNL6_ARATH against nr.

Search parameters

**Enzyme:**	Trypsin: cuts C-term side of KR unless next residue is P.
**Mass values searched:**	7
**Mass values matched:**	7

Protein sequence coverage: 12%

Matched peptides shown in ***bold red***.

1 MRDLLLGTTI PSYFSWLGLF PRFLVWFGFL KASFFCSSRN QSAGESGRRL
51 KMKRAREDVH TDTQKRKPEV SSRGETNKLP RTIDALTYLK AVKDIFHDNK
101 EKYESFLELM KEFKAQTIDT NGVIERIKVL FKGYRDLLLG FNTFLPKGYK
151 ITLLPEEEKP KIRVDFKDAI GFVTKIKTRF GDDEHAYKRF LDILNLYRKE
201 KKSISEVYEE VTMLFKGHED LLMEFVNFLP NCPESAPSTK NAVPRHKGTA
251 TTAMHSDKKR KQRCKLEDYS GHSDQREDGD ENLVTCSADS PVGEGQPGYF
301 RDYENREDTE TDTADRTEK**S** **AASGSQDIGN** **HKSTTK**YVGT PINELDLSEC
351 TQCTPSYRLL PKDYAVEIPS YRNTLGKKTL NDHLVSVTSG SEDYSFSHMR
401 KNQYEESLFR CEDDRYEMDM LLGSVSSAIK **QVEILLEKMN** **NNTISVDSTI**
451 **CIEK**HLSAMN LRCIERLYGD NGLDVMDLLK KNMHSALPVI LTRLKQKQEE
501 WARCHSDFQK VWAEVYAKNH HKSLDHRSFY FKQQDSKNLS TKCLVAEVKD
551 ISEKKHQEDL LQAIAVRVMP LFTPDLEFNY CDTQIHEDLY LLIKYYCEEI
601 CATEQSDKVM KLWITFLEPI FGILSRSQDN LALEDVSKLK NNRELQDACL
651 AVKETASGSN RKHPISPKRL SKDNTKMQGS SSREDVSANI KVKTAQPDK**L**
701 **QDDAAMTNEV** **IQSSK**FVSPK NDQIMEDEGN HMVNAASVEK HELEEGELSP
751 TASREQSNFE VNGQNAFKPL QKVTDNVRSN KDKQSCDKKG AKNKTRAEDD
801 KQENCHKLSE NNKTASEMLV SGTKVSCHEE NNRVMNCNGR GSVAGEMANG
851 NQGEDGSFAF SERFLQTVKP VAKHLSWPLQ ASETCSQNDS QVFYGNDSYY
901 VLFR**LHQMLY** **ERIQTAK**KHS EKKWKAADNT TPDSYPRFMD ALYNLLDGSI
951 DNTKFEDECR AIFGAQSYVL FTLDKLVQKF VK**HLHSVASD** **ETDTKLLQLH**
1001 **AYENYR**KPGK **FFDLVYHENA** **CALLHEANIY** **RIR**YSSEGTR **LSIQLMNSGN**
1051 **NQLEVMGVAM** **EPAFADYLQN** **K**CLKSVNDEE NHGLFLNRNK KKFTSLDESR
1101 GMPVAMERLN IINEMECRMA CSSSKVKYVA NTSDLLYRSK QGKPNSRVSE
1151 ILKQRRISRF HIMLNCRLCA LPL

Unformatted sequence string: 1173 residues (for pasting into other applications).

Sort by (●) residue number ( ) increasing mass ( ) decreasing mass
Show (●) matched peptides only ( ) predicted peptides also

Start - End	Observed	Mr(expt)	Mr(calc)	Delta	M	Peptide
320 - 336	1689.8578	1688.8505	1687.8125	1.0380	1	K.SAASGSQDIGNHKSTTK.Y
431 - 454	2733.2749	2732.2676	2733.3928	-1.1252	1	K.QVEILLEKMNNNTISVDSTICIEK.H
700 - 715	1749.7694	1748.7621	1748.8251	-0.0629	0	K.LQDDAAMTNEVIQSSK.F
905 - 917	1631.6671	1630.6598	1629.8661	0.7937	1	R.LHQMLYERIQTAK.K
983 - 1006	2841.1968	2840.1895	2839.3889	0.8007	1	K.HLHSVASDETDTKLLQLHAYENYR.K
1011 - 1033	2808.2332	2807.2259	2806.4013	0.8246	1	K.FFDLVYHENACALLHEANIYRIR.Y
1041 - 1071	3424.4870	3423.4798	3424.6465	-1.1667	0	R.LSIQLMNSGNNQLEVMGVAMEPAFADYLQNK.C

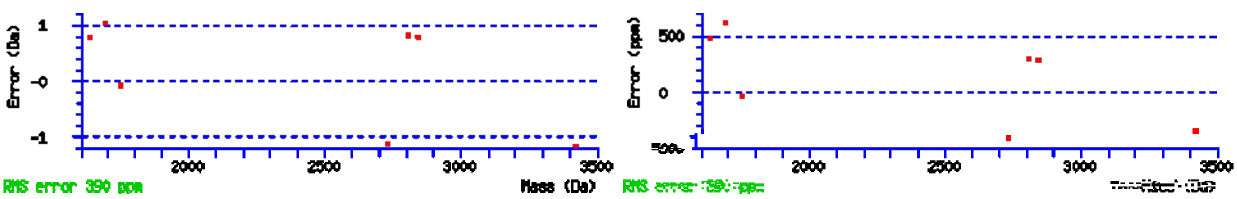

Protein View: 1433D_TOBAC

14-3-3-like protein D OS=Nicotiana tabacum PE=2 SV=1

**Database:**	SwissProt
**Score:**	53
**Expect:**	0.19
**Monoisotopic mass (M_r):**	28410
**Calculated pI:**	4.76
**Taxonomy:**	Nicotiana tabacum

Sequence similarity is available as an NCBI BLAST search of 1433D_TOBAC against nr.

Search parameters

**Enzyme:**	Trypsin: cuts C-term side of KR unless next residue is P.
**Fixed modifications:**	Carbamidomethyl (C)
**Variable modifications:**	Oxidation (M)
**Mass values searched:**	4
**Mass values matched:**	4

Protein sequence coverage: 21%

Matched peptides shown in ***bold red***.

```
  1 MAVPENLTRE QCLYLAKLAE QAERYEEMVK FMDRLVAVSA SSELTVEERN
 51 LLSVAYKNVI GSLRAAWRIV SSIEQKEEGR KNEEHVVLVK DYRSKVESEL
101 SDVCAGILKI LDQYLIPSAA AGESKVFYLK MKGDYYRYLA EFKVGNERKE
151 AAEDTMLAYK AAQDIALAEL APTHPIRLGL ALNYSVFYYE ILNASEKACS
201 MAKQAFEEAI AELDTLGEES YKDSTLIMQL LRDNLTLWTS DMQEQMDEA
```

Unformatted sequence string: 249 residues (for pasting into other applications).

Sort by ◉ residue number ○ increasing mass ○ decreasing mass
Show ◉ matched peptides only ○ predicted peptides also

Start – End	Observed	Mr(expt)	Mr(calc)	Delta	M	Peptide
126 – 132	928.3375	927.3302	927.5252	-0.1949	1	K.VFYLKMK.G
131 – 137	933.4345	932.4272	931.4222	1.0051	1	K.MKGDYYR.Y
198 – 222	2807.1607	2806.1534	2806.2677	-0.1143	1	K.ACSMAKQAFEEAIAELDTLGEESYK.D + Oxidation (M)
233 – 249	2026.9215	2025.9142	2025.8296	0.0847	0	R.DNLTLWTSDMQEQMDEA.-

Protein View: FB332_ARATH

Probable F-box protein At5g36000 OS=Arabidopsis thaliana GN=At5g36000 PE=2 SV=2

**Database:**	SwissProt
**Score:**	60
**Expect:**	0.032
**Nominal mass (M_r):**	40170
**Calculated pI:**	8.51
**Taxonomy:**	Arabidopsis thaliana

Sequence similarity is available as an NCBI BLAST search of FB332_ARATH against nr.

Search parameters

**Enzyme:**	Trypsin: cuts C-term side of KR unless next residue is P.
**Fixed modifications:**	Carbamidomethyl (C)
**Variable modifications:**	Oxidation (M)
**Mass values searched:**	21
**Mass values matched:**	10

Protein sequence coverage: 36%

Matched peptides shown in ***bold red***.

```
  1 MNTRSGDAEG DIRGKMIAPV RDGNGGQKRK LVQSNDIQRD EDGGAKRRII
 51 QSSDQKNGKI LRGIHGCVSP RCSAPTYQSR FSWYEQDIWT YITRFLDGKS
101 LVKLGATNKW FYKIAMEDTV WRFACLRDLQ VPETFPVSST WIKIYASAFD
151 GSHSYLFHQK EKHIDWMRLG AFVLDSRTSF LTESLSGRLK VPTEGTIEQM
201 LQSSGSCLIN DIKSGIWIAD LQLLRCPLCD LSTCDGTMQT LDVRHIELFL
251 SEGYKDGSWD YNLIGSHKLE KDASAACGAI FDLKHLKKSS SSGILNLKSW
301 TGEADDSQPK AVIAPHAVAV HTRLQQNEGI LVKYHTMKAG TDGDIVSIRI
351 SQQLL
```

Unformatted sequence string: 355 residues (for pasting into other applications).

Sort peptides by ◉ Residue Number ○ Increasing Mass ○ Decreasing Mass

Show predicted peptides also

Start – End	Observed	Mr(expt)	Mr(calc)	Delta	M	Peptide
49 – 56	917.5124	916.5051	917.4818	-0.9767	0	R.IIQSSDQK.N
60 – 71	1363.5619	1362.5547	1363.7507	-1.1960	1	K.ILRGIHGCVSPR.C
144 – 162	2227.0113	2226.0041	2227.0698	-1.0657	1	K.IYASAFDGSHSYLFHQKEK.H
169 – 188	2155.0537	2154.0464	2155.1273	-1.0809	1	R.LGAFVLDSRTSFLTESLSGR.L
226 – 244	2242.9970	2241.9897	2240.9534	1.0363	0	R.CPLCDLSTCDGTMQTLDVR.H
226 – 244	2258.9956	2257.9883	2256.9484	1.0399	0	R.CPLCDLSTCDGTMQTLDVR.H + Oxidation (M)
245 – 268	2807.2922	2806.2849	2807.3555	-1.0706	1	R.HIELFLSEGYKDGSWDYNLIGSHK.L
288 – 298	1132.6264	1131.6191	1132.6452	-1.0261	1	K.KSSSSGILNLK.S
289 – 298	1004.5295	1003.5223	1004.5502	-1.0279	0	K.SSSSGILNLK.S
324 – 338	1802.8683	1801.8611	1800.9556	0.9055	1	R.LQQNEGILVKYHTMK.A

No match to: 735.2685, 933.5123, 1020.5193, 1036.5148, 1381.5753, 1841.9519, 2027.0030, 2121.9908, 2212.9940, 2230.9829, 2244.9918

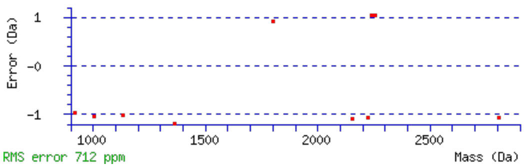

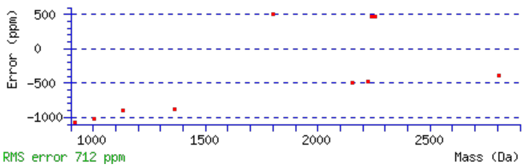

Protein View: FB169_ARATH

Putative F-box protein At3g20705 OS=Arabidopsis thaliana GN=At3g20705 PE=4 SV=1

**Database:**	SwissProt
**Score:**	69
**Expect:**	0.0046
**Nominal mass (M_r):**	26223
**Calculated pI:**	9.19
**Taxonomy:**	Arabidopsis thaliana

Sequence similarity is available as an NCBI BLAST search of FB169_ARATH against nr.

Search parameters

**Enzyme:**	Trypsin: cuts C-term side of KR unless next residue is P.
**Fixed modifications:**	Carbamidomethyl (C)
**Variable modifications:**	Oxidation (M)
**Mass values searched:**	6
**Mass values matched:**	6

Protein sequence coverage: 18%

Matched peptides shown in ***bold red***.

```
  1 MMMMSNLPND LVEEILSRVT VTFMRTVRSI CKKWNALTKD RSFTNKYIRN
 51 IAALGEREFL MIKEFSIYLV GVNLHGIQNN NFDLSIELKG KLISMDNTIR
101 RFCISQIFHC NGLFLCVSQK DMDNRLVVWN PYCSKPRWIK PSYNYRTVDR
151 FALGYDKSCG SHKILRLFGD NLNNLEIYDL SSNSWRVPNV TLERDIVYMQ
201 PGVSLKEKTY WYARDKESEN
```

Unformatted sequence string: 220 residues (for pasting into other applications).

Sort peptides by ◉ Residue Number ○ Increasing Mass ○ Decreasing Mass

Show predicted peptides also

Start – End	Observed	Mr(expt)	Mr(calc)	Delta	M	Peptide
1 – 18	2170.0873	2169.0801	2169.9778	-0.8977	0	-.MMMMSNLPNDLVEEILSR.V + 3 Oxidation (M)
2 – 18	2041.0089	2040.0016	2038.9373	1.0643	0	M.MMMSNLPNDLVEEILSR.V + 3 Oxidation (M)
33 – 39	861.0520	860.0447	859.4916	0.5532	1	K.KWNALTK.D
34 – 41	1004.5153	1003.5080	1002.5247	0.9833	1	K.WNALTKDR.S
121 – 125	650.0219	649.0146	649.2490	-0.2344	0	K.DMDNR.L
187 – 194	928.3969	927.3896	926.5185	0.8711	0	R.VPNVTLER.D

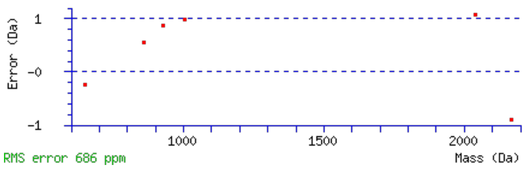

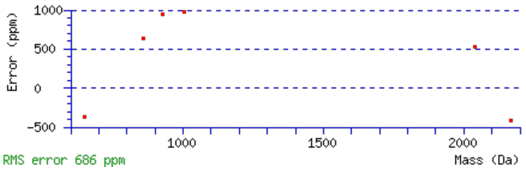

MATRIX SCIENCE MASCOT Search Results

Protein View: CRR27_ARATH

Putative cysteinerich repeat secretory protein 27 OS=Arabidopsis thaliana GN=CRRSP27 PE=5 SV=2

Database:	SwissProt
Score:	51
Expect:	0.29
Nominal mass (M_r):	29384
Calculated pI:	8.66
Taxonomy:	Arabidopsisthaliana

Sequence similarity is available as anNCBIBLAST searchofCRR27_ARATHagainstnr.

Search parameters

MS data file:	peaklist.xml
Enzyme:	Trypsin: cuts Cterm side of KR unless next residue is P.
Fixed modifications:	Carbamidomethyl(C)
Variable modifications:	Oxidation(M)
Mass values searched:	29
Mass values matched:	8

Protein sequence coverage: 38%

Matched peptides shown in **bold red**.

```
  1 MISKFGSVHI LAVVAIQLLI IPSVSSLNLT NAYLHHKCNN TEGKYSHGSA
 51 FEKYINLALR AIDSDNYLNG FAYIERGEDP NKVFVMYQCR GDSYGSKCKS
101 CISAAISGLR RRCPKSKGAV IWYDQCLFEI STINSFNKID YENDFYLSNP
151 NDVNDKELFN KETSALLEEL TNKATDKNNM IGNKFVLYAA GDKRIGTKNV
201 YAMVQCTKDL VTTTSAACFE WIFKMFSKCC EGKQGGRVLG TSCNFRYELY
251 PFLRN
```

Unformatted sequence string: 255 residues (for pasting into other applications).

Sort peptides by ● Residue Number ● Increasing Mass ● Decreasing Mass

Show predicted peptides also

Start – End	Observed	Mr(expt)	Mr(calc)	Delta	M	Peptide R.GDSYGSKCK.S
91 – 99	1001.5255	1000.5182	1000.4284	0.0898	1	K.SCISAAISGLRR.R
100 – 111	1290.5815	1289.5743	1289.6874	0.1131	1	K.IDYENDFYLSNPNDVNDKELFNK.E
139 – 161	2807.1108	2806.1036	2805.2769	0.8266	1	K.ELFNK.E
157 – 161	649.9872	648.9800	649.3435	0.3635	0	K.NNMIGNKFVLYAAGDK.R
178 – 193	1753.8175	1752.8102	1753.8821	1.0719	1	R.IGTKNVYAMVQCTK.D + Oxidation
195 – 208	1629.7625	1628.7552	1627.8062	0.9490	1	(M)
209 – 228	2398.8660	2397.8587	2397.1385	0.7202	1	K.DLVTTTSAACFEWIFKMFSK.C + Oxidation (M)

225 - 233 1163.5079 1162.5007 1161.4617 1.0390 1 K.MFSKCCEGK.Q + Oxidation (M)

No match to: 861.0013, 876.9765, 1066.0323, 1272.5970, 1415.5723, 1493.6204, 1558.6411, 1838.8251, 1915.8922, 1916.8766, 1994.8561, 2051.9375, 2101.8428, 2239.9797, 2270.0422, 2691.1355, 2841.1145, 2922.1902, 3026.3208, 3059.3765, 3349.4243

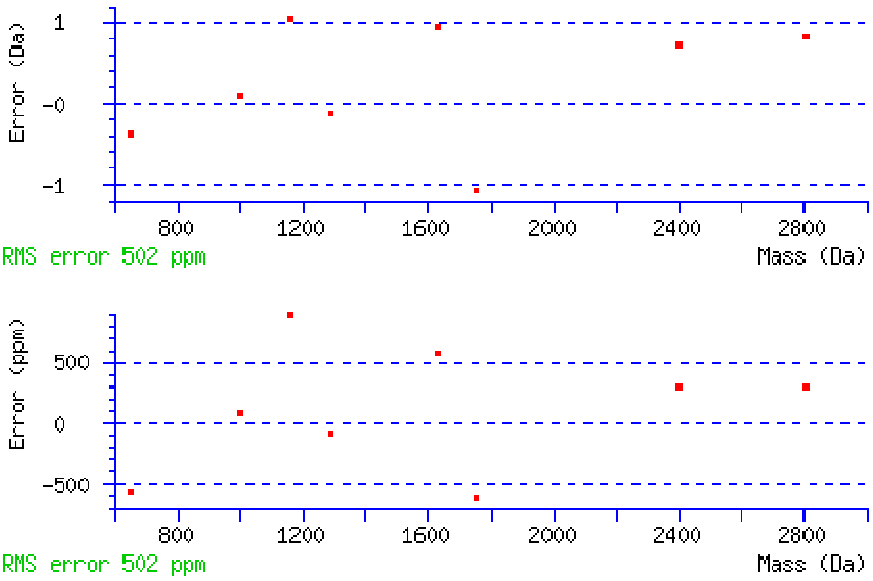

Protein View: Y4498_ARATH

Uncharacterized protein At4g04980 OS=Arabidopsis thaliana GN=At4g04980 PE=2 SV=1

**Database:**	SwissProt
**Score:**	60
**Expect:**	0.043
**Monoisotopic mass (M_r):**	82314
**Calculated pI:**	5.01
**Taxonomy:**	Arabidopsis thaliana

Sequence similarity is available as an NCBI BLAST search of Y4498_ARATH against nr.

Search parameters

**Enzyme:**	Trypsin: cuts C-term side of KR unless next residue is P.
**Fixed modifications:**	Carbamidomethyl (C)
**Variable modifications:**	Oxidation (M)
**Mass values searched:**	7
**Mass values matched:**	7

Protein sequence coverage: 12%

Matched peptides shown in ***bold red***.

```
  1 MKDLQKLSPE IVTINQSFEM EGADMDKMLI FFYEDLRAIG DSSWIMDSDWI
 51 YRSKYKNSGV GKNKSDRLVE HVLAALDGLI KTTRERFGMM DLESEGRKSF
101 TTPKGVSSEA RRSFTRSASY SESNNSFYPS PLTPRSVLPG TMMMSSNSTS
151 PSLWNLRAQA LDRLSPVDLK RFAMQILSQR DSESVSETKI GIEEENEESE
201 ILAEEKEEED NDFSVLETEG TEQEIKTEHH RESSGTEHET EAKDHSEGFE
251 TEHHRIECFE TEHEIDADDH IEDFETEHHH IEGLETEHEI DANDHIEDFE
301 TEHHHIEGFE TEHENETEDH SETTTSETDS TESSPKEDVP PPPPLTSPQT
351 PSPTVSTFNT KSSLRSQPPP PPPSPEHKAP APPPPPPMSK ASESGEFCQF
401 SKTHSTNGDN APSMPAPPAP PGSGRSLKKA TSKLRRSAQI ANLYWALKGK
451 LEGRGVEGKT KKASKGQNSV AEKSPVKVAR SGMADALAEM TKRSSYFQQI
501 EEDVQKYAKS IEELKSSIHS FQTKDMKELL EFHSKVESIL EKLTDETQVL
551 ARFEGFPEKK LEVIRTAGAL YKKLDGILVE LKNWKIEPPL NDLLDKIERY
601 FNKFKGEIET VERTKDEDAK MFKRYNINID FEVLVQVKET MVDVSSNCME
651 LALKERREAN EEAKNGEESK MKEERAKRLW RAFQFAFKVY TFAGGHDERA
701 DCLTRQLAHE IQTDPDQSES SIMS
```

Unformatted sequence string: 724 residues (for pasting into other applications).

Sort by ◉ residue number ○ increasing mass ○ decreasing mass
Show ◉ matched peptides only ○ predicted peptides also

Start – End	Observed	Mr(expt)	Mr(calc)	Delta	M	Peptide
105 – 112	861.0502	860.0429	860.4464	-0.4035	1	K.GVSSEARR.S
136 – 157	2444.1979	2443.1907	2443.1182	0.0725	0	R.SVLPGTMMMSSNSTSPSLWNLR.A + 3 Oxidation (M)
164 – 171	928.4443	927.4370	926.5549	0.8821	1	R.LSPVDLKR.F

Start – End	Observed	Mr(expt)	Mr(calc)	Delta	M	Peptide
171 – 180	1248.7468	1247.7395	1248.6761	-0.9366	1	K.RFAMQILSQR.D
474 – 480	756.3628	755.3555	755.4654	-0.1098	1	K.SPVKVAR.S
574 – 585	1428.5873	1427.5800	1426.8184	0.7616	1	K.LDGILVELKNWK.I
700 – 724	2833.1854	2832.1782	2831.2702	0.9080	1	R.ADCLTRQLAHEIQTDPDQSESSIMS.-

Protein View: M1010_ARATH

Uncharacterized mitochondrial protein AtMg01010 OS=Arabidopsis thaliana GN=AtMg01010 PE=4 SV=1

**Database:**	SwissProt
**Score:**	59
**Expect:**	0.05
**Monoisotopic mass (M_r):**	13161
**Calculated pI:**	4.59
**Taxonomy:**	Arabidopsis thaliana

Sequence similarity is available as an NCBI BLAST search of M1010_ARATH against nr.

Search parameters

**Enzyme:**	Trypsin: cuts C-term side of KR unless next residue is P.
**Fixed modifications:**	Carbamidomethyl (C)
**Variable modifications:**	Oxidation (M)
**Mass values searched:**	4
**Mass values matched:**	4

Protein sequence coverage: 30%

Matched peptides shown in ***bold red***.

```
  1 MIGGDSVEAI ERRLLAKYPE GSPSAEIIEM ARIEAEDLFE IKAQIIQRMA
 51 LYDPTGDWMA RGARALDNPR TTSGEESLER LYDIWKDLQE TGPLSDEFSR
101 LQEKVFLKKG GPGGDPIA
```

Unformatted sequence string: 118 residues (for pasting into other applications).

Sort by ◉ residue number ○ increasing mass ○ decreasing mass
Show ◉ matched peptides only ○ predicted peptides also

Start – End	Observed	Mr(expt)	Mr(calc)	Delta	M	Peptide
1 – 12	1277.1298	1276.1226	1275.6129	0.5097	0	-.MIGGDSVEAIER.R
49 – 61	1527.3106	1526.3033	1525.6694	0.6340	0	R.MALYDPTGDWMAR.G
49 – 64	1841.9581	1840.9508	1841.8189	-0.8680	1	R.MALYDPTGDWMARGAR.A + 2 Oxidation (M)
101 – 108	1004.5477	1003.5404	1003.6066	-0.0662	1	R.LQEKVFLK.K
